# Supplementary material for: Reproducible evaluation of transposable element detectors with McClintock 2 guides accurate inference of Ty insertion patterns in yeast
Source: Mob DNA. 2023 Jul 14;14:8. doi: 10.1186/s13100-023-00296-4 (PMC10347736; doi:10.1186/s13100-023-00296-4)
Supplement: Supplementary file 1 — Additional file 1. Supplemental Text, Tables and Figures. Combined PDF of all Supplemental Text, Figures, and Tables. [file 13100_2023_296_MOESM1_ESM.pdf]

Supplementary material for

Reproducible evaluation of transposable element detectors with McClintock 2 guides accurate inference of Ty insertion patterns in yeast.

Jingxuan Chen[*,1], Preston J. Basting[*,1], Shunhua Han[*], David J. Garfinkel[†] & Casey M. Bergman[*,‡,2]

[*] Institute of Bioinformatics, University of Georgia, 120 E. Green St., Athens, GA, USA.
[†] Department of Biochemistry and Molecular Biology, University of Georgia, 120 E. Green St., Athens, GA, USA.
[‡] Department of Genetics, University of Georgia, 120 E. Green St., Athens, GA, USA.

[1] These authors contributed equally to this work.
[2] Author for correspondence: cbergman@uga.edu.

Supplementary Text

McClintock 2 cross-validation and performance evaluation simulation methods

We conducted four simulation experiments to validate aspects of the McClintock re-implementation, demonstrate the utility of our new reproducible simulation framework, and evaluate the ability of McClintock components to predict non-reference TE insertions in *S. cerevisiae*. In all four simulations, synthetic genomes were created with the new reproducible simulation framework available in McClintock 2 (see Implementation in main text), the UCSC sacCer2 version of the *S. cerevisiae* S288c reference genome (to allow cross-validation with results in Nelson *et al.* [1]), and 5-bp TSDs for all Ty families [1, 2, 3, 4, 5]. All simulations and McClintock jobs run on simulated data used the following input files (provided in `https://github.com/bergmanlab/mcclintock/blob/master/test/`): the UCSC sacCer2 version of the *S. cerevisiae* S288c reference genome (sacCer2.fasta, commit SHA 53a5e8f); reference TE annotations (reference_TE_locations.gff, commit SHA 571ef0e), taxonomy files (sac_cer_te_families.tsv, commit SHA 571ef0e), and canonical sequences for *S. cerevisiae* Ty elements (sac_cer_TE_seqs.fasta, commit SHA d649713) from [6]. Details of the four simulation experiments are as follows:

Simulation 1 aimed replicate previous results reported in the original McClintock paper [1] using predictions from McClintock 1 on WGS datasets created by the new simulation framework in McClintock 2. Simulation parameters were the same as in Nelson *et al.* [1] and are based on the well-established preferences of Ty elements to insert upstream of genes transcribed by RNA Pol III such as tRNA genes (reviewed in [7]). To create synthetic genomes, a single Ty element was sequentially chosen from the list of Ty families that are active in S288c (Ty1, Ty2, Ty3, and Ty4) and inserted upstream one of the 299 tRNA genes in reference genome. Potential insertion sites were set to be 195-200 bp upstream of tRNA genes for Ty1, Ty2 and Ty4, and 12-17 bp upstream of tRNA genes for Ty3. In total, 598 synthetic genomes were generated: 299 genomes each having one insertion upstream of one tRNA gene in the forward strand, and 299 genomes each having one insertion upstream one tRNA gene in the reverse strand. As in Nelson *et al.* [1], the wgsim read simulator (v1, build h7132678_5) [8] was then used to create WGS paired-end datasets with a fold-coverage of 100×, read lengths of 101 bp, median insert sizes of 300 bp and a base error rate of 1%. Performance for the six original components on simulated WGS datasets was evaluated using McClintock 1 (commit SHA ad0c6cb).

Simulation 2 aimed to cross-validate the accuracy of the McClintock re-implementation using predictions from McClintock 2 for the six original components available in McClintock 1. Construction of synthetic genomes and simulation of WGS read datasets was the same as in Simulation 1. Performance for the six original components on simulated WGS datasets was evaluated using predictions from McClintock 2 (commit SHA 7aa5298).

Simulation 3 aimed to evaluate the ability of all 12 components in McClintock 2 to detect TE insertions placed in biologically-realistic locations in the *S. cerevisiae* genome over a range of WGS fold-coverages. Simulation 3 used modified Ty targeting preferences and a different read simulator than in Simulations 1 and 2. First, rather than fixing the location of insertions for Ty1, Ty2, and Ty4 to be at one position (195-200 bp upstream of tRNA genes) as in Nelson *et al.* [1], we allowed synthetic insertions for these families to be placed in a broader region (50-800 bp upstream of tRNA genes) that better reflects patterns observed for both spontaneous and induced insertions [1, 9, 10, 11]. As above, potential insertion sites for Ty3 were set to be 12-17 bp upstream of tRNA genes as is observed for both spontaneous and induced insertions [1, 4, 12, 13]. Second, the tRNA gene and Ty family was chosen randomly from the list of tRNA genes and Ty families, rather than sequentially as in Nelson *et al.* [1]. Also, because of incompatibilities between the format of fastq sequences generated by wgsim [8] and one of the new components in McClintock 2 (TEbreak), we used the ART read simulator (v2016.06.05 build h874f42a_8) [14] for Simulation 3. Simulated WGS paired end datasets were generated by ART with a built-in error profile for the Illumina HiSeq 2500 instrument, read lengths of 101 bp, median insert sizes of 300 bp, and a standard deviation of simulated DNA fragment sizes of 10 bp (options: -ss HS25 -sam -p -s 10 -l 101 -m 300). For each synthetic genome, six sets of simulated reads were produced at different fold-coverages (3×, 6×, 12×, 25×, 50× and 100×). We also rounded the number of replicates per strand to 300 for each fold-coverage. Performance for all 12 components on simulated WGS datasets was evaluated using predictions from McClintock 2 (commit SHA 7aa5298).

Simulation 4 aimed to evaluate the performance of all 12 components in McClintock 2 to detect TE insertions in random, unique genomic regions over a range of WGS fold-coverages. Because Ty targeting of RNA Pol III transcribed regions has caused a historical accumulation of Ty fragments upstream of tRNA genes [5, 6], many synthetic non-reference Ty insertions in Simulations 1-3 are placed into fragments of pre-existing Ty sequences found in the reference genome. Simulation 4 therefore allows us to gain insight into component method performance for TE families that do not target repetitive DNA in the reference genome (unlike Ty elements in *S. cerevisiae*), and by contrasting with results from Simulation 3 to understand the effects that insertion into repetitive DNA has on TE detector performance in yeast. Unique regions were defined as the complement of regions annotated as Ty elements

in the UCSC sacCer2 version of the *S. cerevisiae* S288c reference genome [6]. Aside from differences in the location of potential insertion sites, construction of synthetic genomes and simulation of WGS read datasets was the same as in Simulation 3. Performance for all 12 components on simulated WGS datasets was evaluated using predictions from McClintock 2 (commit SHA 7aa5298).

Quantitative results from all four simulations can be found in Additional Files 2 and 3. Overlaps among component methods for true positive predictions at different window sizes and fold-coverages for Simulations 3 and 4 can be found in Additional File 4.

Cross-validation of the McClintock 2 meta-pipeline and simulation system

To validate the new McClintock implementation and simulation system, we used results from Simulations 1-3 (see above). To be able to compare with previous results, we used the same approach as in Nelson *et al.* [1] to summarize the six original component methods' performance across simulated samples. Namely, we calculated the average number of non-reference TE predictions made by each component overall and at different levels of positional accuracy determined by varying window sizes (within 0, 5, 100, 300 or 500 bp of the synthetic insertion). Because differences in component performance on the positive and negative strands were minimal (Additional File 2), here we simplified reporting of summary metrics relative to Nelson *et al.* [1] by averaging results from forward and reverse strands across simulations (Fig S1). The expected value of non-reference TE insertions per simulation is one, with values less than one representing a tendency for a TE detector to make false negative predictions and values greater than one representing a tendency for false positive predictions.

We initially asked whether we could replicate performance results for the original six McClintock components reported in Nelson *et al.* [1] using the new reproducible simulation system in McClintock 2 (Simulation 1). This simulation used identical TE insertion settings but a more recent version of McClintock 1 and different simulation implementation relative to Nelson *et al.* [1] (Table S1). As shown in Fig. S1, results from Simulation 1 generated very similar numbers (± 0.09) of average non-reference TE predictions as in Nelson *et al.* [1], with the exception of the total number of non-reference TE predictions made by RelocaTE being substantially lower ($\sim$25%) in the current study. This difference could arise from many sources (including different git commit SHAs for McClintock 1, component method dependencies, computing environments, simulation code, or run-to-run variation in component methods) and underscores the difficulty in fully reproducing previous TE detector benchmarking studies. Nevertheless, the general consistency between results reported previously by Nelson *et al.* [1] and those generated independently here (Simulation 1) indicates that our new Python-based reproducible simulation system yields similar results as the old Bash-based simulation system, and that performance estimates of the original six component methods generated by McClintock 1 are broadly replicable.

Next, we sought to test whether the new Python-based McClintock 2 meta-pipeline generates similar performance results as the original Bash-based McClintock system. Because of the differences observed between Nelson *et al.* [1] and Simulation 1 (Fig. S1), we chose to use the reproducible results for McClintock 1 generated here (Simulation 1) as a baseline to compare with the McClintock 2 meta-pipeline. Also, using Simulation 1 as a baseline fully controls for differences between the new Python-based simulation system and the Bash-based simulation system used in Nelson *et al.* [1], and therefore allows us to isolate any potential performance differences to the McClintock meta-pipeline itself. As shown in Fig. S1, no major differences in performance of the six original component methods (± 0.065) are observed for the Python-based (Simulation 2) and Bash-based (Simulation 1) implementations of the McClintock meta-pipeline. The largest difference observed is for RetroSeq, where this component exhibits improved performance in McClintock 2. Overall, this analysis demonstrates that performance estimates generated by the Python-based McClintock 2 are the same or better as those from the Bash-based McClintock 1 and indicates that no major differences should be experienced by users migrating from McClintock 1 to McClintock 2.

The approach used to simulate non-reference TE insertions in Nelson *et al.* [1] had a number of hard-coded constraints tailored for the yeast genome that made it unsuitable for application to other organismal contexts. Namely, the TE family and location of simulated insertions were generated sequentially from a candidate set of TE families and tRNA gene promoters, and the insertion positions were at fixed distances upstream of tRNA genes. To allow more general modeling of insertion preferences, we designed the new McClintock 2 simulation system to generate an instance of a randomly selected TE family to be randomly inserted into a set of arbitrary genomic locations specified in a configurable JSON file. By modifying parameters in the JSON file, the new simulation system is flexible enough to permit implementation of the Nelson *et al.* [1] approach as well as more biologically-realistic models of TE insertion, such as Ty insertion over a range of positions upstream of tRNA genes (see Materials and Methods for details). We tested if this new biologically-realistic insertion framework (Simulation 3) gave similar results at the same fold-coverage (100×) as the original sequential insertion framework with fixed Ty insertion locations (Simulation 2). As shown in Fig. S1, both the random (Simulation 3) and sequential (Simulation 2) insertion frameworks give

similar results (±0.17) with the exception of PoPoolationTE. We speculate that the unusually high number of total non-reference predictions made by PoPoolationTE in Simulation 3 may be caused by misclassification of reference TE insertions as non-reference TE insertions (see also [1]).

In summary, we can conclude that key improvements in McClintock 2 including (i) the Python-based implementation of the simulation system (Simulation 1 vs. Nelson *et al.* [1]), (ii) the Python-based implementation of the meta-pipeline (Simulation 2 vs. Simulation 1), and (ii) the random insertion model in the single insertion simulation framework (Simulation 3 vs. Simulation 2) together generate results that broadly replicate those published previously in Nelson *et al.* [1].

Supplementary Tables

Table S1: Distinguishing features of simulation experiments performed in Nelson *et al.* [1] and this study.

	Nelson *et al.* [1]	Simulation 1	Simulation 2	Simulation 3	Simulation 4
McClintock implementation	Bash	Bash	Python	Python	Python
McClintock commit SHA	e945d20	ad0c6cb	7aa5298	7aa5298	7aa5298
Simulation implementation	Bash	Python	Python	Python	Python
Simulation commit SHA	N.A.	7aa5298	7aa5298	7aa5298	7aa5298
TE insertion targets	tRNA promoter	tRNA promoter	tRNA promoter	tRNA promoter	Non-repetitive DNA
Insertion position	Fixed distance	Fixed distance	Fixed distance	Specified range	Specified range
Candidate selection	Serial	Serial	Serial	Random	Random
Read simulator	wgsim	wgsim	wgsim	ART	ART
Simulated fold-coverage	100×	100×	100×	3× to 100×	3× to 100×
Replicates per setting	299	299	299	300	300

Supplementary Figures

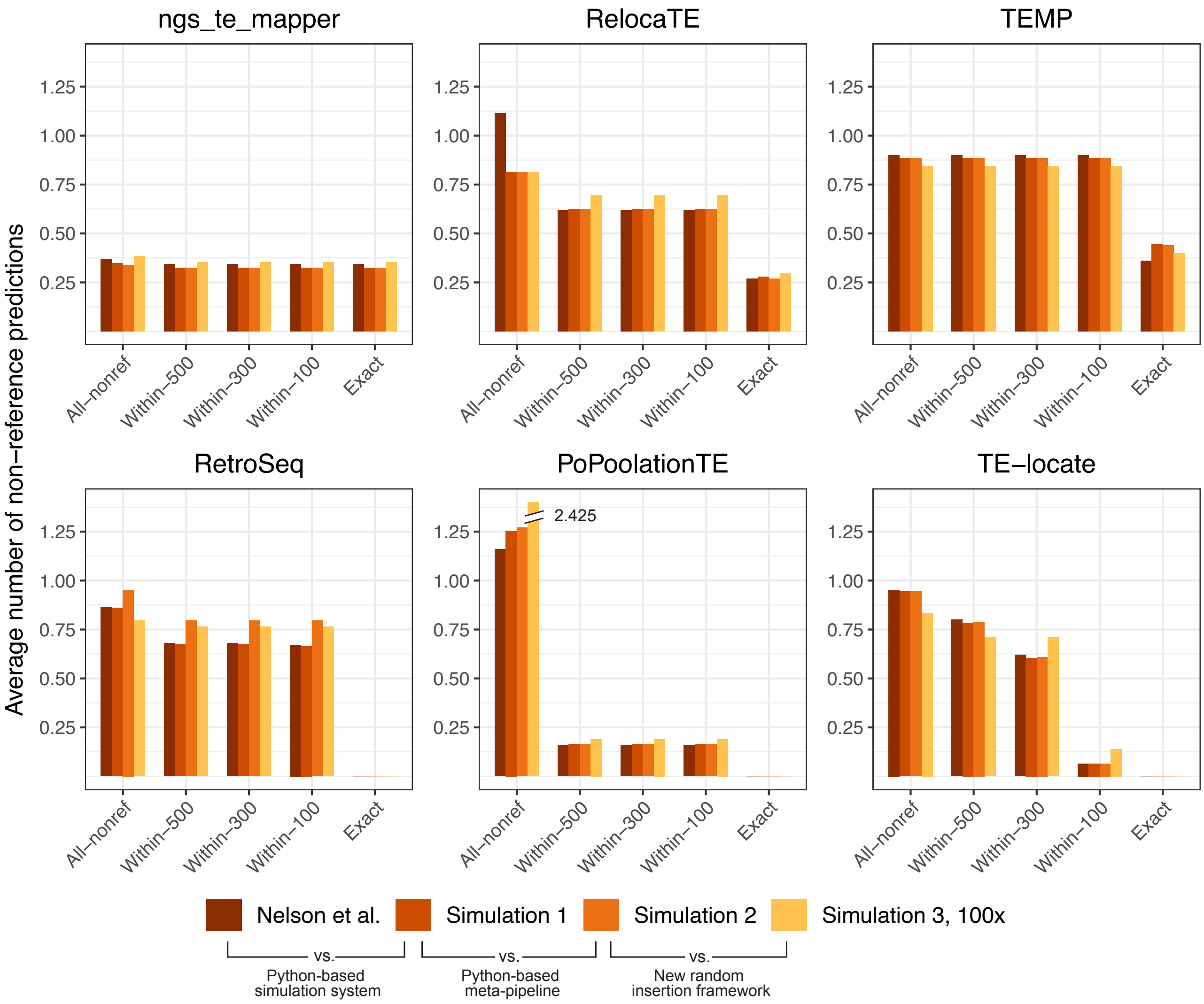

Figure S1: **Validation of McClintock 2 meta-pipeline and simulation system.** The bar plot shows the average number of all non-reference predictions and true positive predictions falling in different window sizes (exact, within 100 bp, within 300 bp and within 500 bp) across all simulated replicates, including old McClintock data [1], simulation 1, simulation 2 and simulation 3 (100×). Values shown in this figure are the average number of the forward and reverse strand since two strands were run separately in our simulation framework. Only six original methods included in McClintock are shown here for validation. The PoPoolationTE value exceeds y-axis limitation and thus is annotated with a break and the actual number.

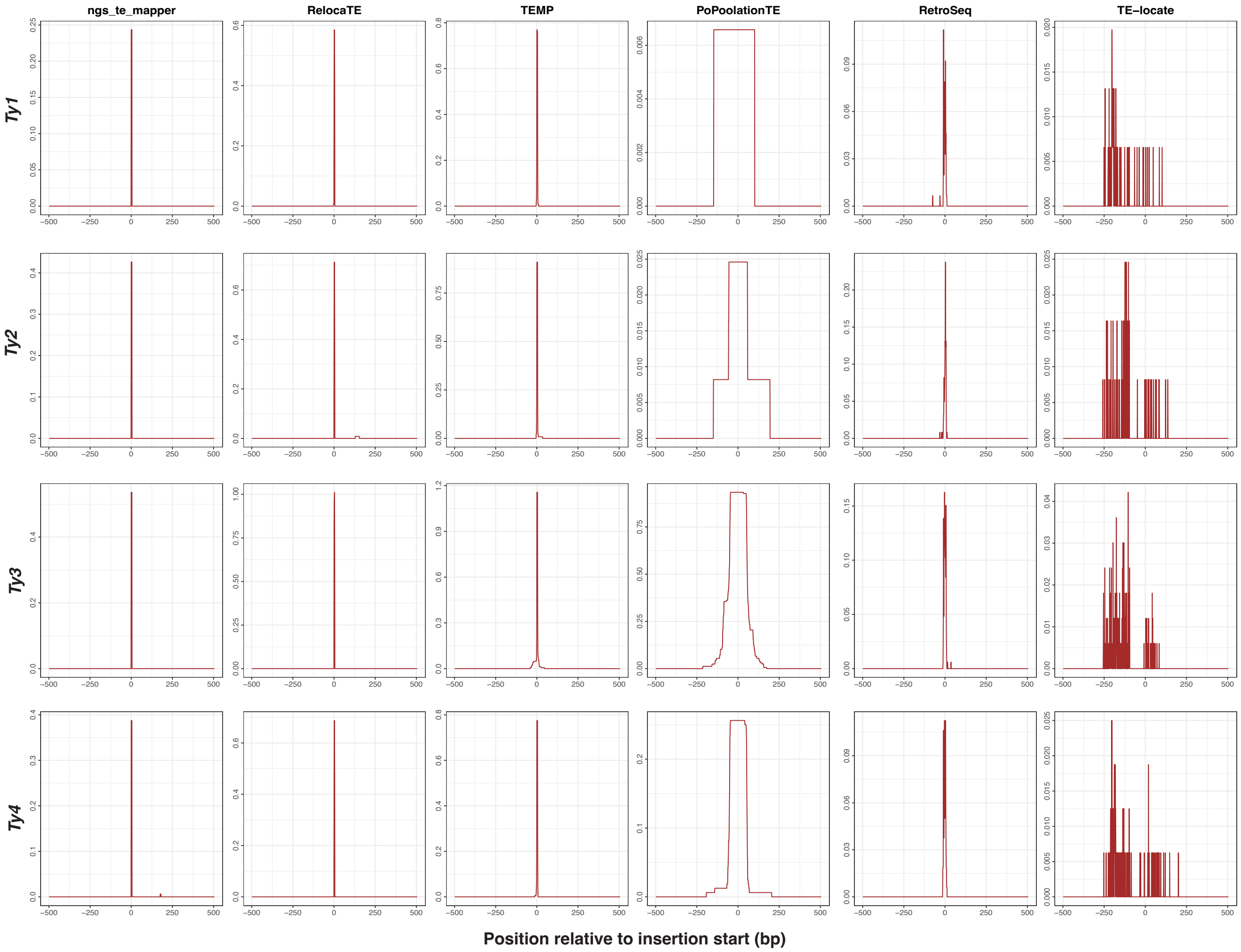

Figure S2: **Positional accuracy of six original McClintock component methods in simulated data generated by the yeast insertion model (Simulation 3).** Plots show coverage of all non-reference TE predictions relative to the coordinate of the start position of synthetic insertions in simulated datasets with 50× fold-coverage averaged across all 600 replicate simulations. Predictions more than 500 bp from the start position of synthetic insertions are not shown.

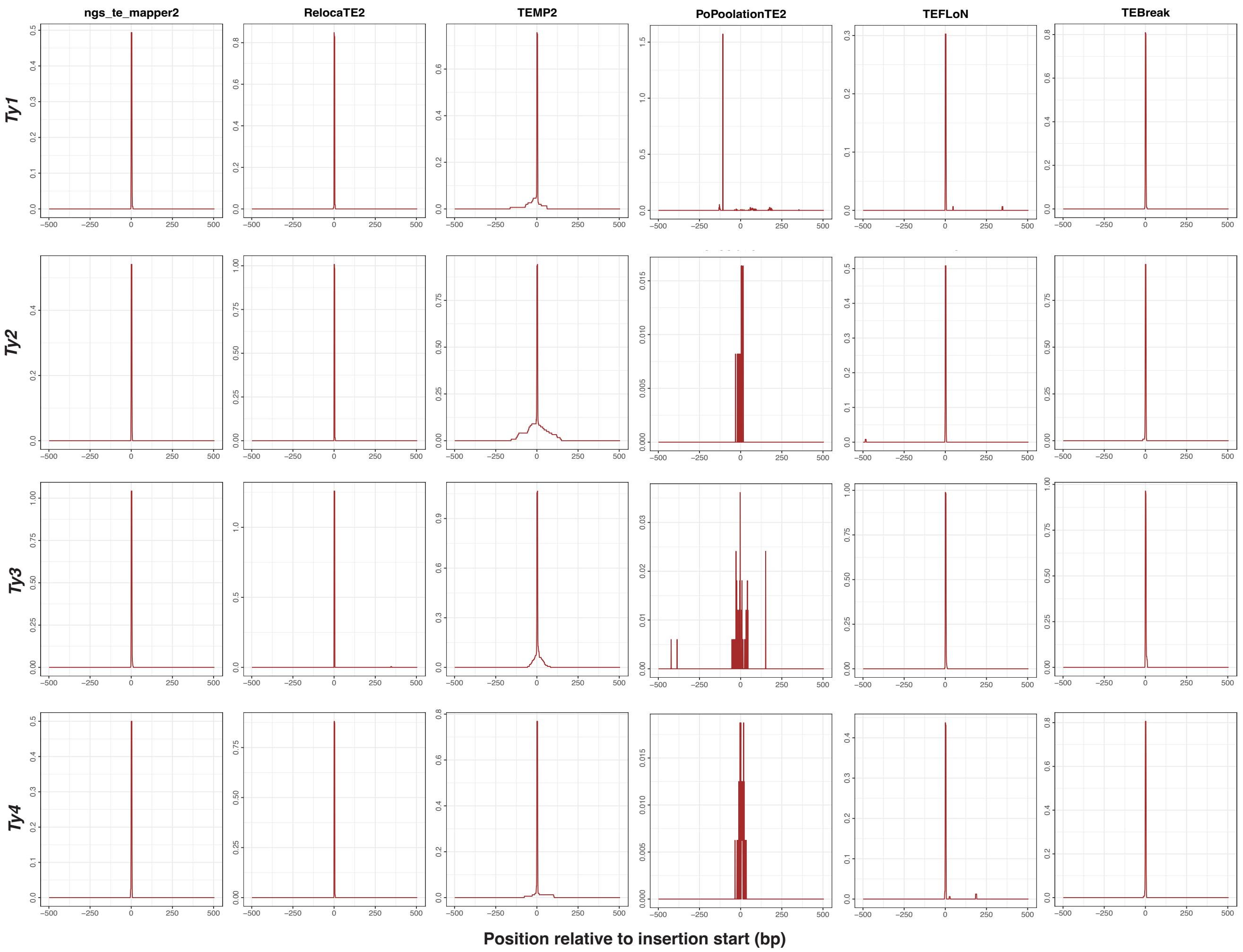

Figure S3: **Positional accuracy of six new McClintock component methods in simulated data generated by the yeast insertion model (Simulation 3).** Plotting criteria are the same as in Figure S2.

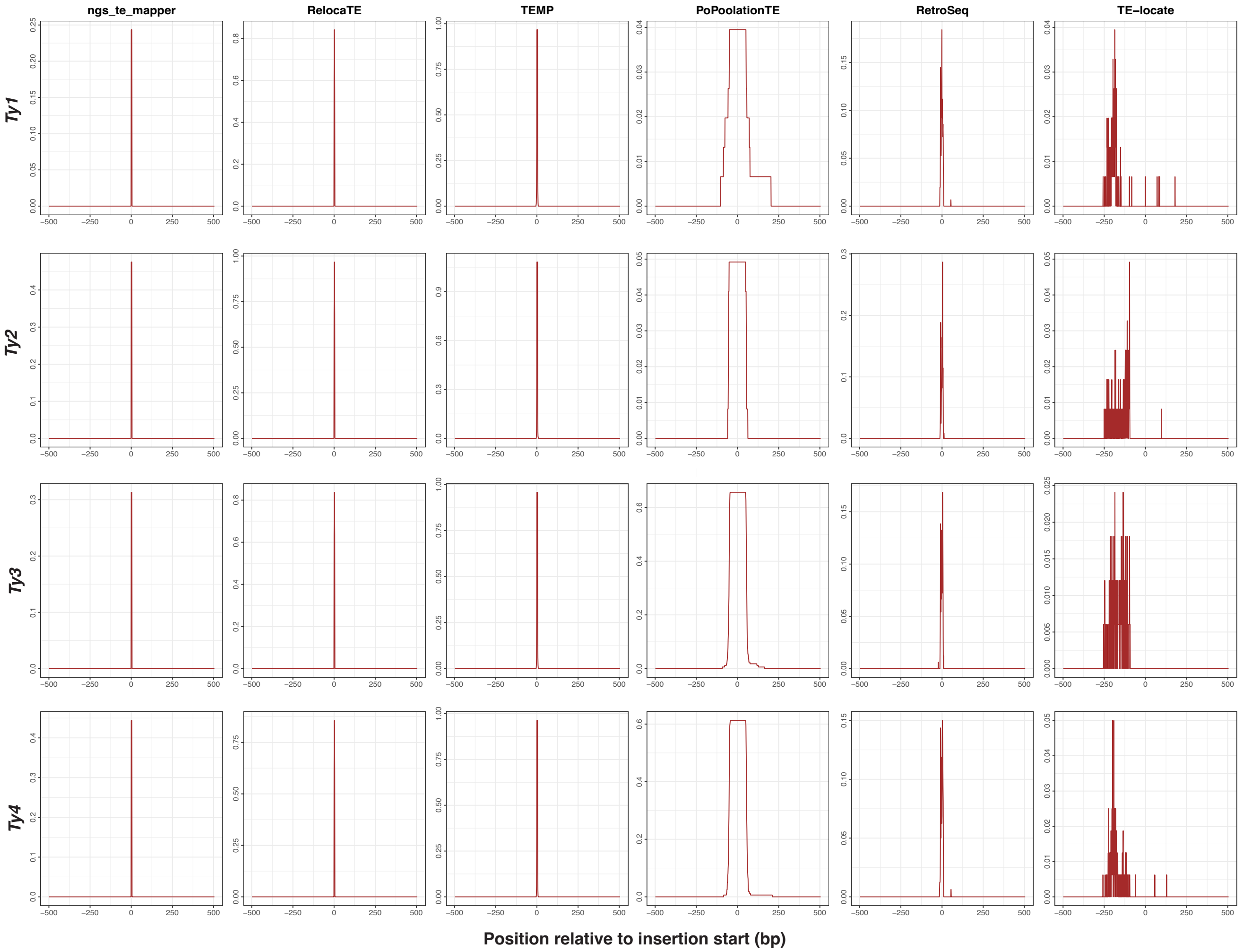

Figure S4: **Positional accuracy of six original McClintock component methods in simulated data generated by the random insertion model (Simulation 4).** Plotting criteria are the same as in Figure S2.

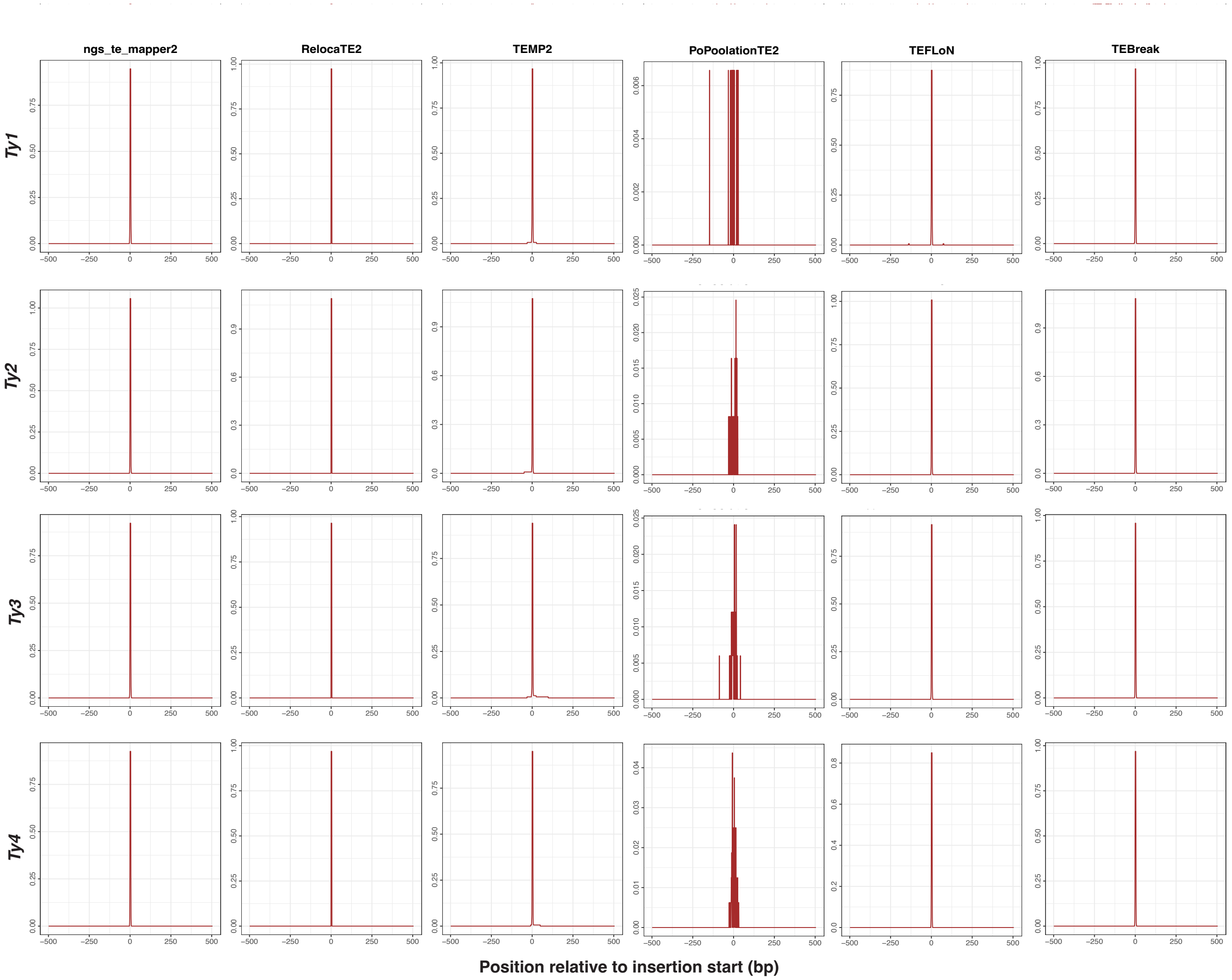

Figure S5: **Positional accuracy of six new McClintock component methods in simulated data generated by the random insertion model (Simulation 4).** Plotting criteria are the same as in Figure S2.

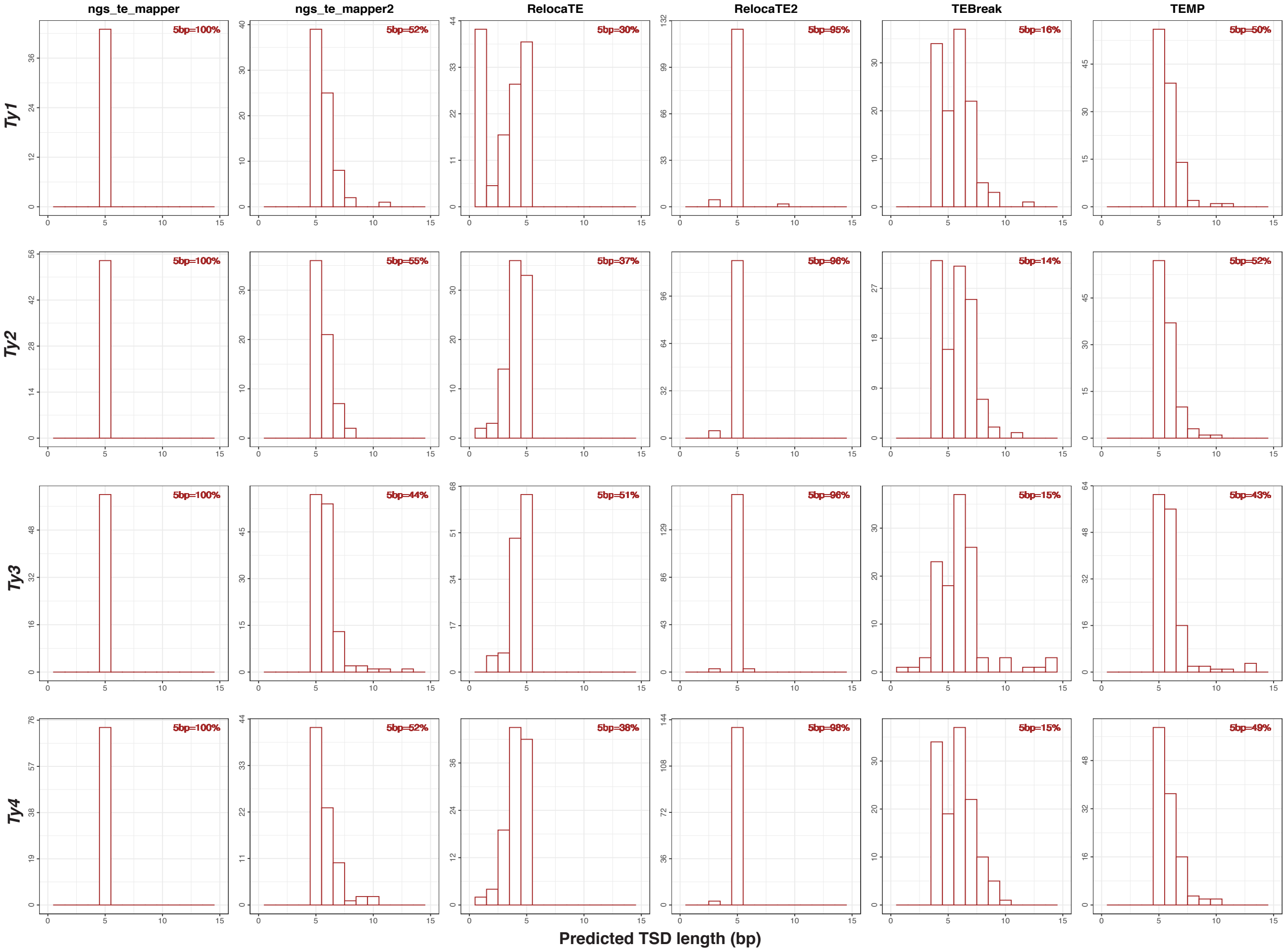

Figure S6: **TSD length distributions in simulated data generated by the yeast insertion model (Simulation 3).** Plots show histograms of TSD lengths for predictions in simulated datasets with 50× fold-coverage that have split-read evidence. Shown are all predictions made by ngs_te_mapper, ngs_te_mapper2, RelocaTE, RelocaTE2, and TEBreak and the subset of TEMP predictions with split-read evidence. Percentages indicate the proportion of predictions with the expected 5-bp TSD length.

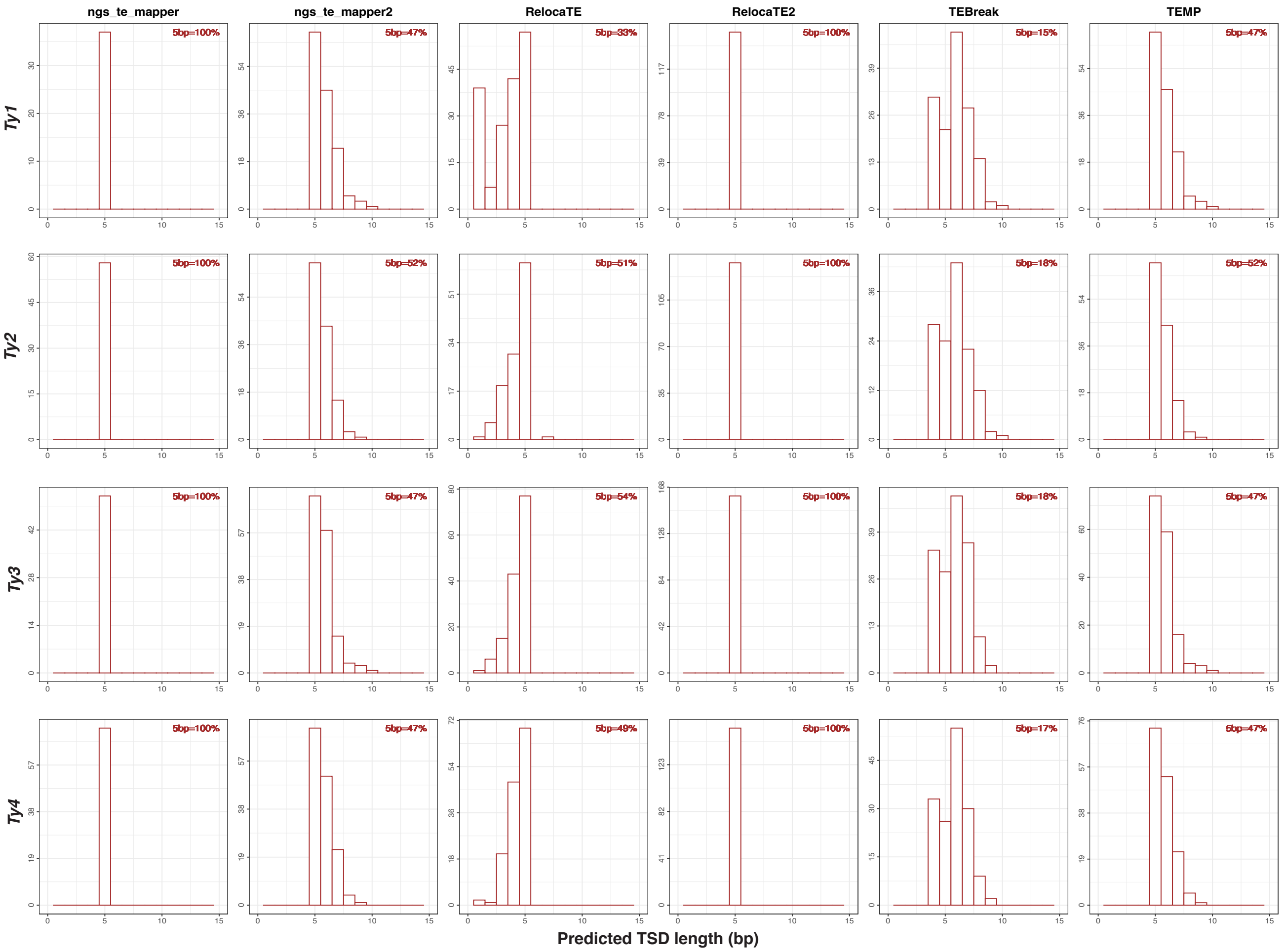

Figure S7: **TSD length distributions in simulated data generated by the random insertion model (Simulation 4).** Plotting criteria are the same as in Figure S6.

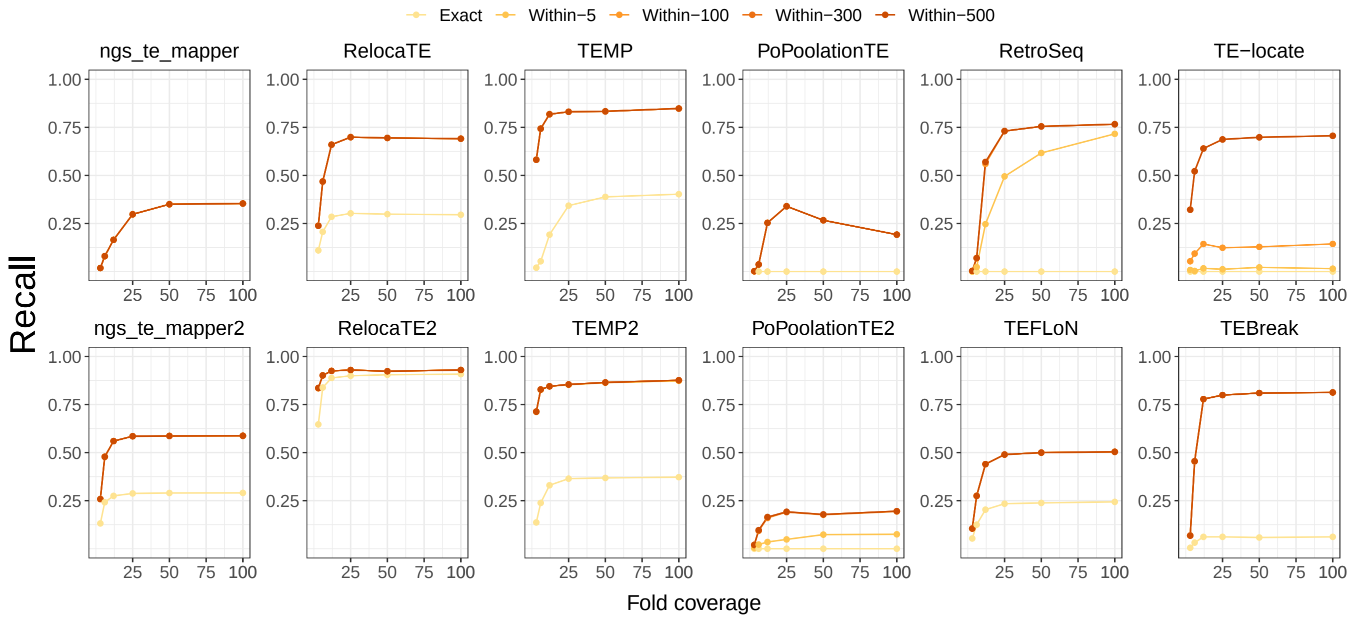

Figure S8: **Recall of McClintock 2 component methods in simulated data generated by the yeast insertion model (Simulation 3).** Shown are the recall of 12 component methods across variable fold-coverage (i.e., 3×, 6×, 12×, 25×, 50× and 100×). Curves in different colors indicate variable overlapping window sizes to identify true-positive predictions, including exact coordinates, within 5 bp, within 100 bp, within 300 bp, and within 500 bp. The six original McClintock component methods are shown on the first row and the six newly-integrated methods are shown on the second row.

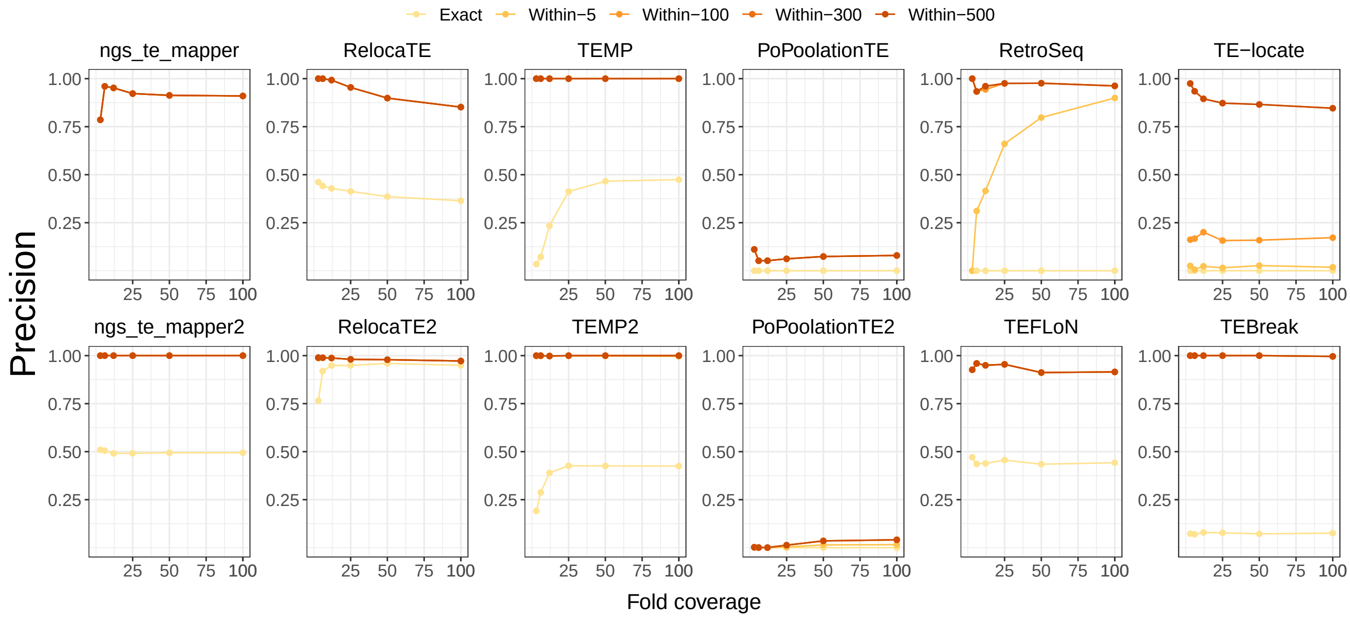

Figure S9: **Precision of McClintock 2 component methods in simulated data generated by the yeast insertion model (Simulation 3).** Plotting criteria are the same as in Figure S8.

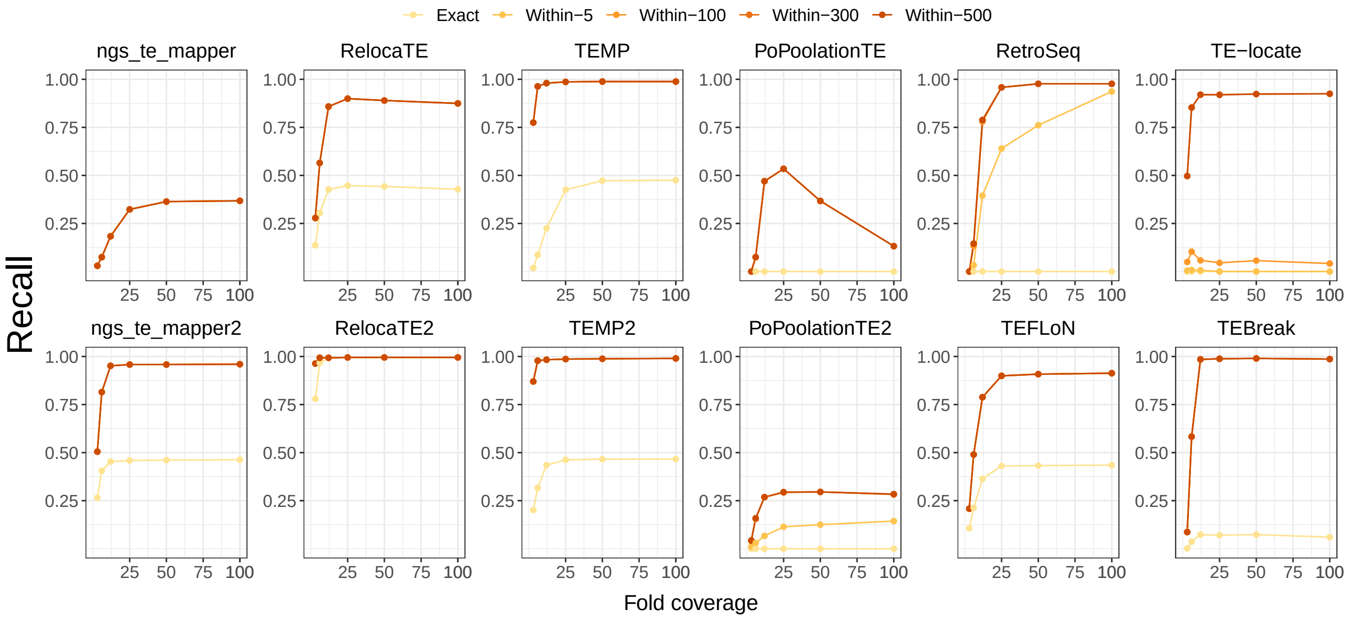

Figure S10: **Recall of McClintock 2 component methods in simulated data generated by the random insertion model (Simulation 4).** Plotting criteria are the same as in Figure S8.

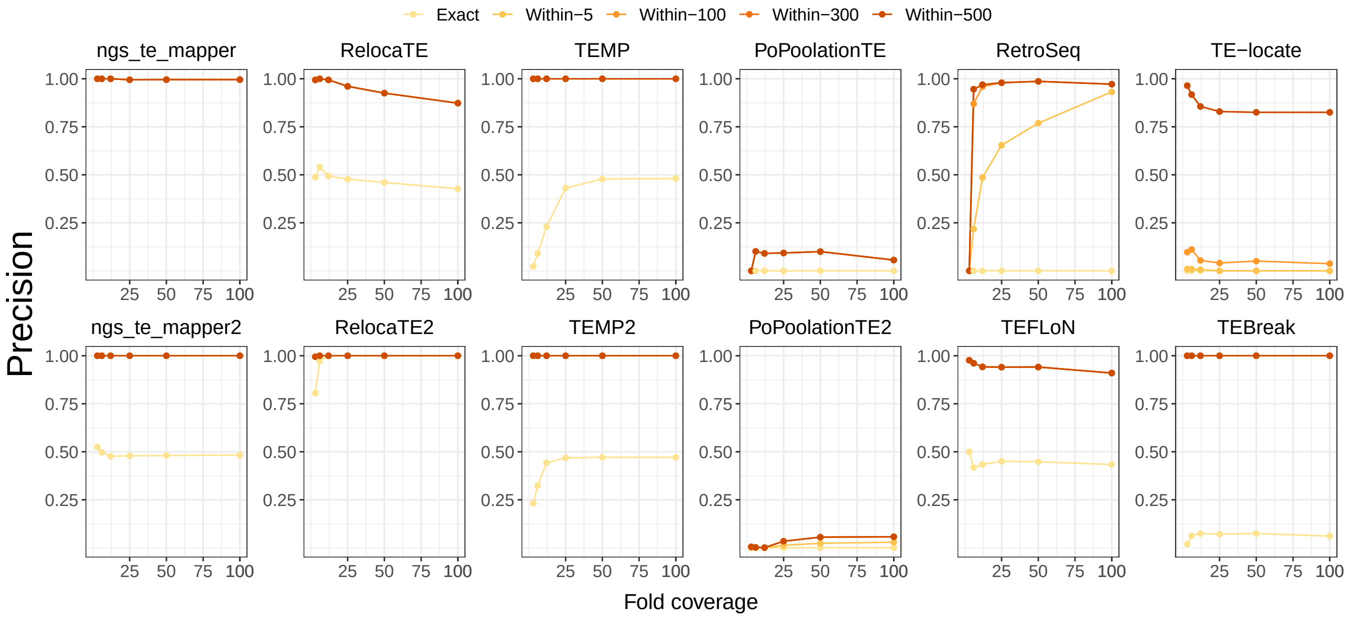

Figure S11: **Precision of McClintock 2 component methods in simulated data generated by the random insertion model (Simulation 4).** Plotting criteria are the same as in Figure S8.

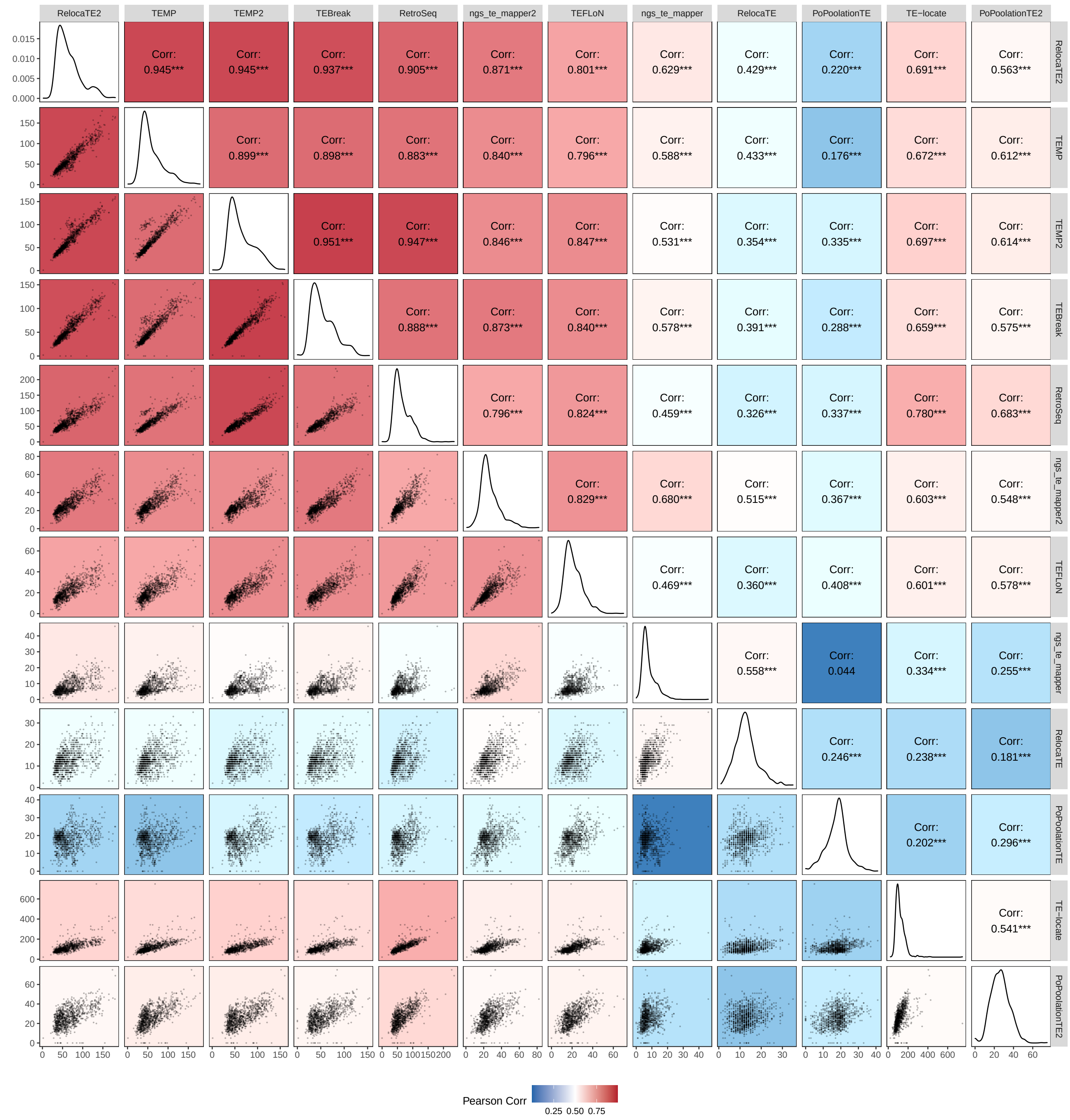

Figure S12: **Correlation across 12 component methods in all non-reference predictions for 1,011 yeast isolates.** The number of non-reference predictions for each yeast sample was plotted and compared across 12 component methods. WGS data are from [15, 16] and down-sampled to 50× fold-coverage. The panels in lower-left triangle are scatter plots on total TE count of each isolate between the corresponding pairs of methods. Panels in the upper-right triangle reveal the Pearson correlation between pairs of methods. Background colors for each panel are selected related to the Pearson correlation statistic, where higher and lower correlations are plotted in red and blue, respectively. Diagonal panels show the distribution of TE counts made by each component method. The component methods are ordered according to the general performance revealed in our simulation results.

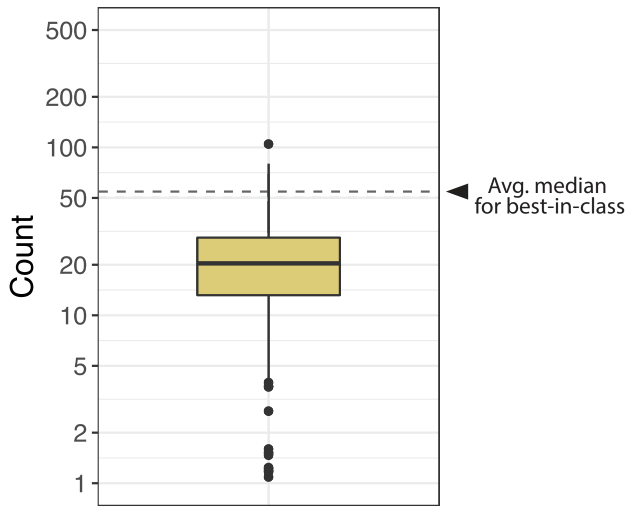

Figure S13: **Distribution of estimated copy number of full-length TEs across 1,011 yeast isolates.** Shown is the distribution of copy number of full-length TEs per strain (summed over all Ty families) estimated by the McClintock 2 coverage module using internal coding regions of all Ty families. Estimated full-length TE copy number includes both reference and non-reference TEs, but does not include solo LTRs. All WGS samples from [15, 16] were down-sampled to 50× fold-coverage. The y-axis is transferred to a $\log_{10}$ scale. The line inside the box indicates the median value, the colored box shows the interquartile range (IQR), whiskers show values 1.5×IQR of the upper or lower quartiles, and the dots indicate outliers that beyond 1.5×IQR. The dashed line indicates the average of median numbers non-reference Ty insertions per strain predicted by the four "best-in-class" component methods (RelocaTE2, TEMP, TEMP2 and TEBreak) (see Main Text for details).

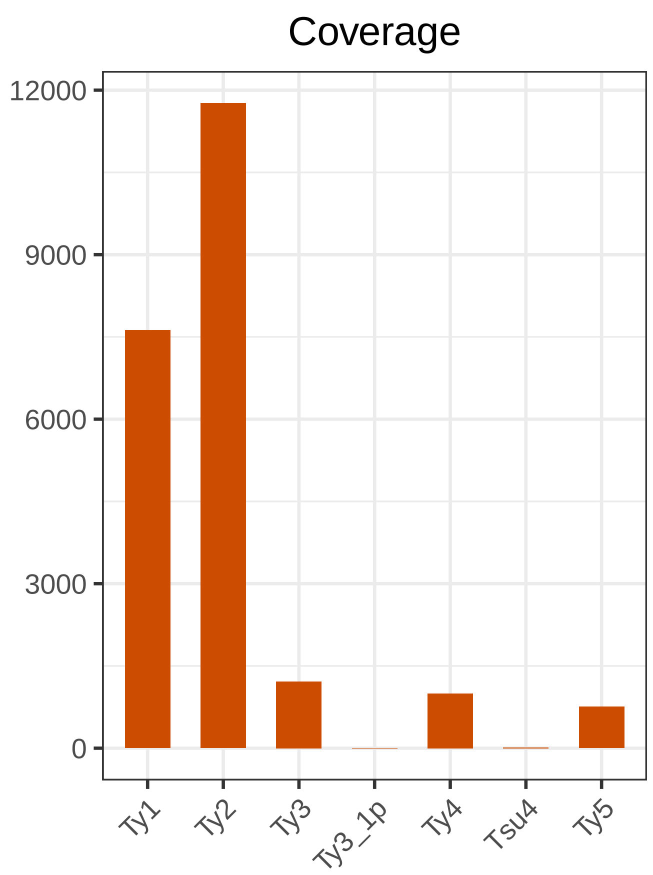

Figure S14: **Estimated total number of full-length TEs per Ty family across 1,011 *S. cerevisiae* isolates.** Shown is the total copy number of full-length TEs (summed across all strains) estimated by the McClintock 2 coverage module using internal coding regions of all Ty families. Estimated full-length TE copy number includes both reference and non-reference TEs, but does not include solo LTRs. All WGS samples from [15, 16] were down-sampled to 50× fold-coverage.

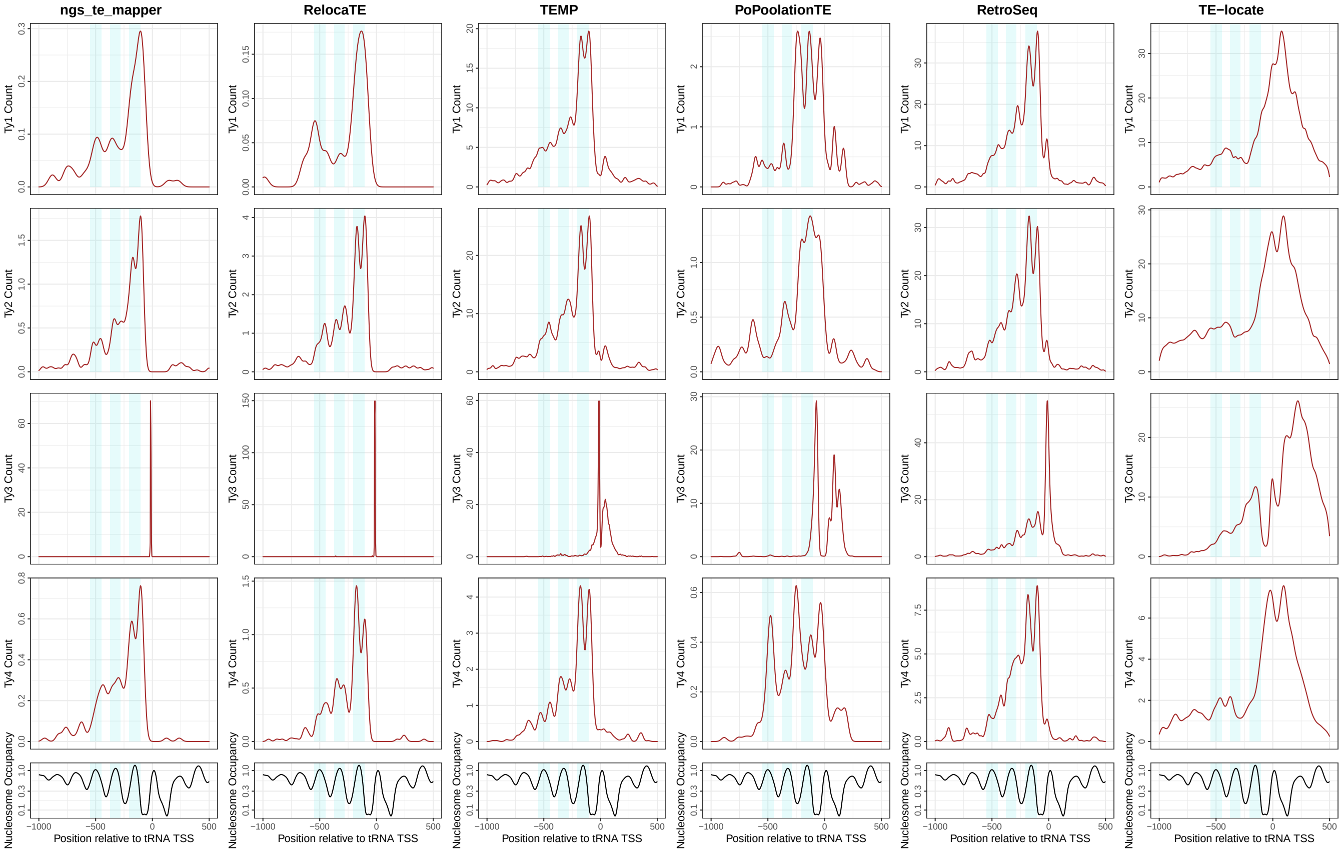

Figure S15: **Predicted TE insertion profiles around tRNA genes in *S. cerevisiae* for the six original McClintock component methods.** The first four rows show non-reference TE predictions made by six original methods in McClintock [1] from 1000 bp upstream and 500 bp downstream of tRNA genes partitioned by Ty family (summed over all strains) in 1,011 *S. cerevisiae* WGS samples [15, 16], down-sampled to 50× fold-coverage. Only the four active Ty families are included in this analysis. The bottom row shows nucleosome occupancy inferred using MNase-seq data from [17]. Light blue shading indicates 100-bp regions surrounding peaks of nucleosome occupancy.

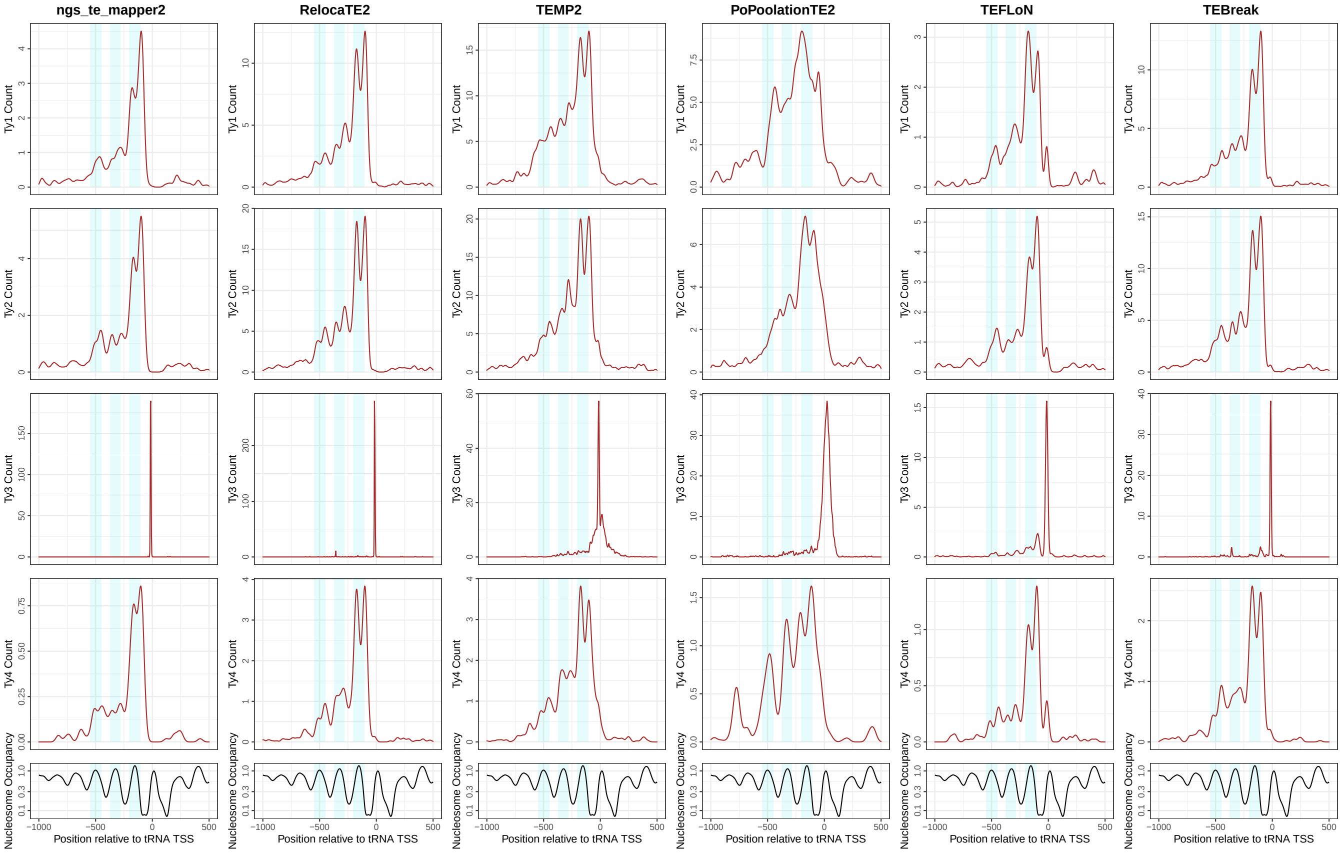

Figure S16: **Predicted TE insertion profiles around tRNA genes in *S. cerevisiae* for the six new McClintock component methods.** Plotting criteria are the same as in Figure S15.

Supplemental References

[1] M. G. Nelson, R. S. Linheiro, and C. M. Bergman, "McClintock: an integrated pipeline for detecting transposable element insertions in whole-genome shotgun sequencing data," *G3*, vol. 7, pp. 2749–2762, Aug. 2017.

[2] J. Gafner and P. Philippsen, "The yeast transposon Ty1 generates duplications of target DNA on insertion," *Nature*, vol. 286, pp. 414–418, July 1980.

[3] L. A. Rinckel and D. J. Garfinkel, "Influences of histone stoichiometry on the target site preference of retrotransposons Ty1 and Ty2 in Saccharomyces cerevisiae," *Genetics*, vol. 142, pp. 761–776, Mar. 1996.

[4] D. L. Chalker and S. B. Sandmeyer, "Transfer RNA genes are genomic targets for de novo transposition of the yeast retrotransposon Ty3," *Genetics*, vol. 126, pp. 837–850, Dec. 1990.

[5] J. M. Kim, S. Vanguri, J. D. Boeke, A. Gabriel, and D. F. Voytas, "Transposable elements and genome organization: a comprehensive survey of retrotransposons revealed by the complete Saccharomyces cerevisiae genome sequence," *Genome Res*, vol. 8, pp. 464–478, May 1998.

[6] M. Carr, D. Bensasson, and C. M. Bergman, "Evolutionary genomics of transposable elements in Saccharomyces cerevisiae," *PLOS ONE*, vol. 7, p. e50978, Nov. 2012.

[7] A. Bonnet and P. Lesage, "Light and shadow on the mechanisms of integration site selection in yeast Ty retrotransposon families," *Curr Genet*, vol. 67, pp. 347–357, June 2021.

[8] H. Li, "wgsim." https://github.com/lh3/wgsim.

[9] H. Ji, D. P. Moore, M. A. Blomberg, L. T. Braiterman, D. F. Voytas, G. Natsoulis, and J. D. Boeke, "Hotspots for unselected Ty1 transposition events on yeast chromosome III are near tRNA genes and LTR sequences," *Cell*, vol. 73, pp. 1007–1018, June 1993.

[10] J. A. Baller, J. Gao, R. Stamenova, M. J. Curcio, and D. F. Voytas, "A nucleosomal surface defines an integration hotspot for the Saccharomyces cerevisiae Ty1 retrotransposon," *Genome Res*, vol. 22, pp. 704–713, Apr. 2012.

[11] L. Mularoni, Y. Zhou, T. Bowen, S. Gangadharan, S. J. Wheelan, and J. D. Boeke, "Retrotransposon Ty1 integration targets specifically positioned asymmetric nucleosomal DNA segments in tRNA hotspots," *Genome Res*, vol. 22, pp. 693–703, Apr. 2012.

[12] D. L. Chalker and S. B. Sandmeyer, "Ty3 integrates within the region of RNA polymerase III transcription initiation," *Genes Dev*, vol. 6, pp. 117–128, Jan. 1992.

[13] X. Qi, K. Daily, K. Nguyen, H. Wang, D. Mayhew, P. Rigor, S. Forouzan, M. Johnston, R. D. Mitra, P. Baldi, and S. Sandmeyer, "Retrotransposon profiling of RNA polymerase III initiation sites," *Genome Res*, vol. 22, pp. 681–92, Apr. 2012.

[14] W. Huang, L. Li, J. R. Myers, and G. T. Marth, "ART: a next-generation sequencing read simulator," *Bioinformatics*, vol. 28, pp. 593–594, Feb. 2012.

[15] P. K. Strope, D. A. Skelly, S. G. Kozmin, G. Mahadevan, E. A. Stone, P. M. Magwene, F. S. Dietrich, and J. H. McCusker, "The 100-genomes strains, an S. cerevisiae resource that illuminates its natural phenotypic and genotypic variation and emergence as an opportunistic pathogen," *Genome Res*, vol. 25, pp. 762–774, May 2015.

[16] J. Peter, M. D. Chiara, A. Friedrich, J.-X. Yue, D. Pflieger, A. Bergstrom, A. Sigwalt, B. Barre, K. Freel, A. Llored, C. Cruaud, K. Labadie, J.-M. Aury, B. Istace, K. Lebrigand, P. Barbry, S. Engelen, A. Lemainque, P. Wincker, G. Liti, and J. Schacherer, "Genome evolution across 1,011 Saccharomyces cerevisiae isolates," *Nature*, vol. 556, pp. 339–344, Apr. 2018.

[17] E. Oberbeckmann, M. Wolff, N. Krietenstein, M. Heron, J. L. Ellins, A. Schmid, S. Krebs, H. Blum, U. Gerland, and P. Korber, "Absolute nucleosome occupancy map for the Saccharomyces cerevisiae genome," *Genome Res*, vol. 29, pp. 1996–2009, Dec. 2019.
