# Supplementary material for: Reproducible evaluation of transposable element detectors with McClintock 2 guides accurate inference of Ty insertion patterns in yeast
Source: Mob DNA. 2023 Jul 14;14:8. doi: 10.1186/s13100-023-00296-4 (PMC10347736; doi:10.1186/s13100-023-00296-4)
Supplement: Supplementary file 4 — Additional file 4. Overlaps between numbers of non-reference TEs predicted by McClintock component methods in simulated data. UpSet plots visualizing overlaps among component methods for true positive predictions at different window sizes and fold-coverages for Simulations 3 and 4. [file 13100_2023_296_MOESM4_ESM.zip › intersection/sim4_upsetplot_100x_0.pdf]

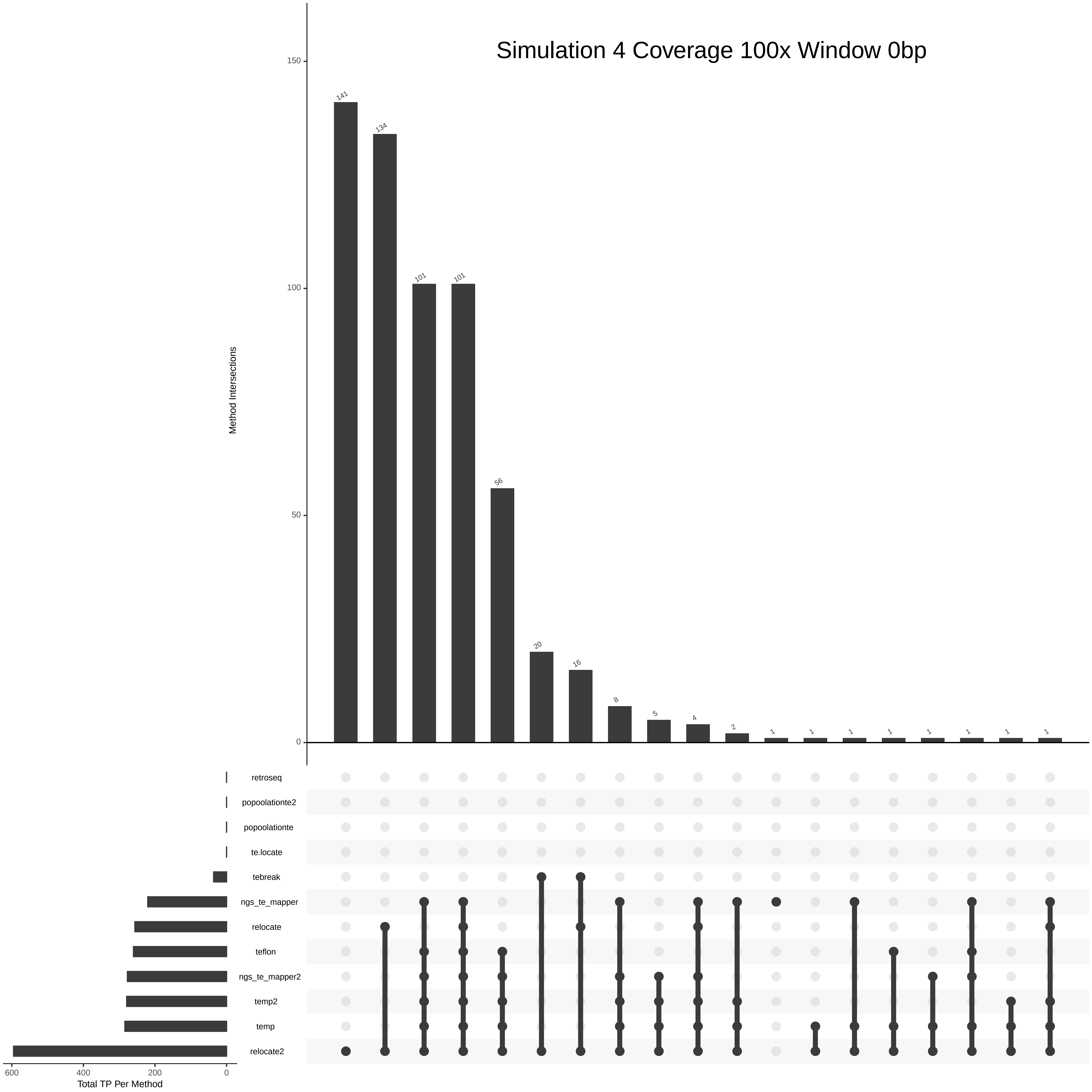

Simulation 4 Coverage 100x Window 0bp
Method Intersections
150
100
50
0
141
134
101
101
56
20
16
8
5
4
2
1
1
1
1
1
1
1
1
retroseq
popoolationte2
popoolationte
te.locate
tebreak
ngs_te_mapper
relocate
teflon
ngs_te_mapper2
temp2
temp
relocate2
600
400
200
0
Total TP Per Method
