# Supplementary material for: Reproducible evaluation of transposable element detectors with McClintock 2 guides accurate inference of Ty insertion patterns in yeast
Source: Mob DNA. 2023 Jul 14;14:8. doi: 10.1186/s13100-023-00296-4 (PMC10347736; doi:10.1186/s13100-023-00296-4)
Supplement: Supplementary file 4 — Additional file 4. Overlaps between numbers of non-reference TEs predicted by McClintock component methods in simulated data. UpSet plots visualizing overlaps among component methods for true positive predictions at different window sizes and fold-coverages for Simulations 3 and 4. [file 13100_2023_296_MOESM4_ESM.zip › intersection/sim4_upsetplot_6x_300.pdf]

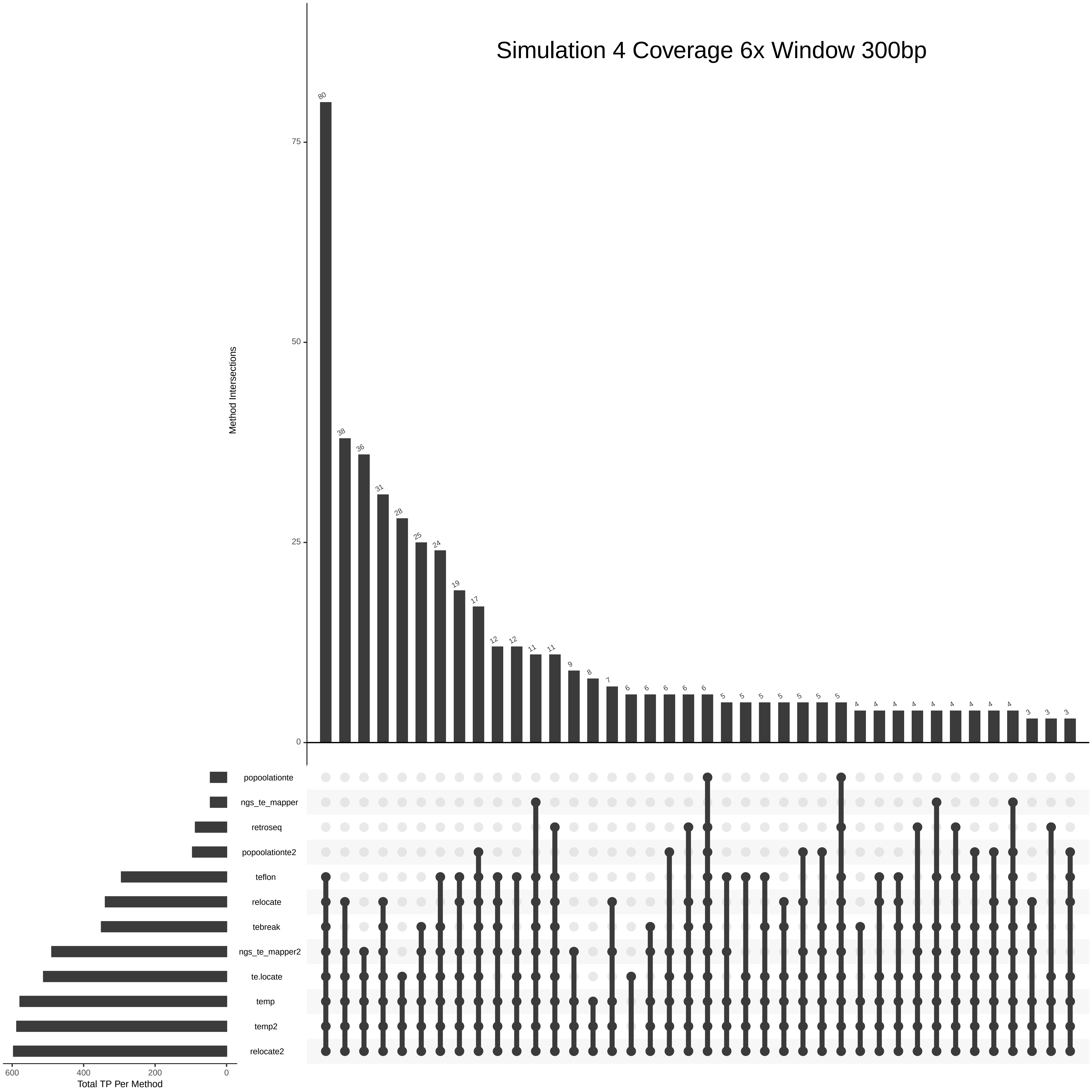

Simulation 4 Coverage 6x Window 300bp
Method Intersections
80
38
36
31
28
25
24
19
17
12
12
11
11
9
8
7
6
6
6
6
6
5
5
5
5
5
5
5
4
4
4
4
4
4
4
4
4
3
3
3
0
25
50
75
popoolationte
ngs_te_mapper
retroseq
popoolationte2
teflon
relocate
tebreak
ngs_te_mapper2
te.locate
temp
temp2
relocate2
600
400
200
0
Total TP Per Method
