# Supplementary material for: Reproducible evaluation of transposable element detectors with McClintock 2 guides accurate inference of Ty insertion patterns in yeast
Source: Mob DNA. 2023 Jul 14;14:8. doi: 10.1186/s13100-023-00296-4 (PMC10347736; doi:10.1186/s13100-023-00296-4)
Supplement: Supplementary file 4 — Additional file 4. Overlaps between numbers of non-reference TEs predicted by McClintock component methods in simulated data. UpSet plots visualizing overlaps among component methods for true positive predictions at different window sizes and fold-coverages for Simulations 3 and 4. [file 13100_2023_296_MOESM4_ESM.zip › intersection/sim3_upsetplot_100x_100.pdf]

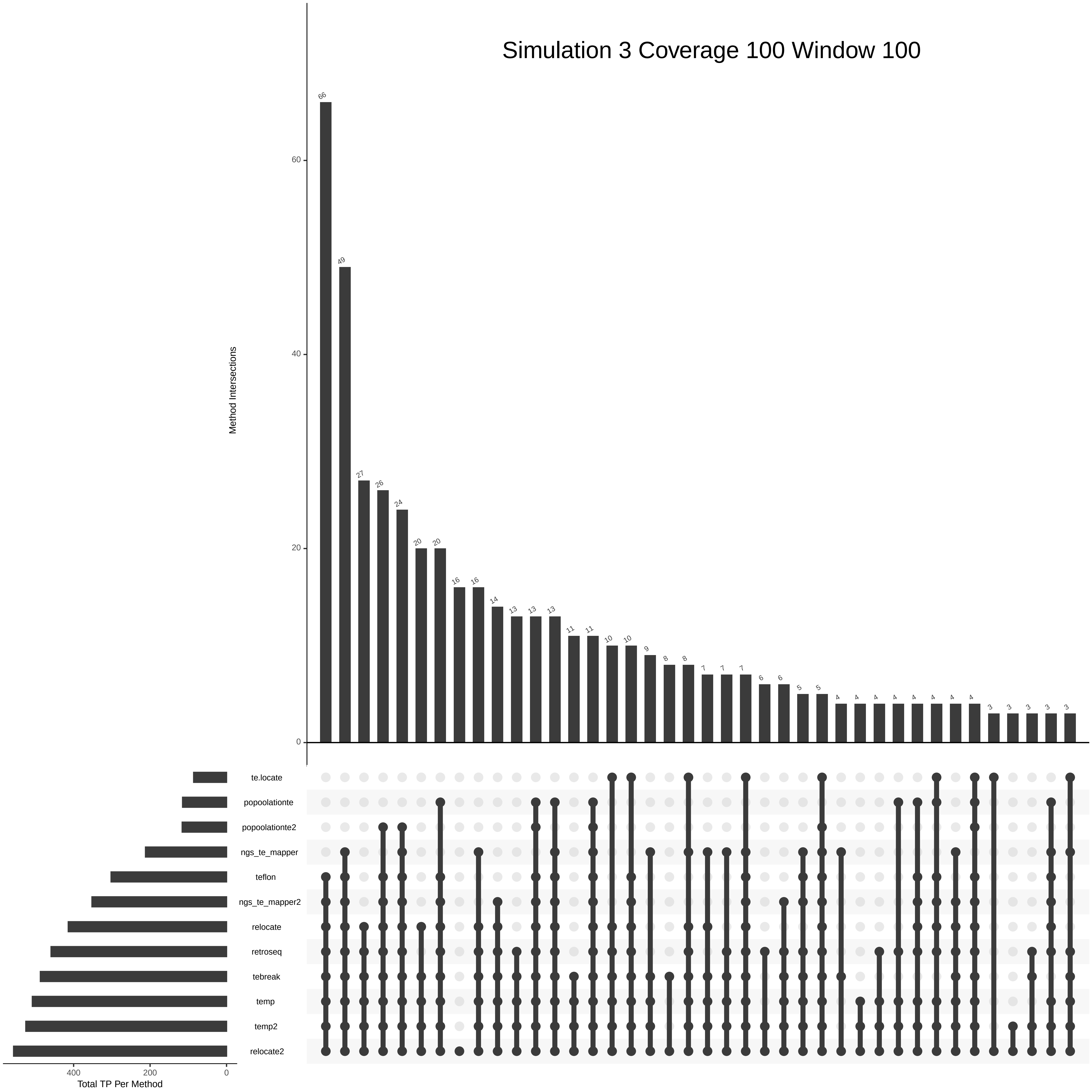

Simulation 3 Coverage 100 Window 100
Method Intersections
66
49
27
26
24
20
20
16
16
14
13
13
13
11
11
10
10
9
8
8
7
7
7
6
6
5
5
4
4
4
4
4
4
4
4
3
3
3
3
3
0
20
40
60
te.locate
popoolationte
popoolationte2
ngs_te_mapper
teflon
ngs_te_mapper2
relocate
retroseq
tebreak
temp
temp2
relocate2
400
200
0
Total TP Per Method
