# Supplementary material for: Reproducible evaluation of transposable element detectors with McClintock 2 guides accurate inference of Ty insertion patterns in yeast
Source: Mob DNA. 2023 Jul 14;14:8. doi: 10.1186/s13100-023-00296-4 (PMC10347736; doi:10.1186/s13100-023-00296-4)
Supplement: Supplementary file 4 — Additional file 4. Overlaps between numbers of non-reference TEs predicted by McClintock component methods in simulated data. UpSet plots visualizing overlaps among component methods for true positive predictions at different window sizes and fold-coverages for Simulations 3 and 4. [file 13100_2023_296_MOESM4_ESM.zip › intersection/sim3_upsetplot_50x_5.pdf]

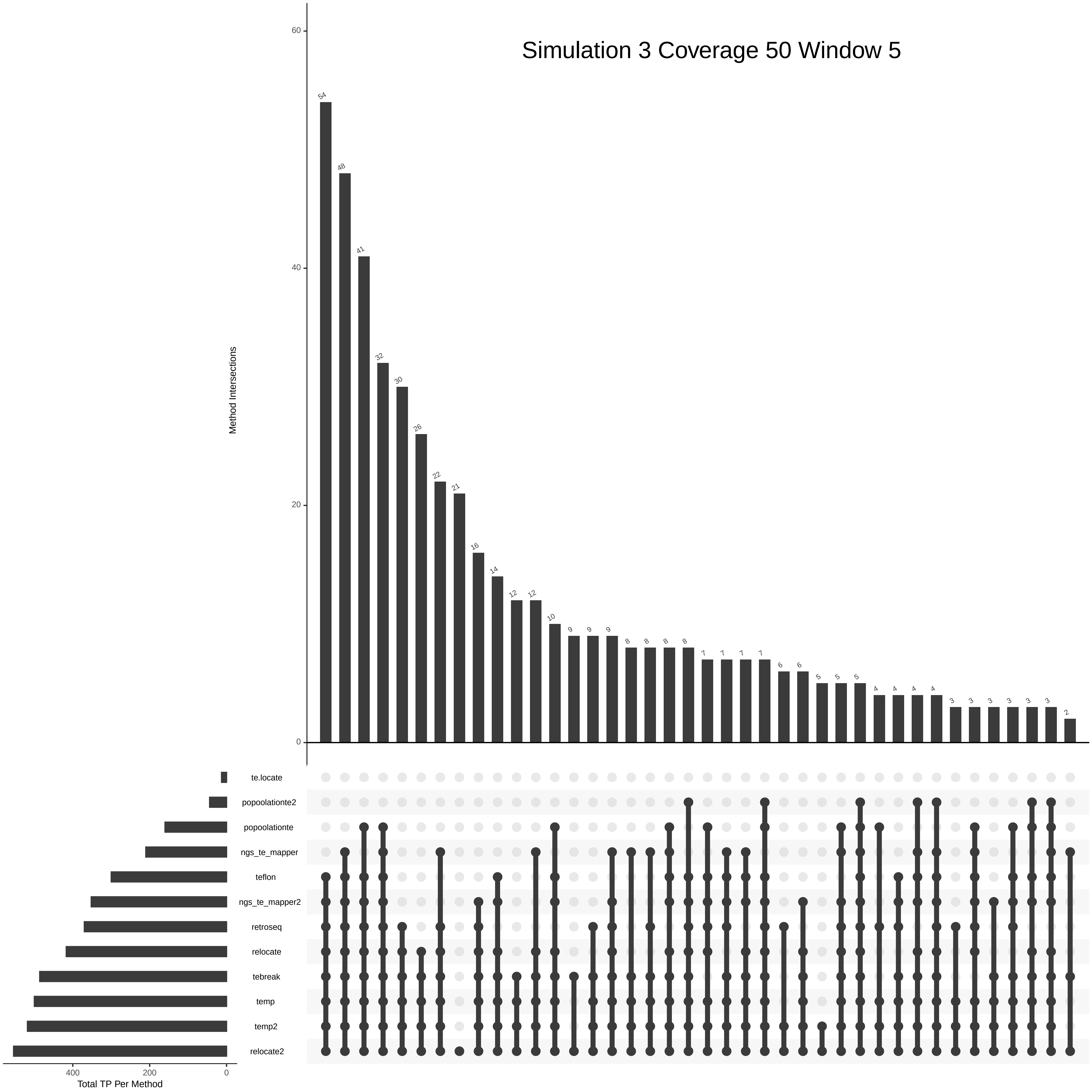

Simulation 3 Coverage 50 Window 5
Method Intersections
0
20
40
60
54
48
41
32
30
26
22
21
16
14
12
12
10
9
9
9
8
8
8
8
7
7
7
7
6
6
5
5
5
4
4
4
4
3
3
3
3
3
3
2
te.locate
popoolationte2
popoolationte
ngs_te_mapper
teflon
ngs_te_mapper2
retroseq
relocate
tebreak
temp
temp2
relocate2
Total TP Per Method
400
200
0
