# Supplementary figures and images for: Reproducible evaluation of transposable element detectors with McClintock 2 guides accurate inference of Ty insertion patterns in yeast
Source: Mob DNA. 2023 Jul 14;14:8. doi: 10.1186/s13100-023-00296-4 (PMC10347736; doi:10.1186/s13100-023-00296-4)

Simulation 3 Coverage 50 Window 100

Method Intersections

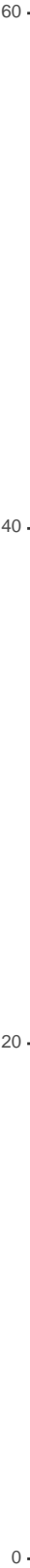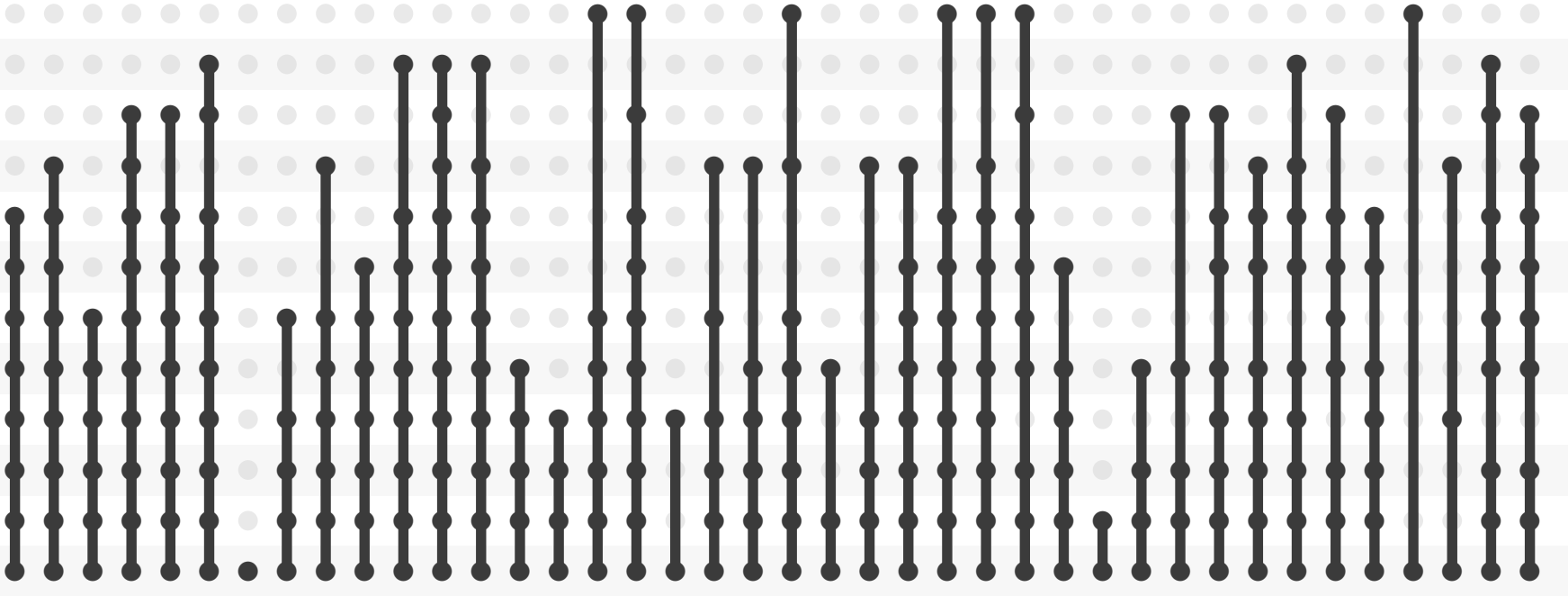

Total TP Per Method

Supplement: Supplementary file 4 — Additional file 4. Overlaps between numbers of non-reference TEs predicted by McClintock component methods in simulated data. UpSet plots visualizing overlaps among component methods for true positive predictions at different window sizes and fold-coverages for Simulations 3 and 4. [file 13100_2023_296_MOESM4_ESM.zip › intersection/sim3_upsetplot_50x_100.pdf]

Simulation 3 Coverage 3 Window 500

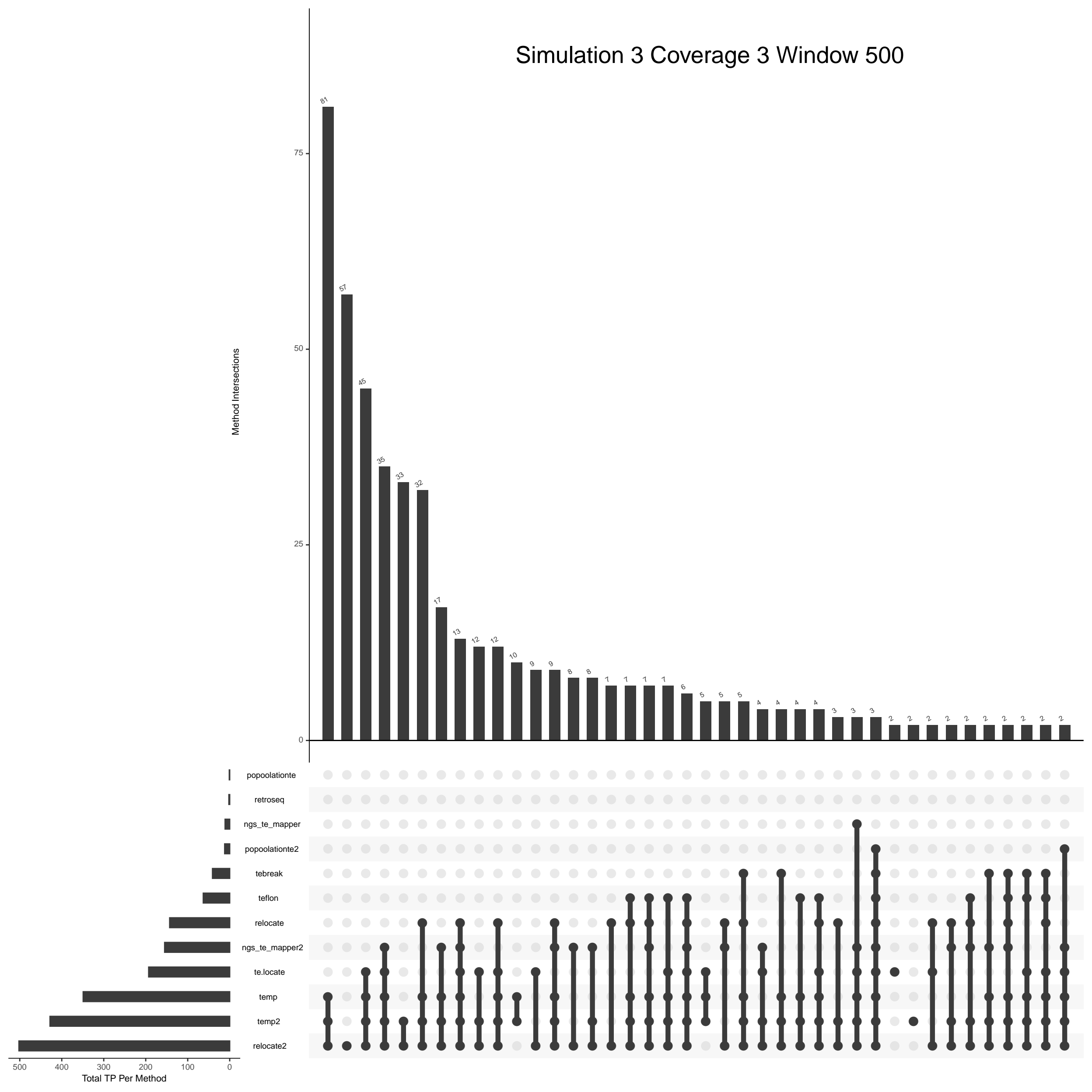

Supplement: Supplementary file 4 — Additional file 4. Overlaps between numbers of non-reference TEs predicted by McClintock component methods in simulated data. UpSet plots visualizing overlaps among component methods for true positive predictions at different window sizes and fold-coverages for Simulations 3 and 4. [file 13100_2023_296_MOESM4_ESM.zip › intersection/sim3_upsetplot_3x_500.pdf]

Simulation 3 Coverage 50 Window 300

Method Intersections

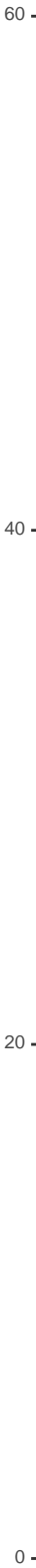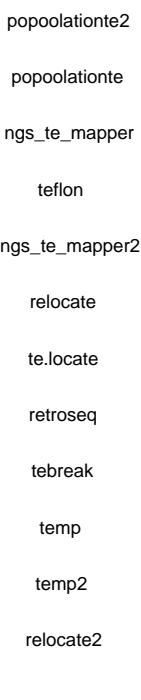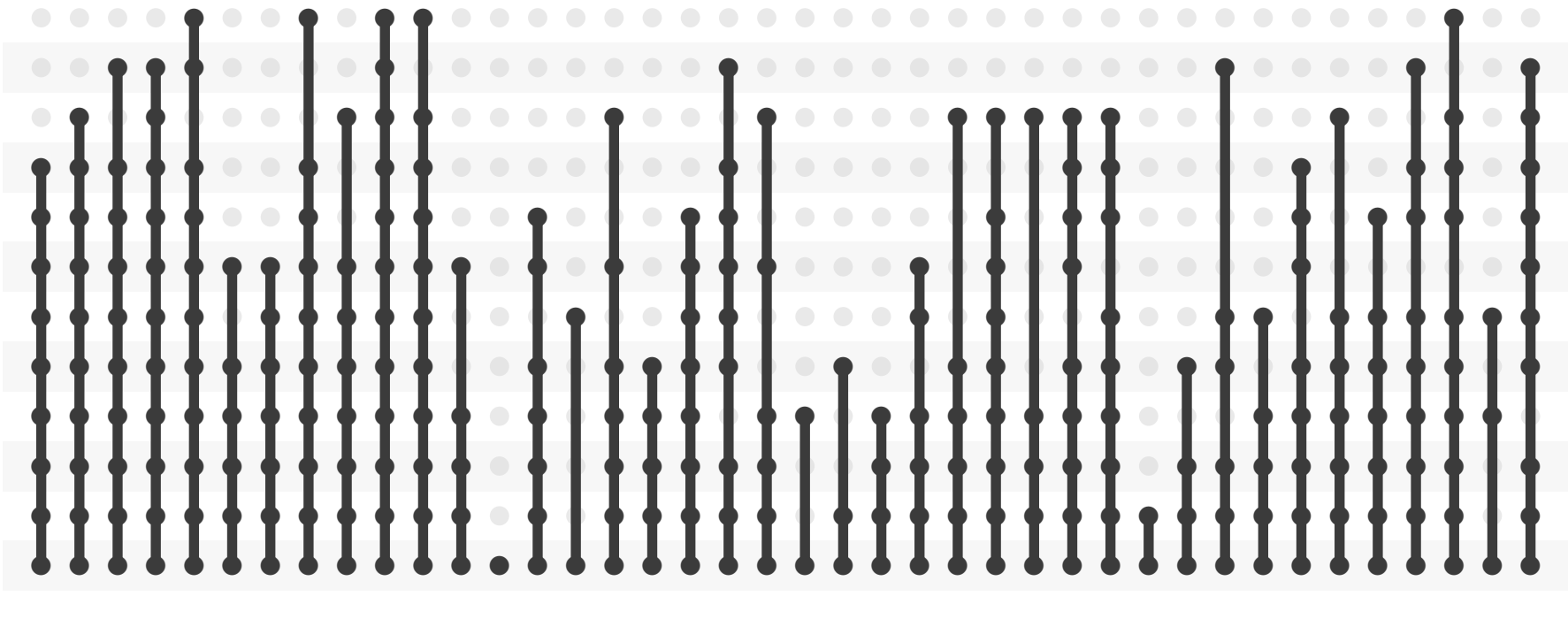

Supplement: Supplementary file 4 — Additional file 4. Overlaps between numbers of non-reference TEs predicted by McClintock component methods in simulated data. UpSet plots visualizing overlaps among component methods for true positive predictions at different window sizes and fold-coverages for Simulations 3 and 4. [file 13100_2023_296_MOESM4_ESM.zip › intersection/sim3_upsetplot_50x_300.pdf]

Simulation 4 Coverage 12x Window 5bp

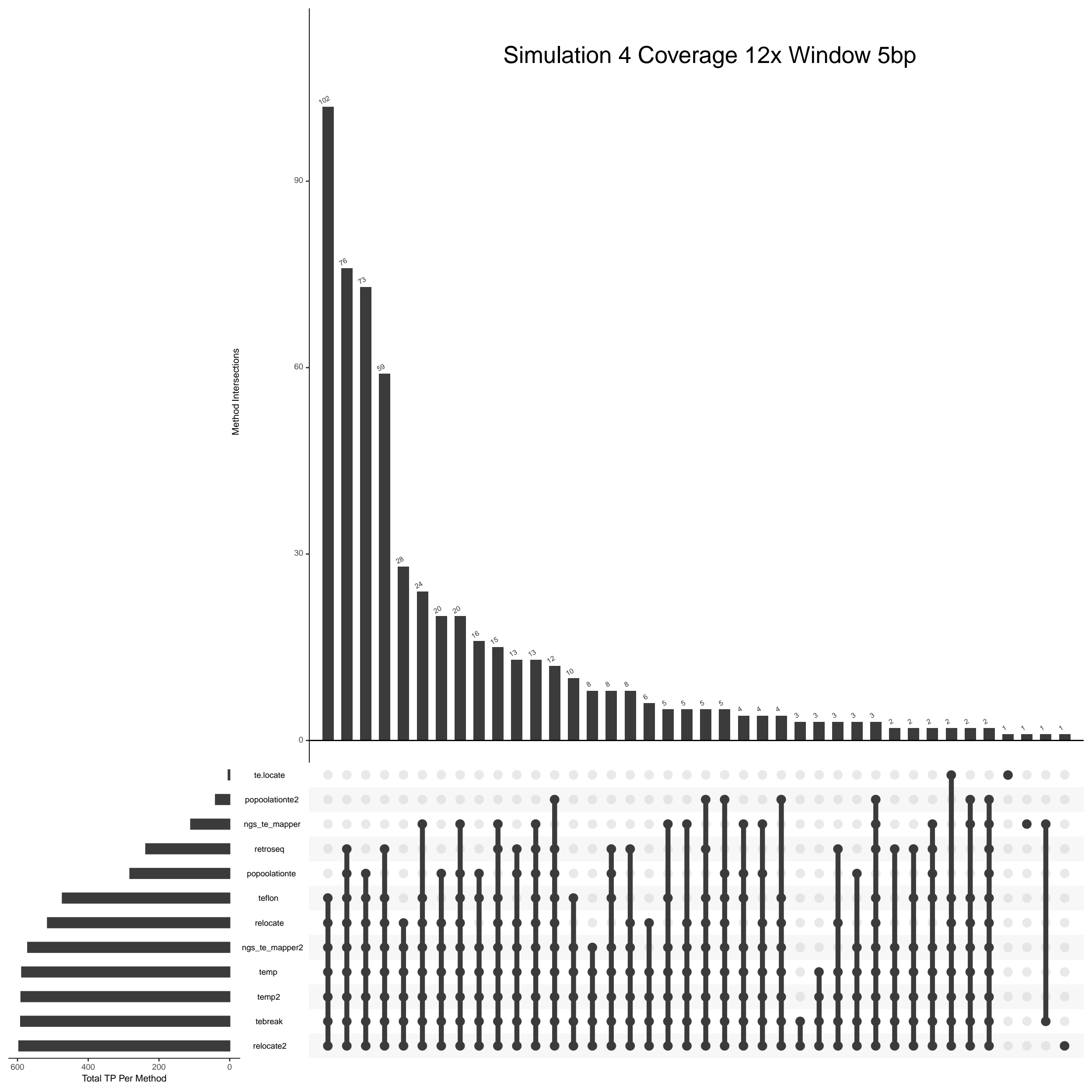

Supplement: Supplementary file 4 — Additional file 4. Overlaps between numbers of non-reference TEs predicted by McClintock component methods in simulated data. UpSet plots visualizing overlaps among component methods for true positive predictions at different window sizes and fold-coverages for Simulations 3 and 4. [file 13100_2023_296_MOESM4_ESM.zip › intersection/sim4_upsetplot_12x_5.pdf]

Simulation 3 Coverage 12 Window 0

Method Intersections

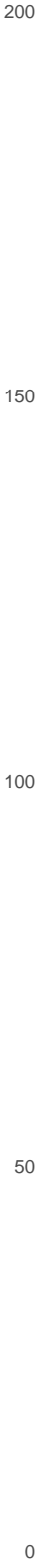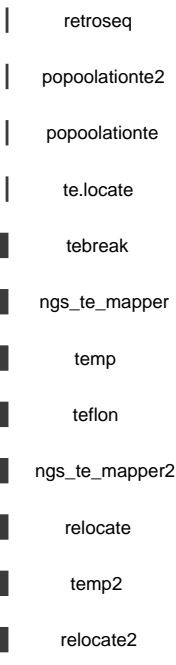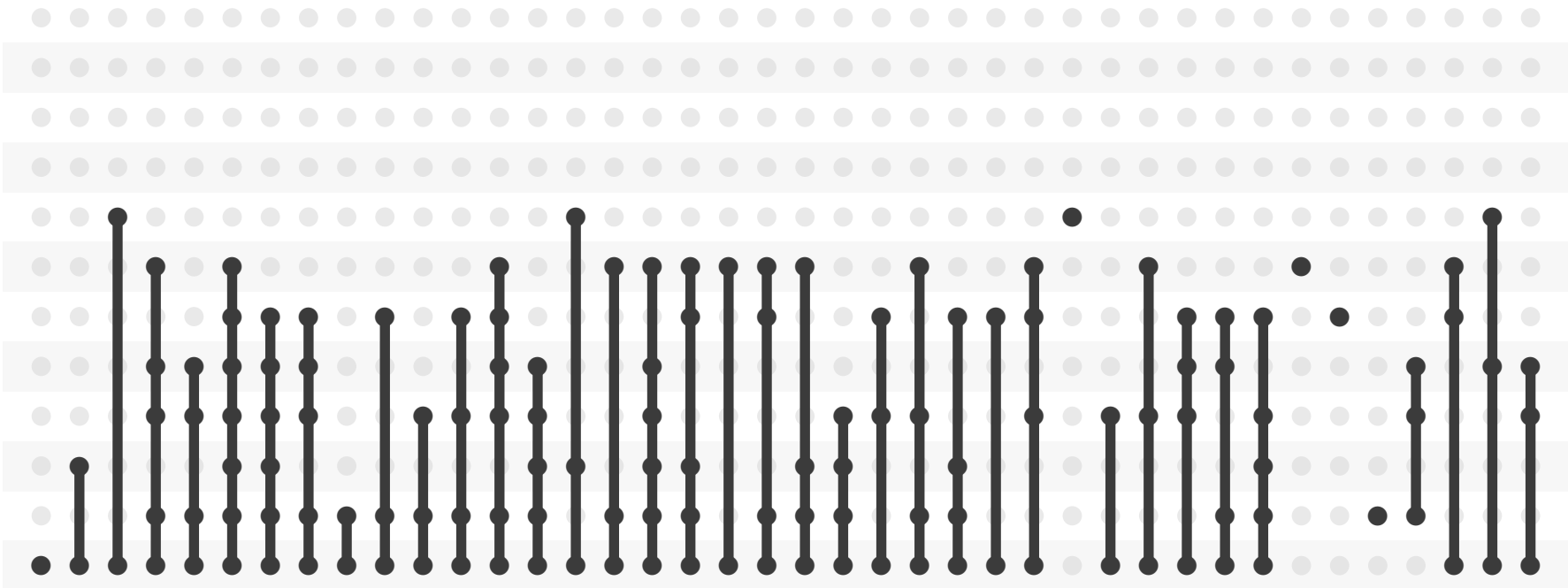

Supplement: Supplementary file 4 — Additional file 4. Overlaps between numbers of non-reference TEs predicted by McClintock component methods in simulated data. UpSet plots visualizing overlaps among component methods for true positive predictions at different window sizes and fold-coverages for Simulations 3 and 4. [file 13100_2023_296_MOESM4_ESM.zip › intersection/sim3_upsetplot_12x_0.pdf]

Simulation 3 Coverage 6 Window 0

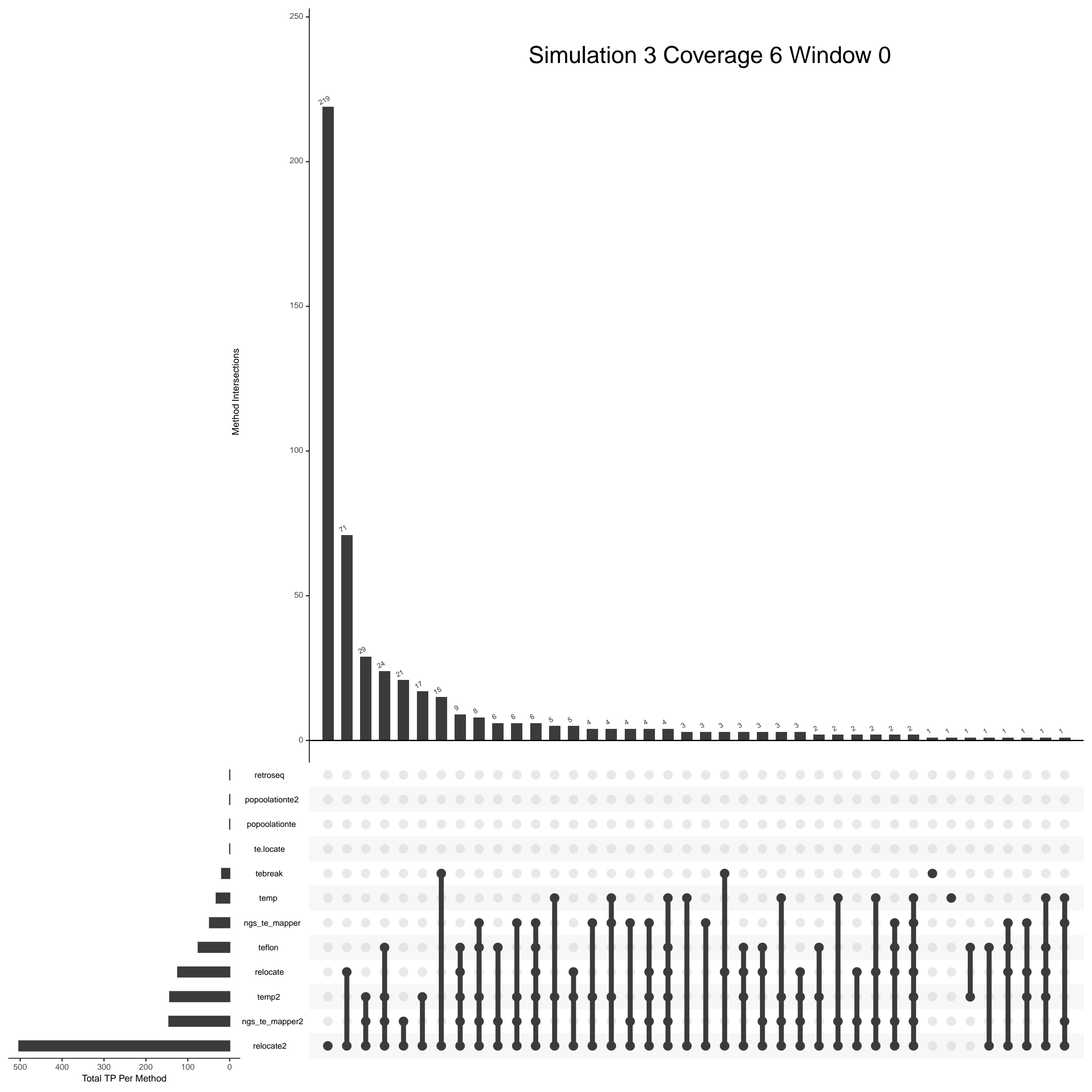

Supplement: Supplementary file 4 — Additional file 4. Overlaps between numbers of non-reference TEs predicted by McClintock component methods in simulated data. UpSet plots visualizing overlaps among component methods for true positive predictions at different window sizes and fold-coverages for Simulations 3 and 4. [file 13100_2023_296_MOESM4_ESM.zip › intersection/sim3_upsetplot_6x_0.pdf]

Simulation 3 Coverage 12 Window 100

Method Intersections

40  
30  
20  
10  
0

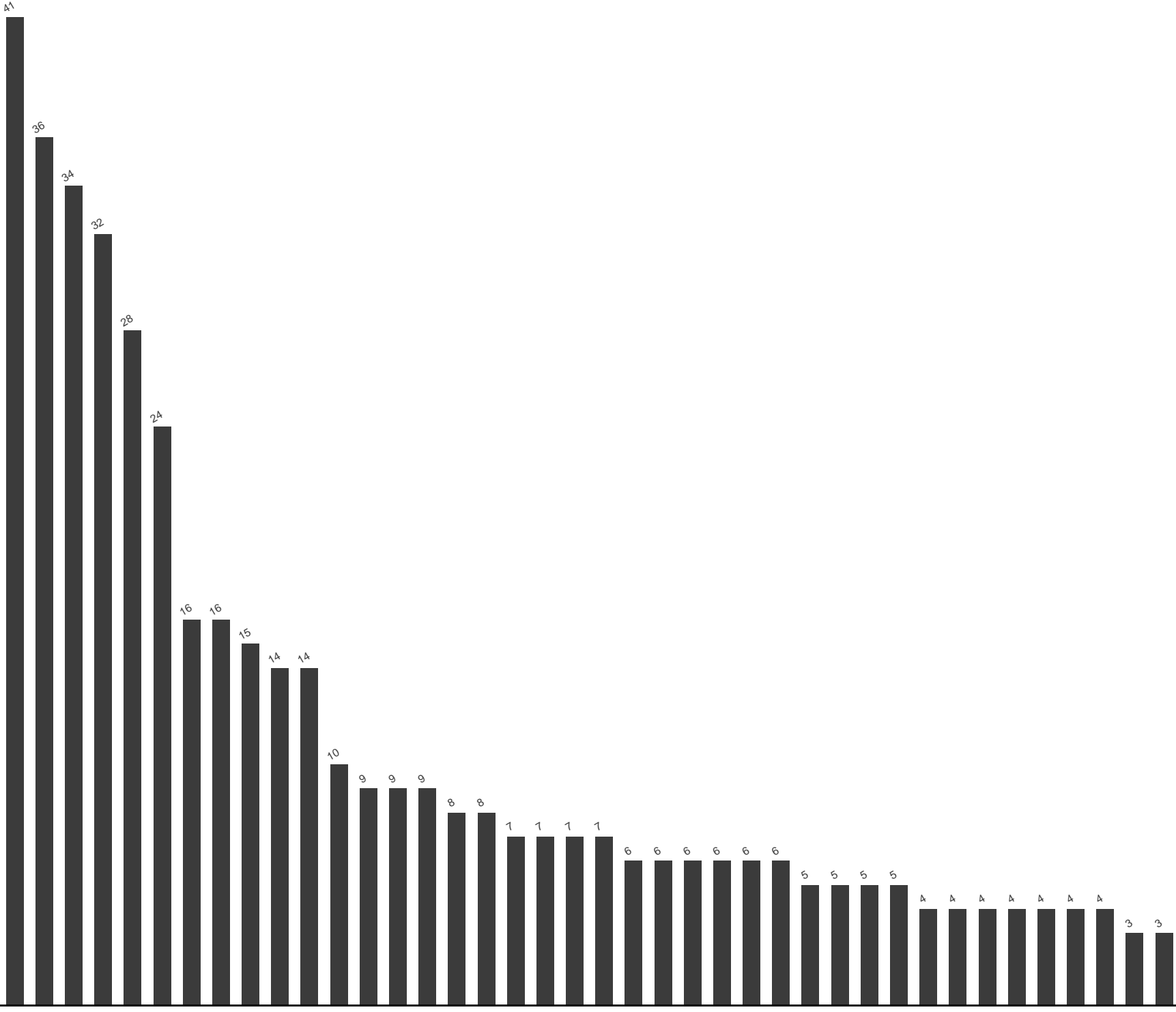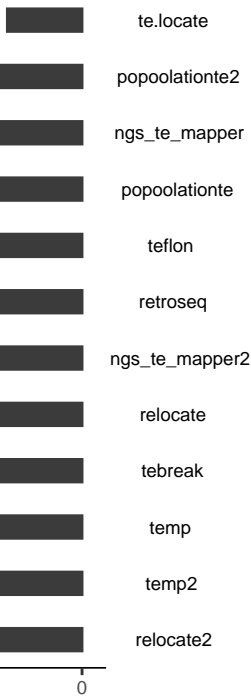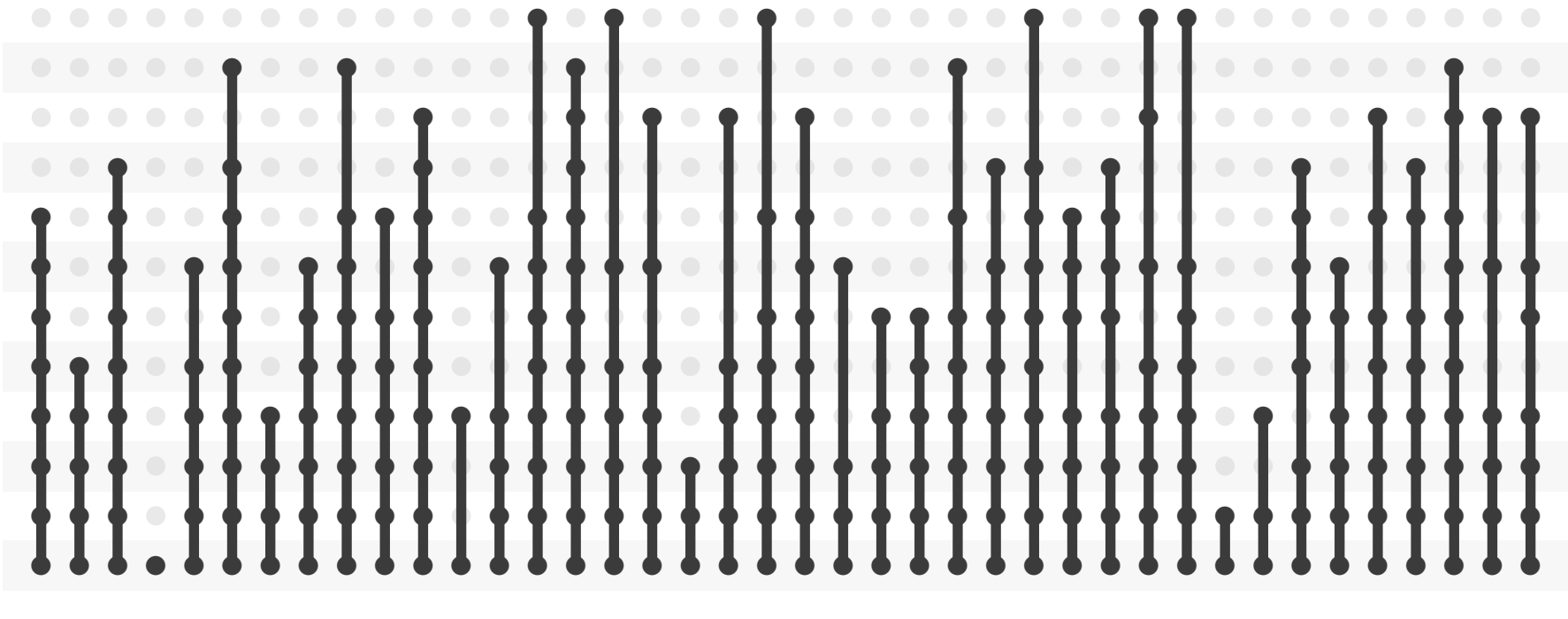

Total TP Per Method

Supplement: Supplementary file 4 — Additional file 4. Overlaps between numbers of non-reference TEs predicted by McClintock component methods in simulated data. UpSet plots visualizing overlaps among component methods for true positive predictions at different window sizes and fold-coverages for Simulations 3 and 4. [file 13100_2023_296_MOESM4_ESM.zip › intersection/sim3_upsetplot_12x_100.pdf]

### Simulation 3 Coverage 12 Window 5

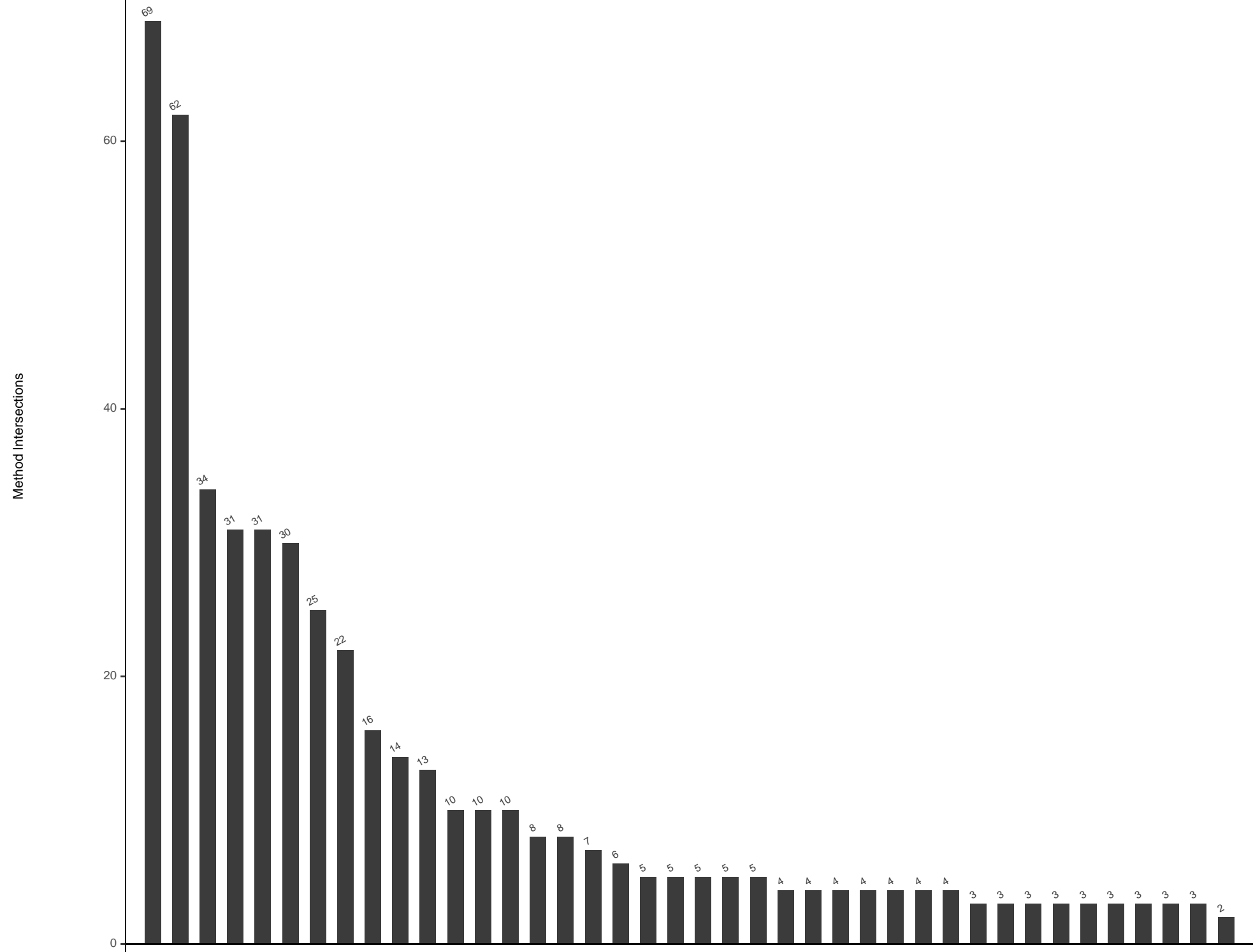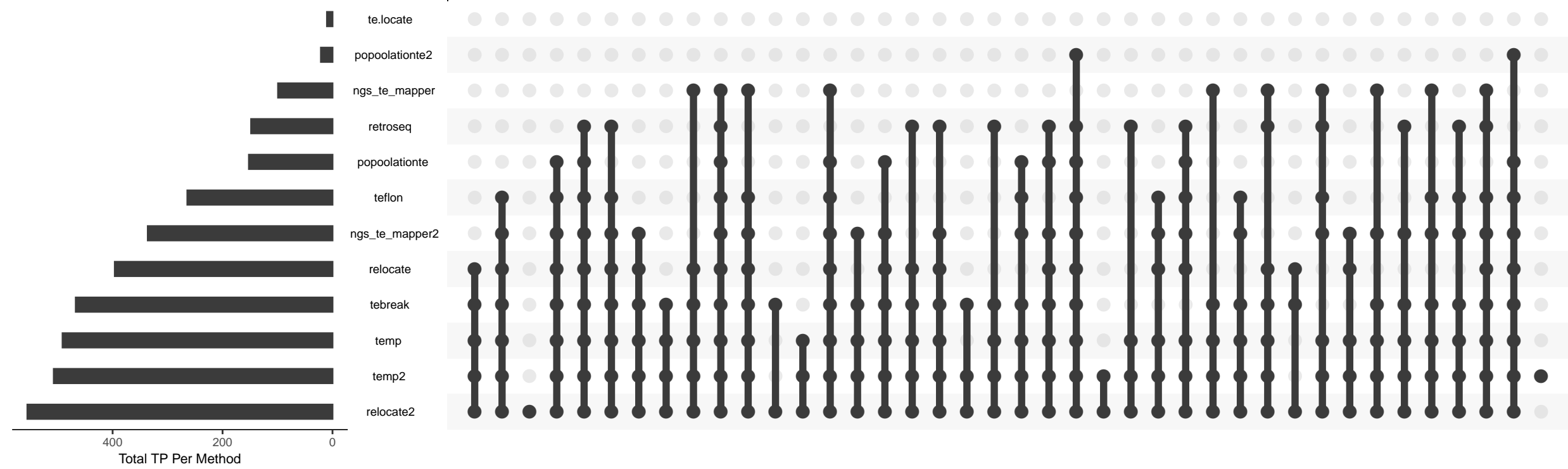

Supplement: Supplementary file 4 — Additional file 4. Overlaps between numbers of non-reference TEs predicted by McClintock component methods in simulated data. UpSet plots visualizing overlaps among component methods for true positive predictions at different window sizes and fold-coverages for Simulations 3 and 4. [file 13100_2023_296_MOESM4_ESM.zip › intersection/sim3_upsetplot_12x_5.pdf]

Simulation 4 Coverage 12x Window 0bp

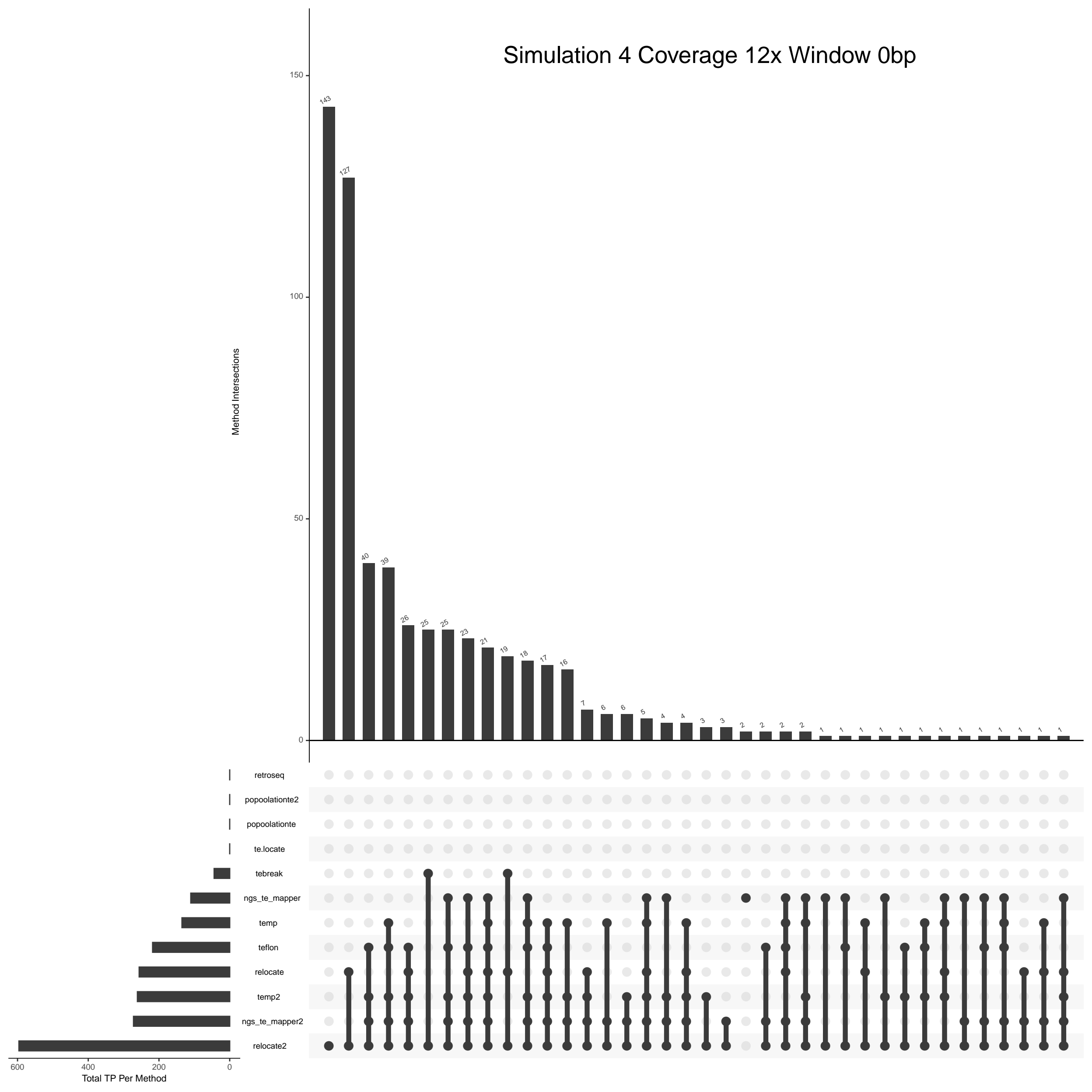

Supplement: Supplementary file 4 — Additional file 4. Overlaps between numbers of non-reference TEs predicted by McClintock component methods in simulated data. UpSet plots visualizing overlaps among component methods for true positive predictions at different window sizes and fold-coverages for Simulations 3 and 4. [file 13100_2023_296_MOESM4_ESM.zip › intersection/sim4_upsetplot_12x_0.pdf]

Simulation 3 Coverage 3 Window 100

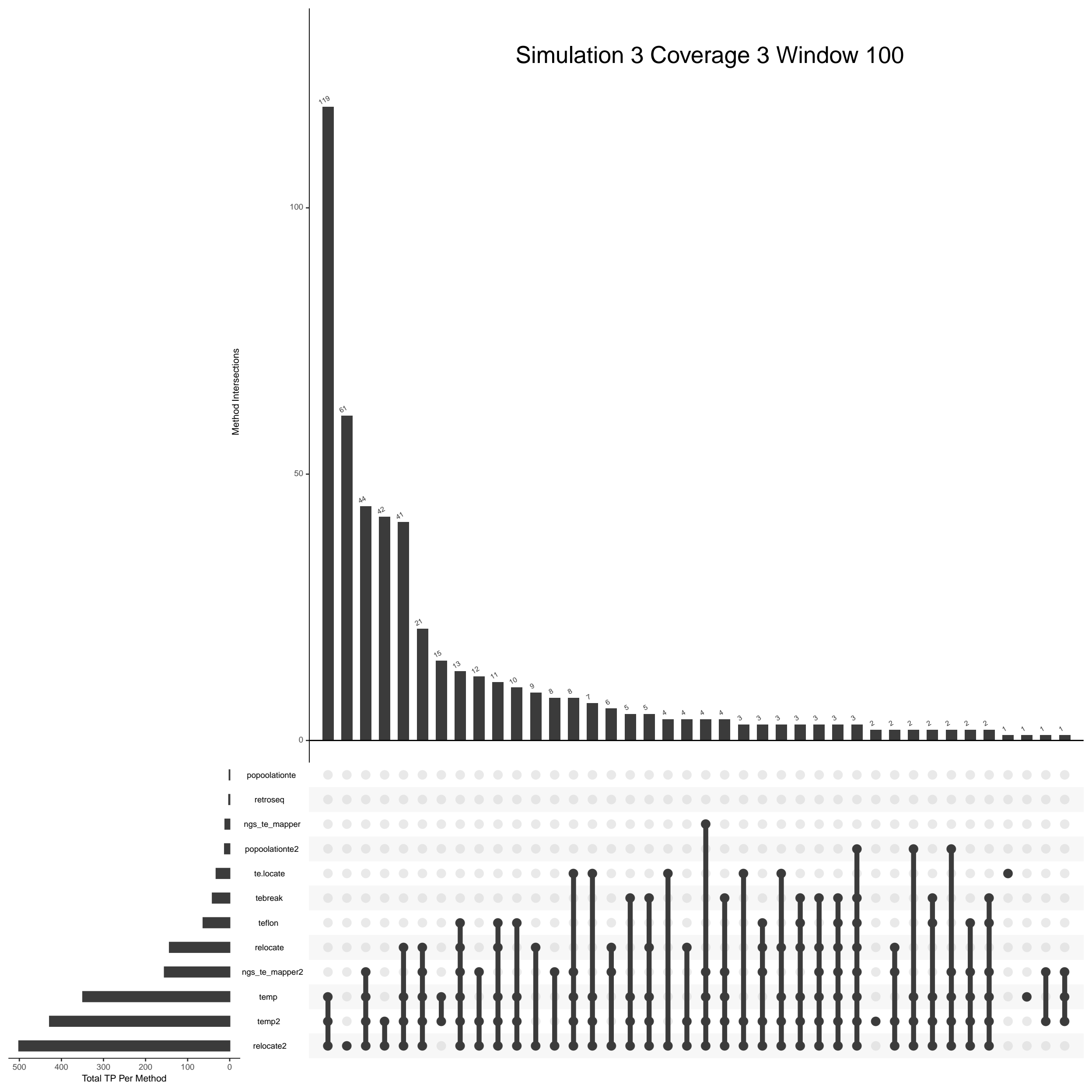

Supplement: Supplementary file 4 — Additional file 4. Overlaps between numbers of non-reference TEs predicted by McClintock component methods in simulated data. UpSet plots visualizing overlaps among component methods for true positive predictions at different window sizes and fold-coverages for Simulations 3 and 4. [file 13100_2023_296_MOESM4_ESM.zip › intersection/sim3_upsetplot_3x_100.pdf]

Simulation 3 Coverage 3 Window 300

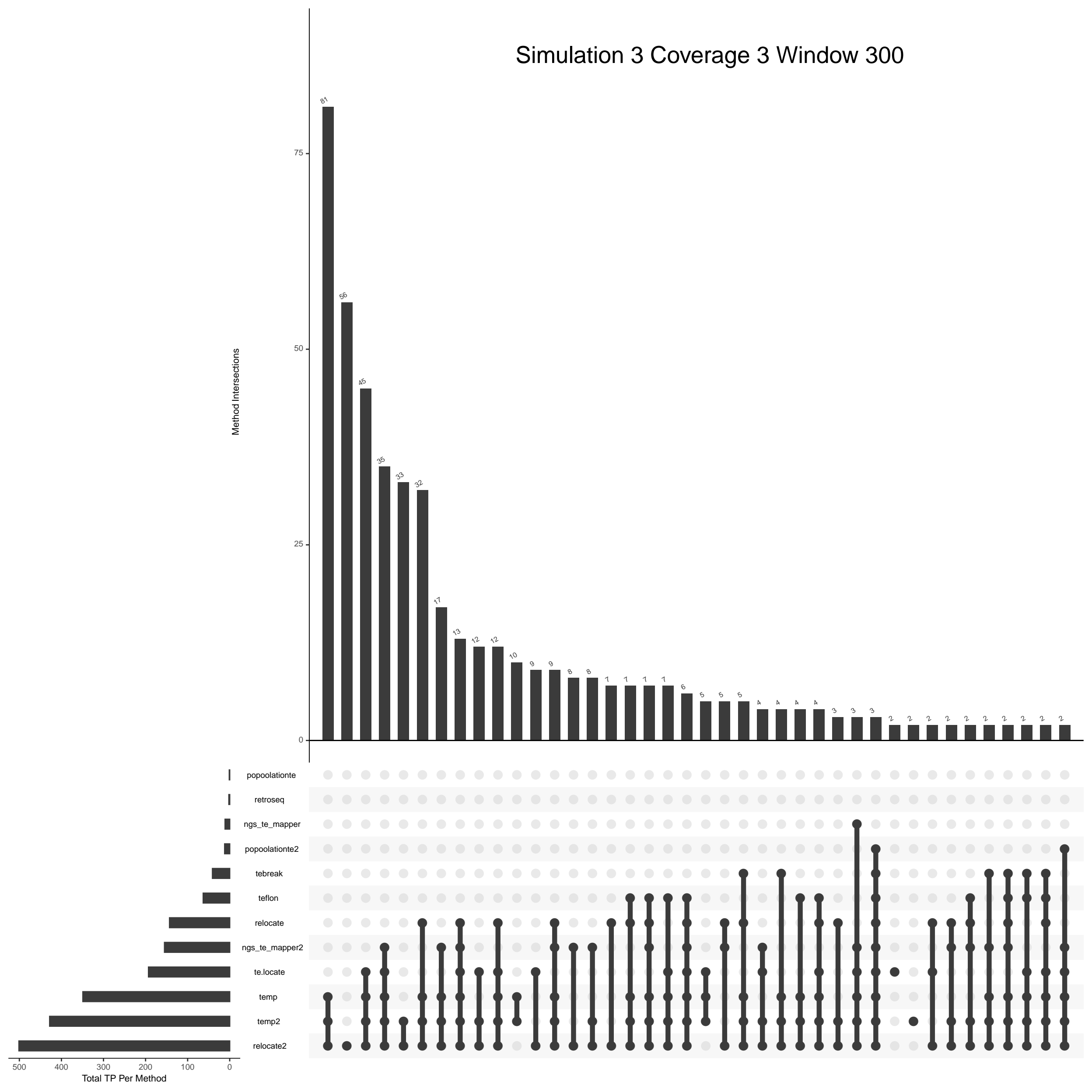

Supplement: Supplementary file 4 — Additional file 4. Overlaps between numbers of non-reference TEs predicted by McClintock component methods in simulated data. UpSet plots visualizing overlaps among component methods for true positive predictions at different window sizes and fold-coverages for Simulations 3 and 4. [file 13100_2023_296_MOESM4_ESM.zip › intersection/sim3_upsetplot_3x_300.pdf]

Simulation 4 Coverage 25x Window 300bp

Method Intersections

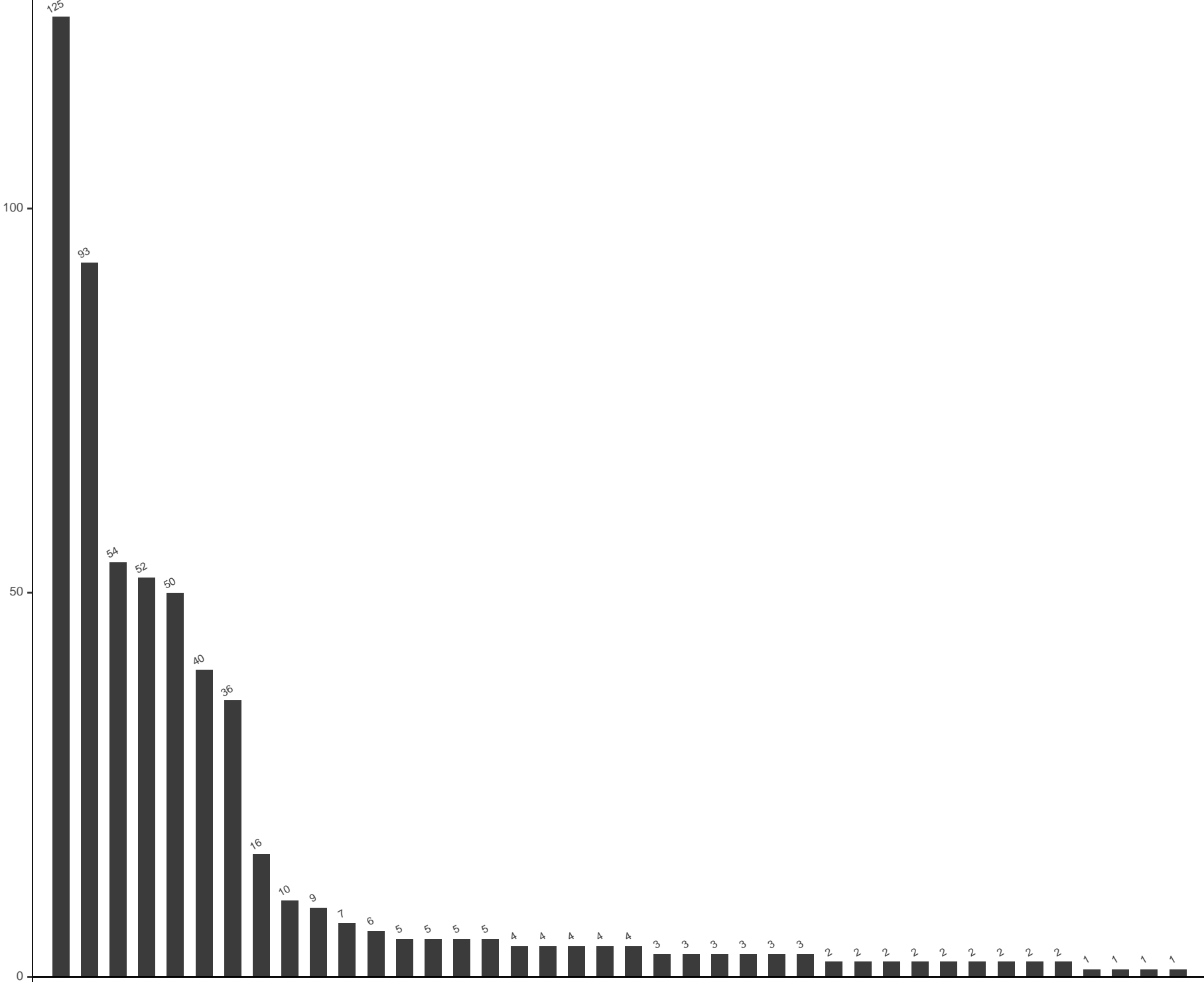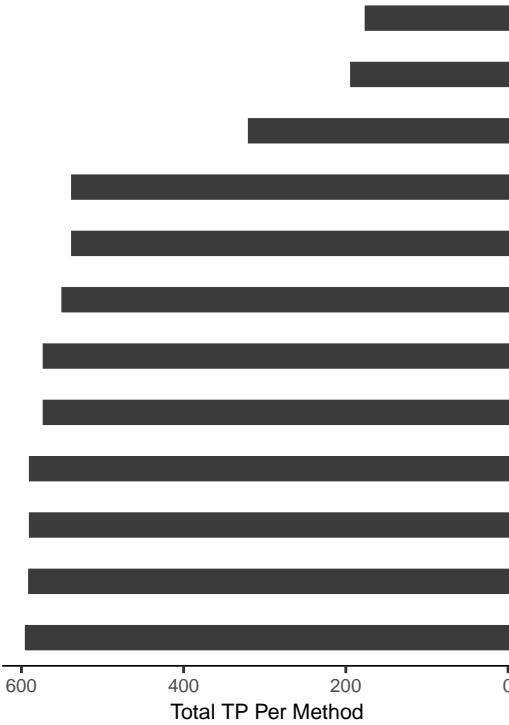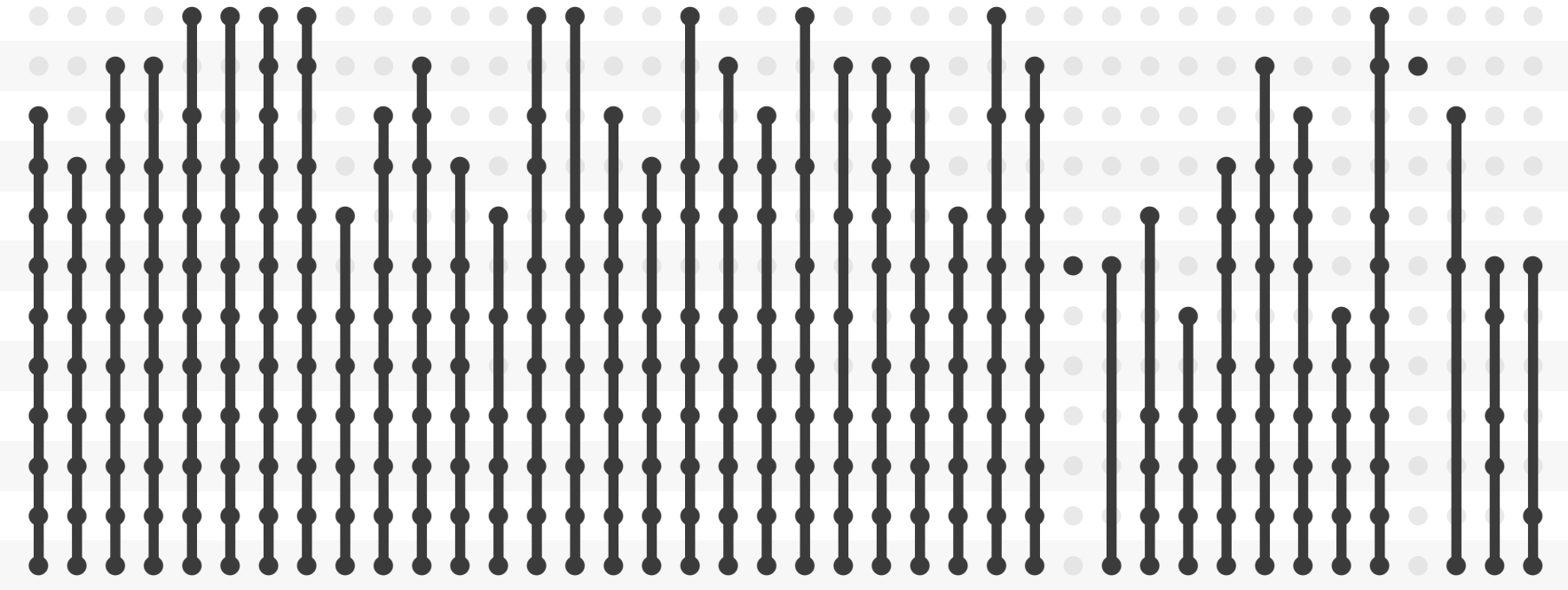

Supplement: Supplementary file 4 — Additional file 4. Overlaps between numbers of non-reference TEs predicted by McClintock component methods in simulated data. UpSet plots visualizing overlaps among component methods for true positive predictions at different window sizes and fold-coverages for Simulations 3 and 4. [file 13100_2023_296_MOESM4_ESM.zip › intersection/sim4_upsetplot_25x_300.pdf]

Simulation 4 Coverage 25x Window 500bp

Method Intersections

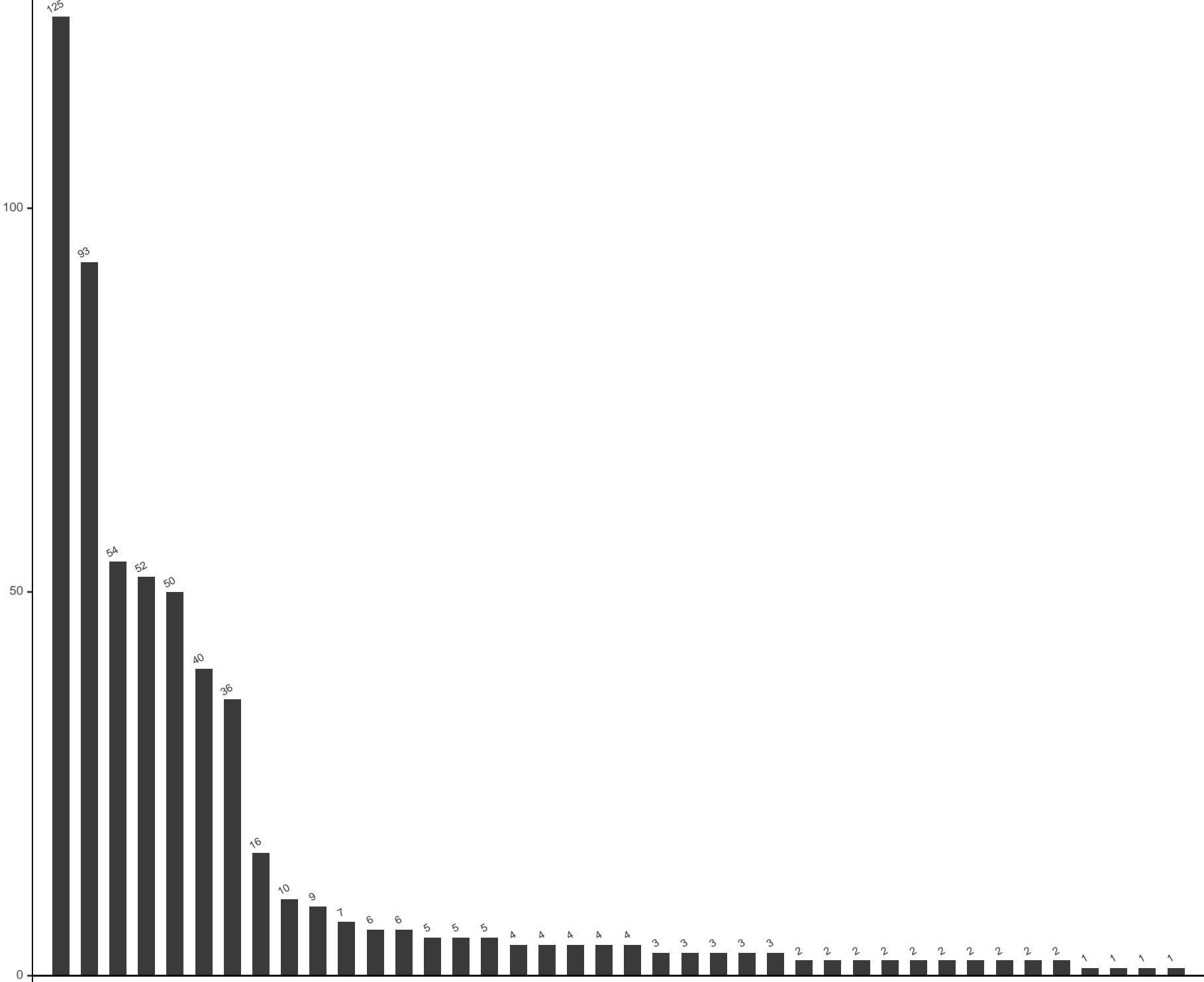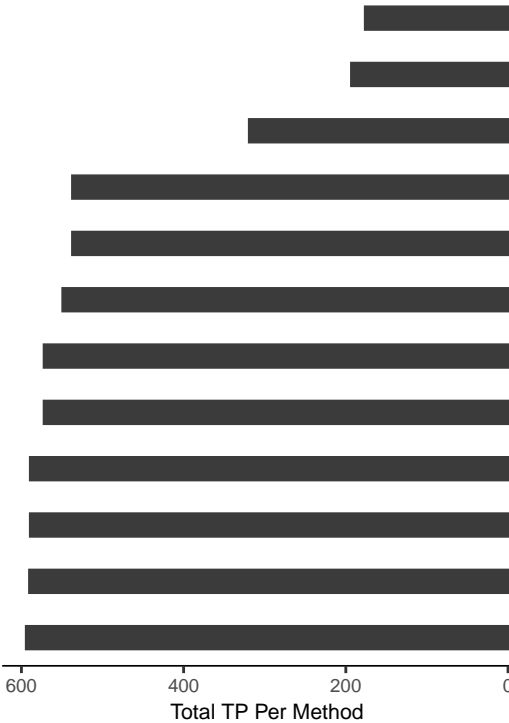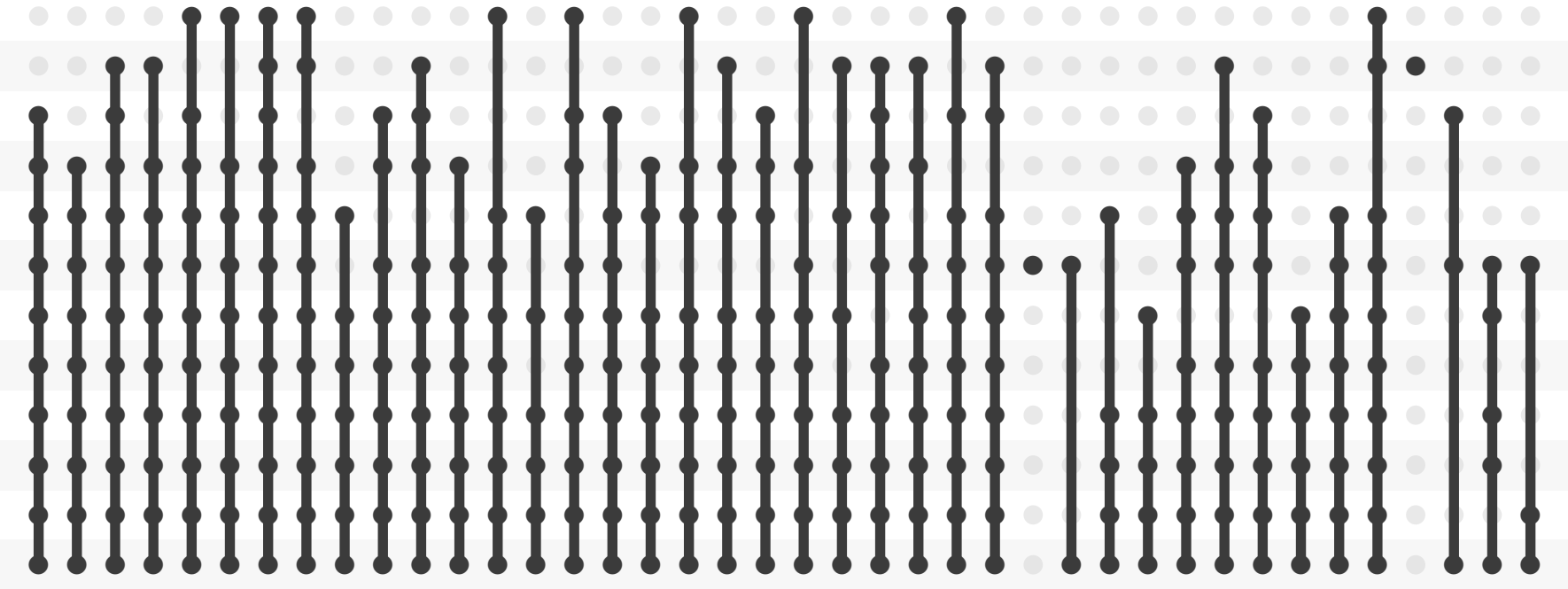

Supplement: Supplementary file 4 — Additional file 4. Overlaps between numbers of non-reference TEs predicted by McClintock component methods in simulated data. UpSet plots visualizing overlaps among component methods for true positive predictions at different window sizes and fold-coverages for Simulations 3 and 4. [file 13100_2023_296_MOESM4_ESM.zip › intersection/sim4_upsetplot_25x_500.pdf]

Simulation 3 Coverage 25 Window 0

Method Intersections

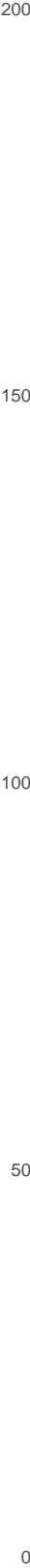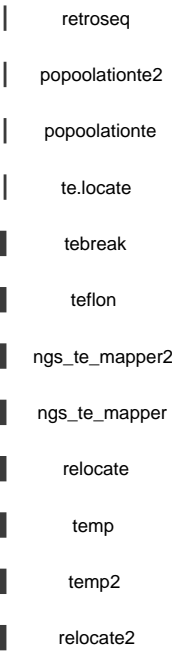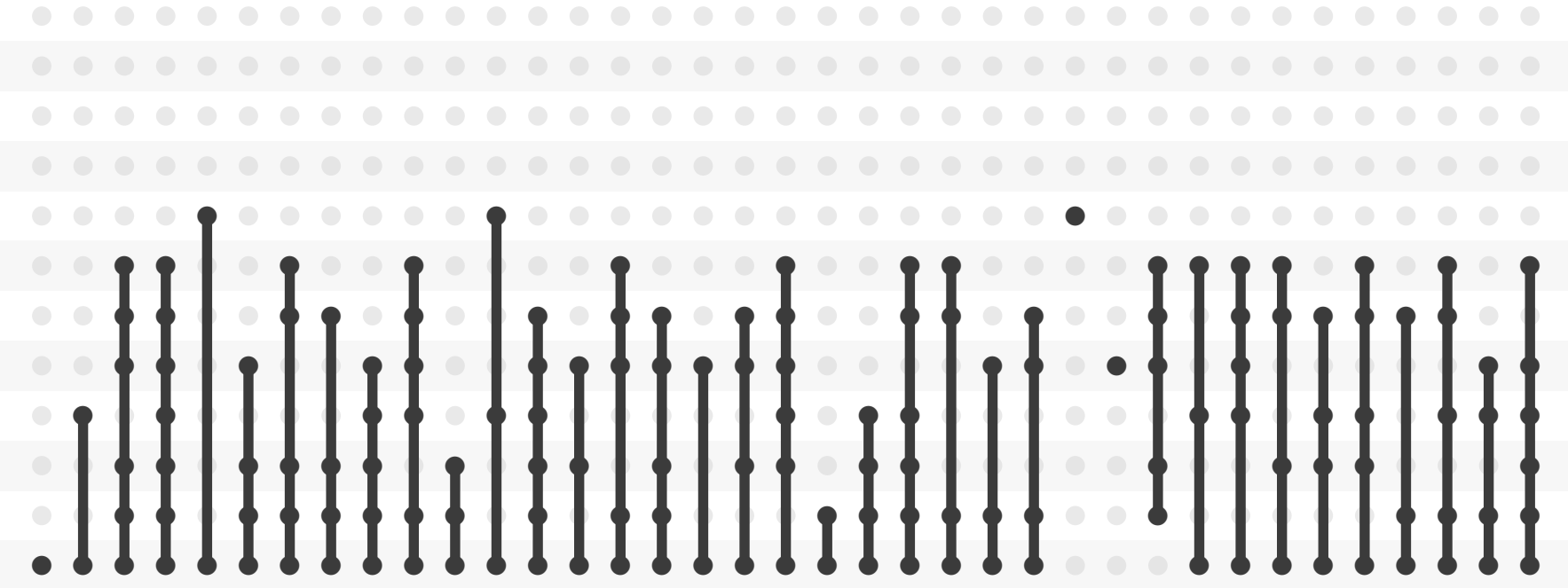

Supplement: Supplementary file 4 — Additional file 4. Overlaps between numbers of non-reference TEs predicted by McClintock component methods in simulated data. UpSet plots visualizing overlaps among component methods for true positive predictions at different window sizes and fold-coverages for Simulations 3 and 4. [file 13100_2023_296_MOESM4_ESM.zip › intersection/sim3_upsetplot_25x_0.pdf]

Simulation 4 Coverage 25x Window 5bp

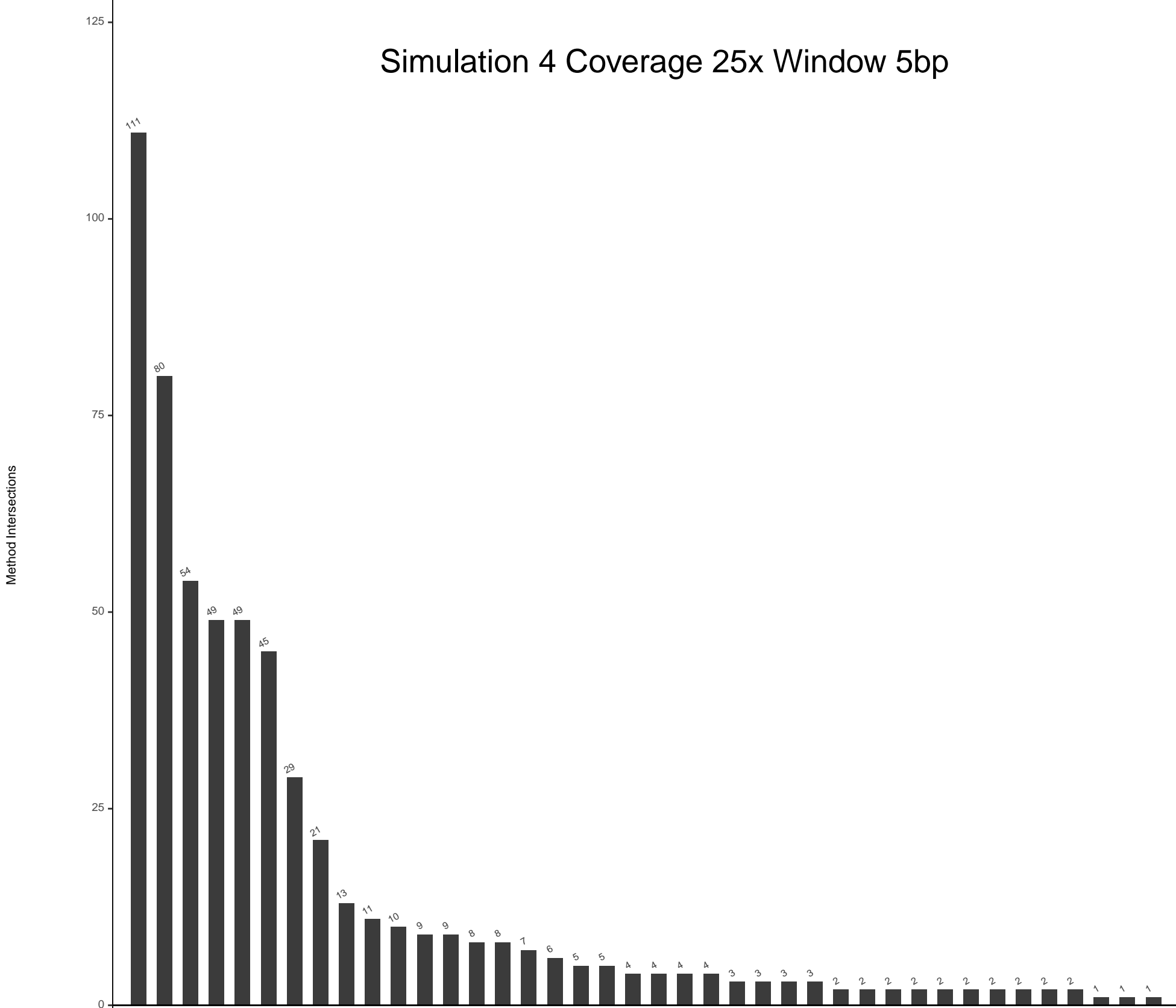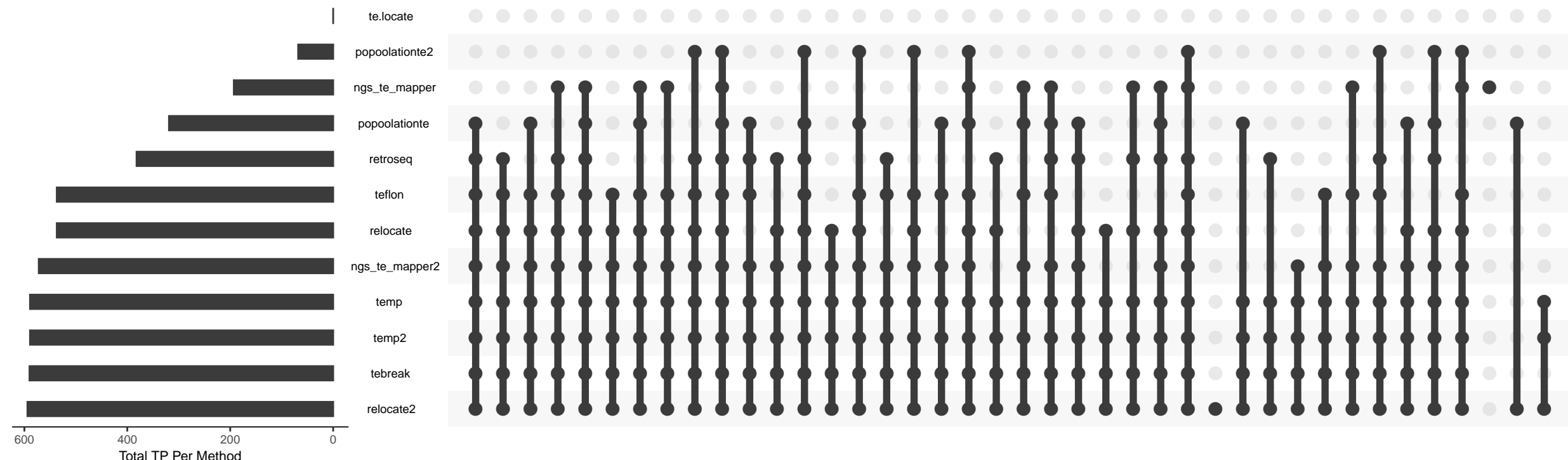

Supplement: Supplementary file 4 — Additional file 4. Overlaps between numbers of non-reference TEs predicted by McClintock component methods in simulated data. UpSet plots visualizing overlaps among component methods for true positive predictions at different window sizes and fold-coverages for Simulations 3 and 4. [file 13100_2023_296_MOESM4_ESM.zip › intersection/sim4_upsetplot_25x_5.pdf]

## Simulation 4 Coverage 6x Window 0bp

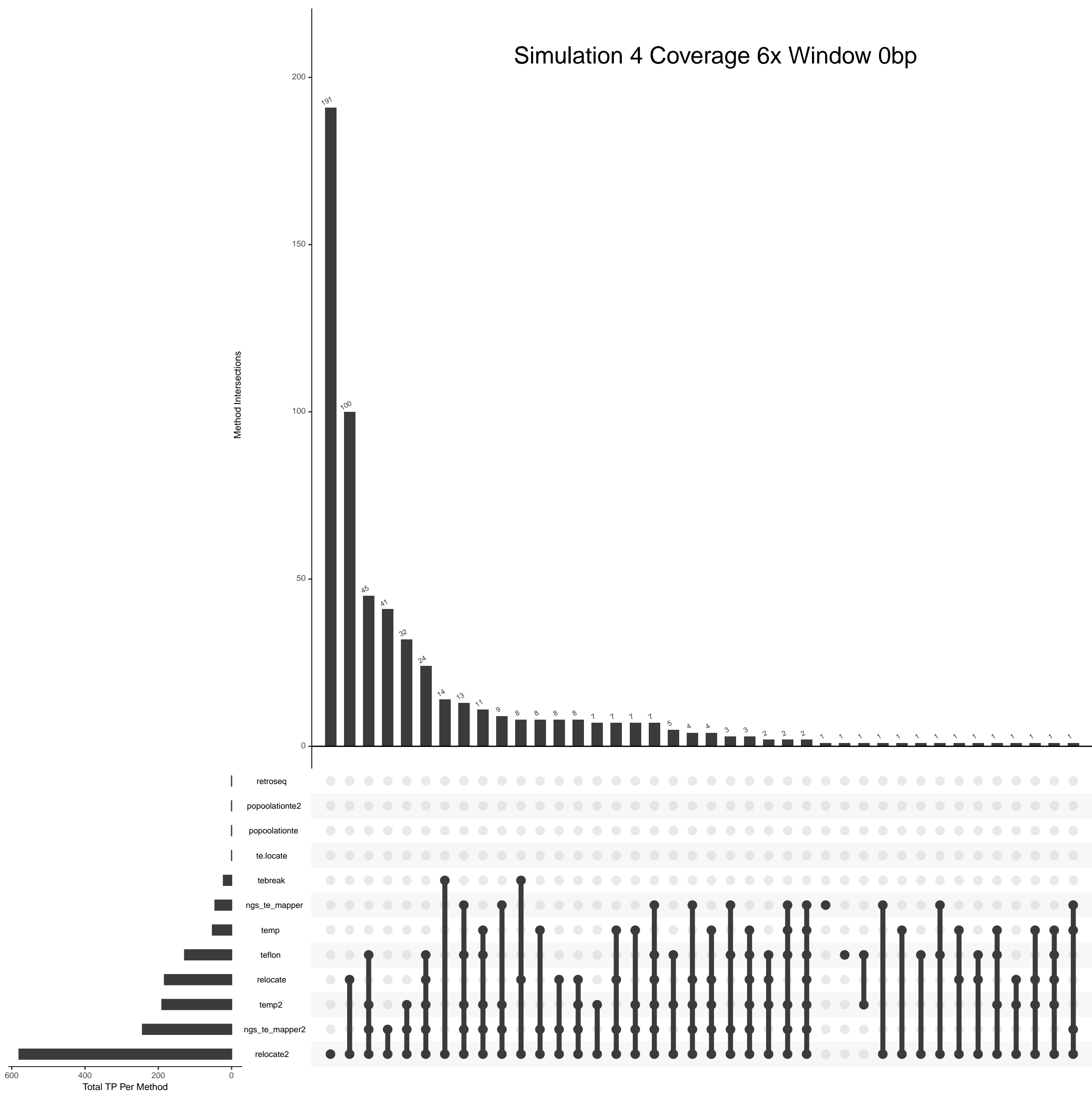

Supplement: Supplementary file 4 — Additional file 4. Overlaps between numbers of non-reference TEs predicted by McClintock component methods in simulated data. UpSet plots visualizing overlaps among component methods for true positive predictions at different window sizes and fold-coverages for Simulations 3 and 4. [file 13100_2023_296_MOESM4_ESM.zip › intersection/sim4_upsetplot_6x_0.pdf]

Simulation 4 Coverage 100x Window 300bp

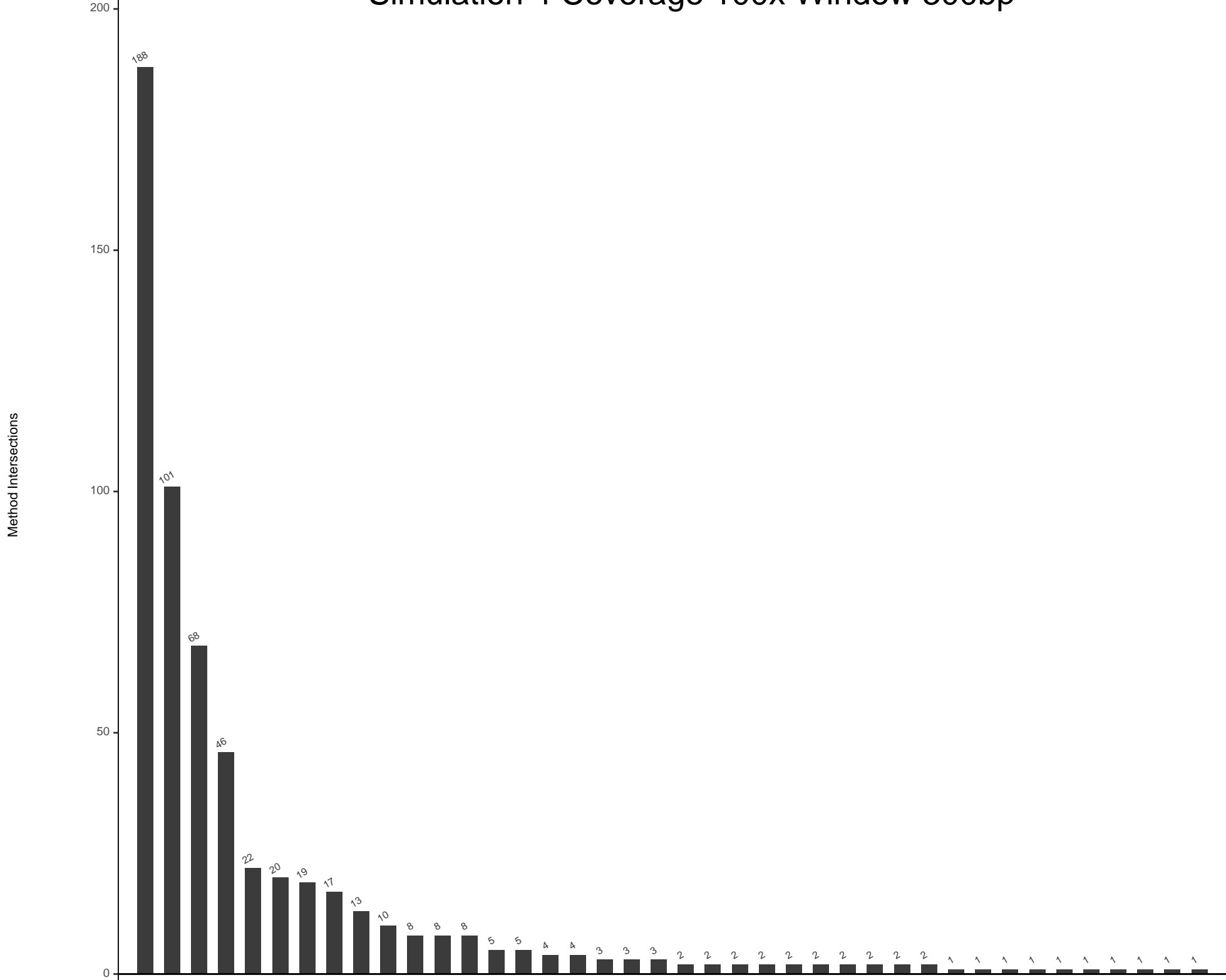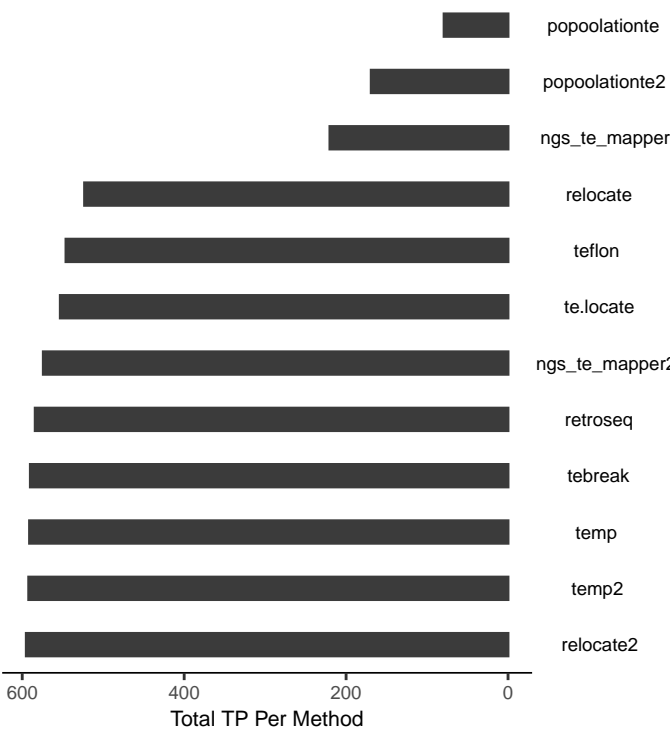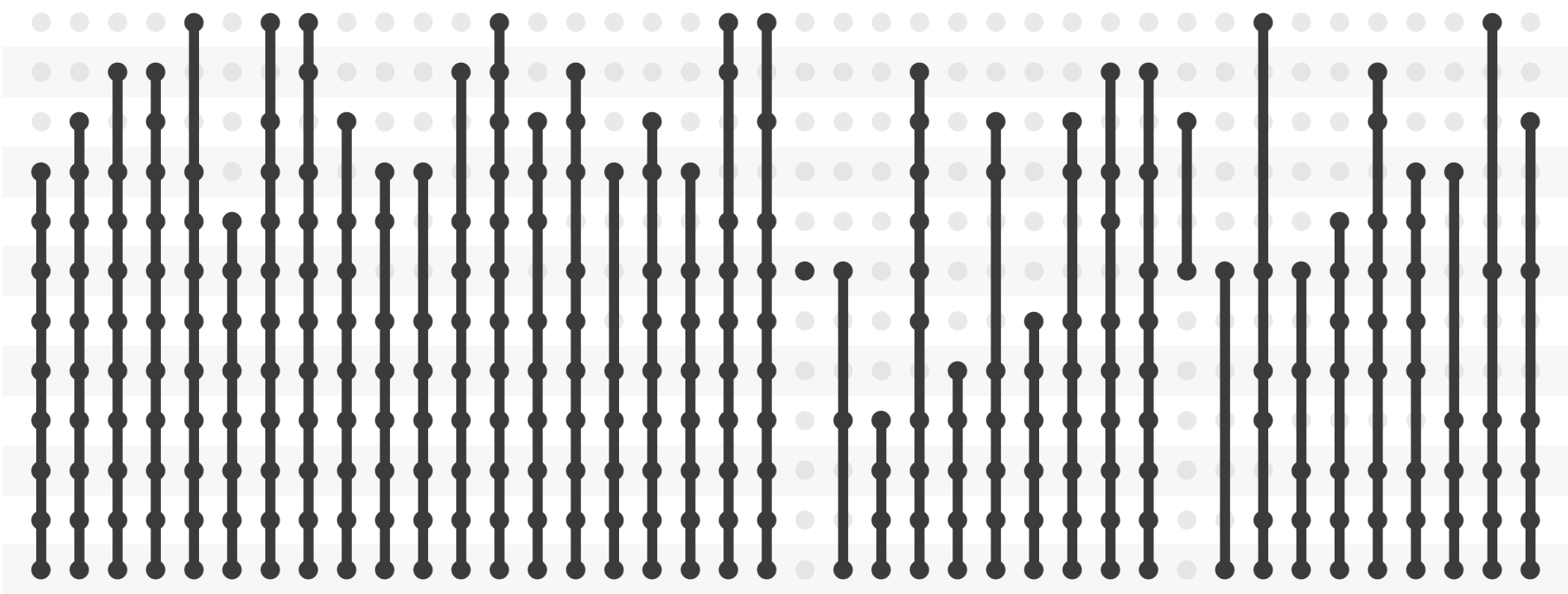

Supplement: Supplementary file 4 — Additional file 4. Overlaps between numbers of non-reference TEs predicted by McClintock component methods in simulated data. UpSet plots visualizing overlaps among component methods for true positive predictions at different window sizes and fold-coverages for Simulations 3 and 4. [file 13100_2023_296_MOESM4_ESM.zip › intersection/sim4_upsetplot_100x_300.pdf]

### Simulation 4 Coverage 3x Window 100bp

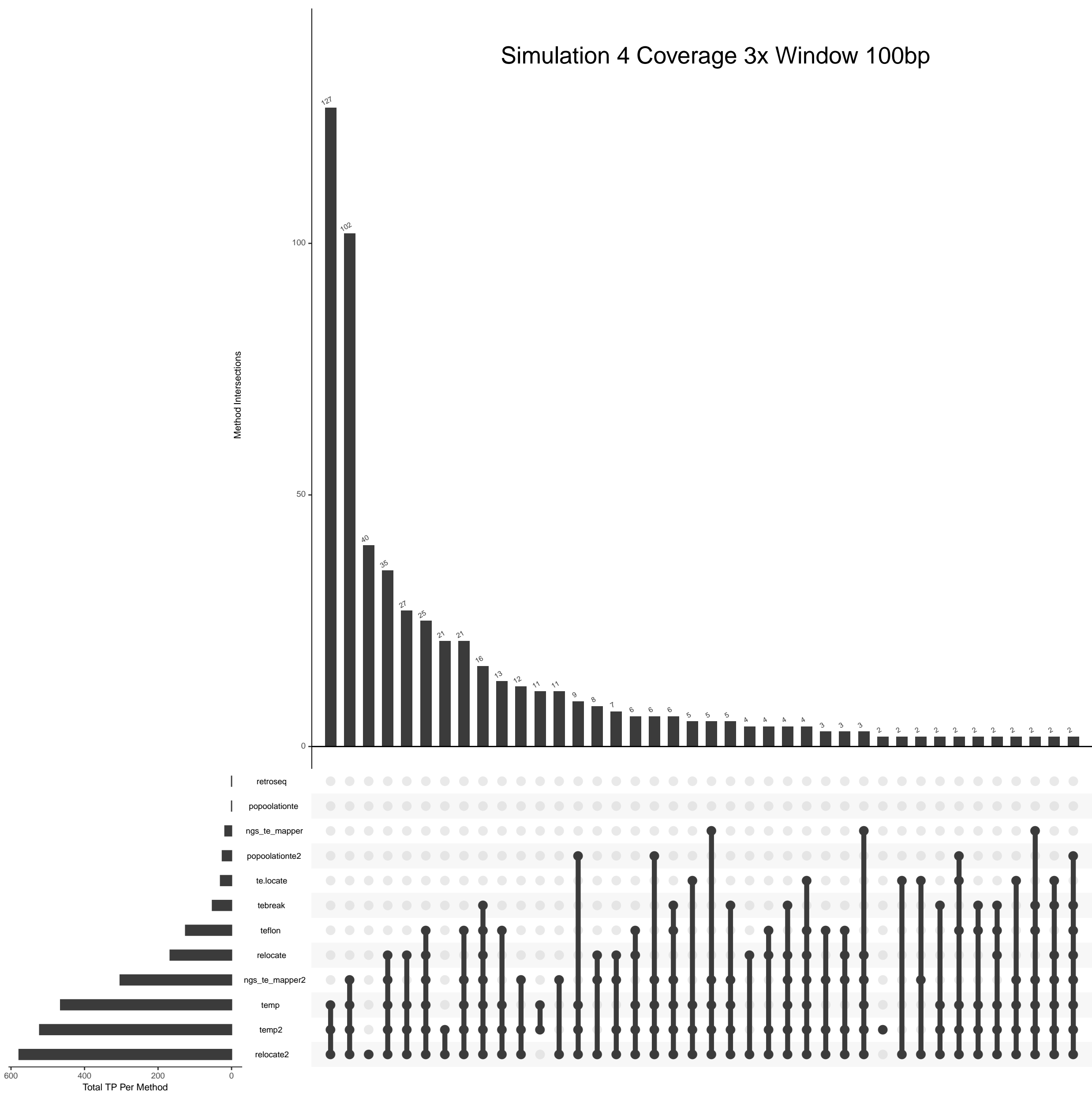

Supplement: Supplementary file 4 — Additional file 4. Overlaps between numbers of non-reference TEs predicted by McClintock component methods in simulated data. UpSet plots visualizing overlaps among component methods for true positive predictions at different window sizes and fold-coverages for Simulations 3 and 4. [file 13100_2023_296_MOESM4_ESM.zip › intersection/sim4_upsetplot_3x_100.pdf]

Simulation 4 Coverage 3x Window 300bp

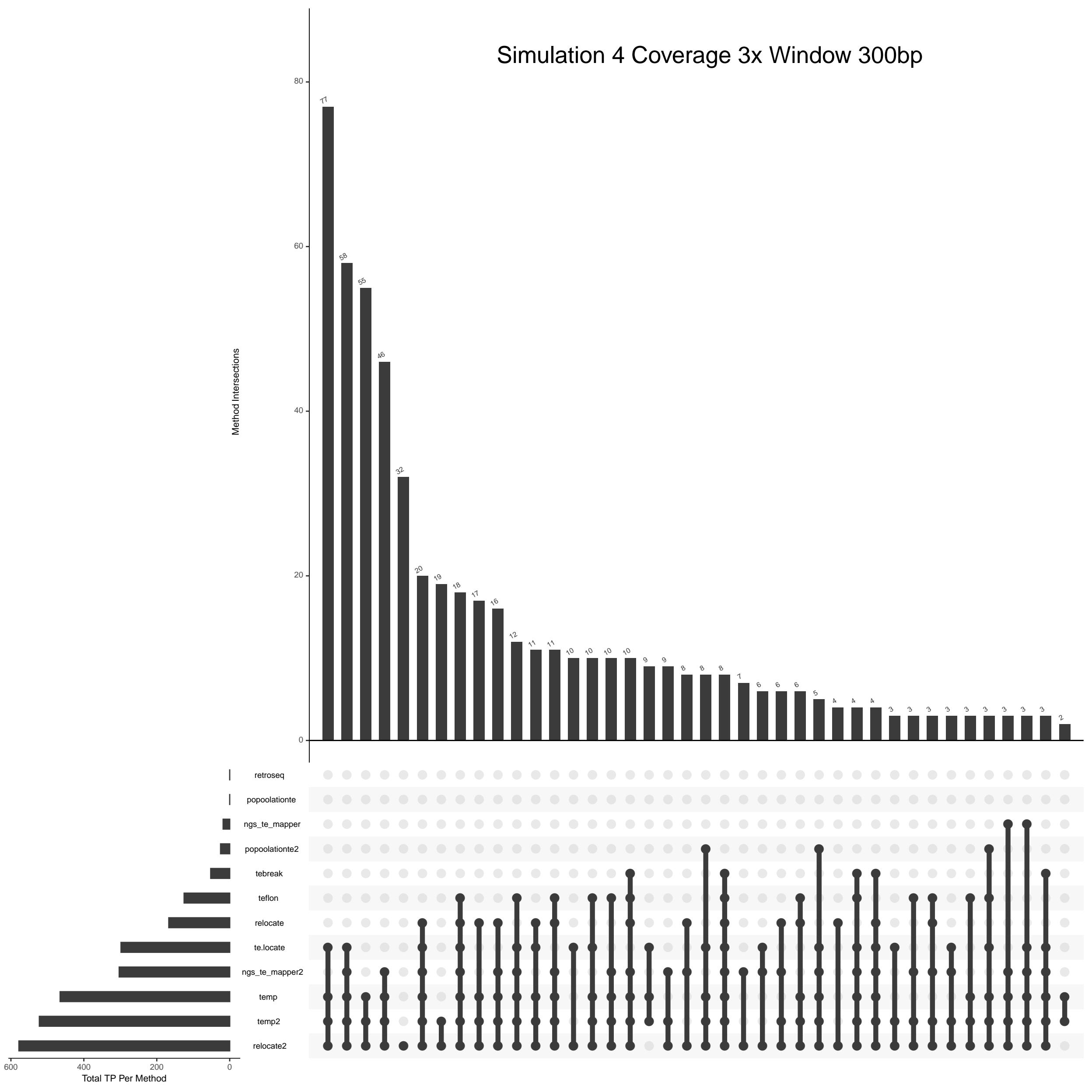

Supplement: Supplementary file 4 — Additional file 4. Overlaps between numbers of non-reference TEs predicted by McClintock component methods in simulated data. UpSet plots visualizing overlaps among component methods for true positive predictions at different window sizes and fold-coverages for Simulations 3 and 4. [file 13100_2023_296_MOESM4_ESM.zip › intersection/sim4_upsetplot_3x_300.pdf]

Simulation 4 Coverage 100x Window 100bp

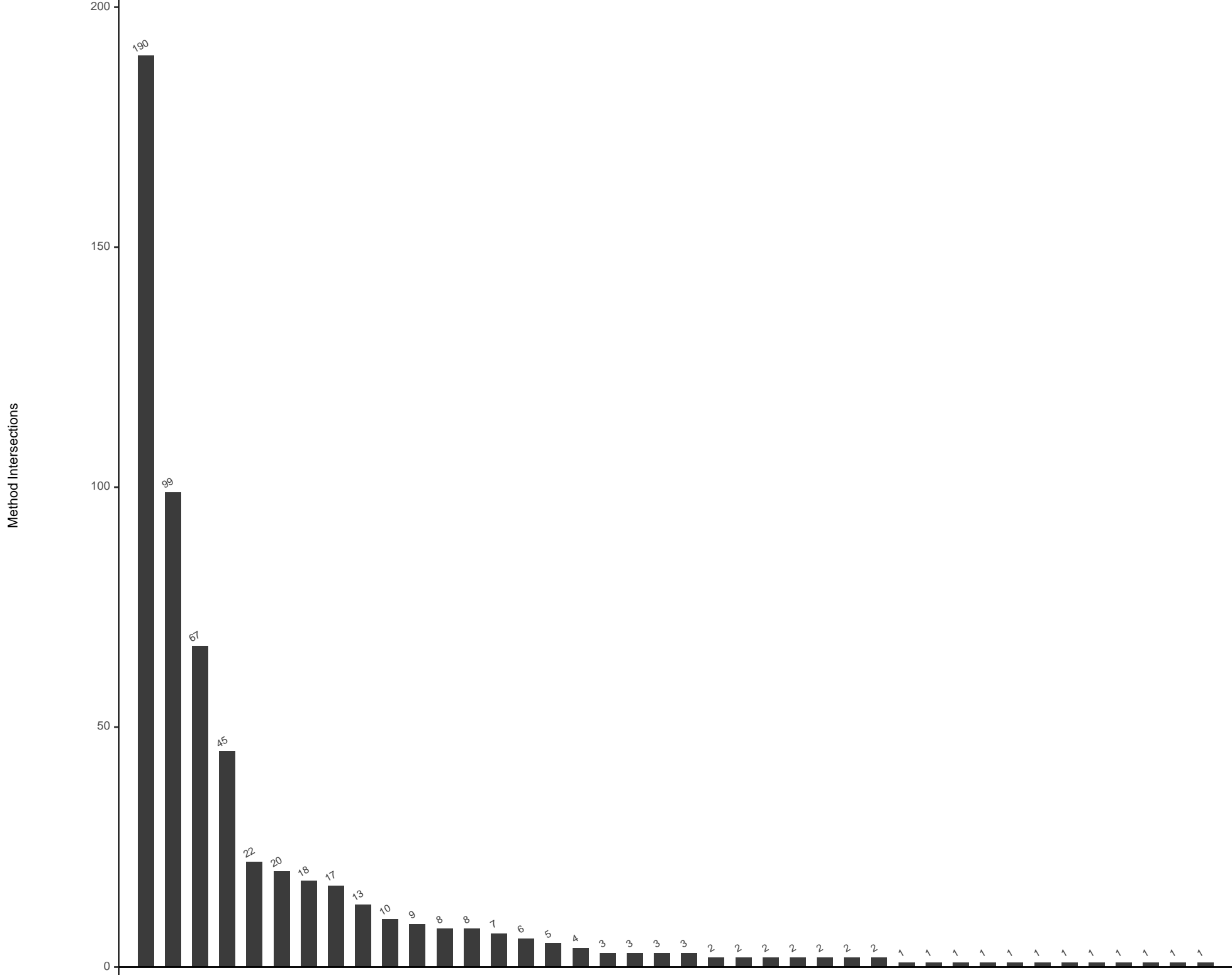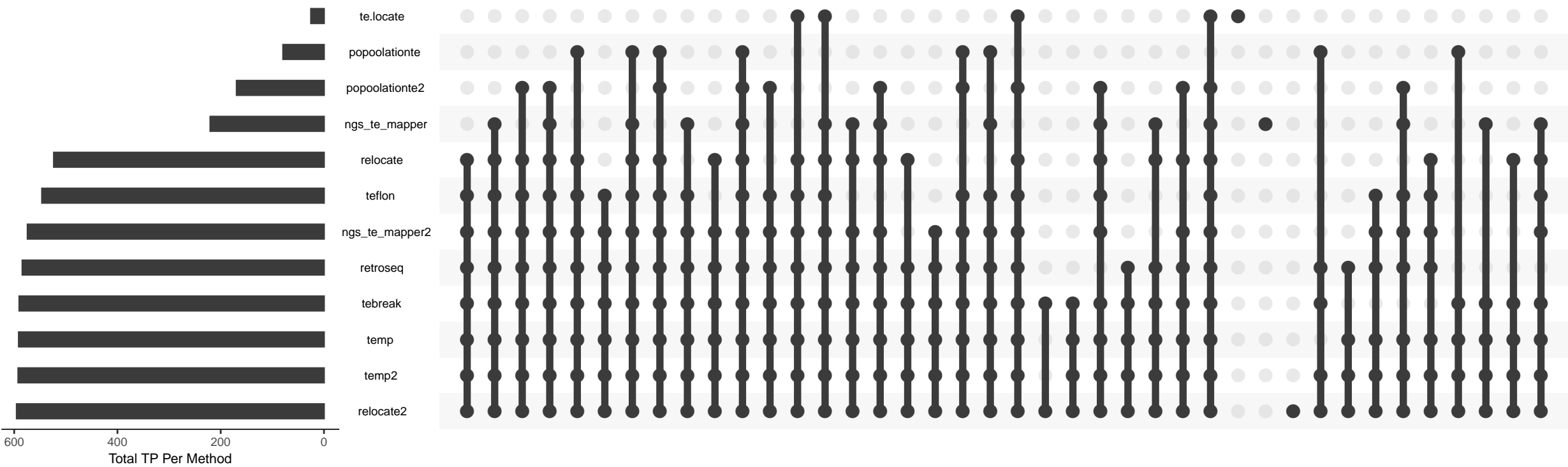

Supplement: Supplementary file 4 — Additional file 4. Overlaps between numbers of non-reference TEs predicted by McClintock component methods in simulated data. UpSet plots visualizing overlaps among component methods for true positive predictions at different window sizes and fold-coverages for Simulations 3 and 4. [file 13100_2023_296_MOESM4_ESM.zip › intersection/sim4_upsetplot_100x_100.pdf]

Simulation 3 Coverage 100 Window 300

Method Intersections

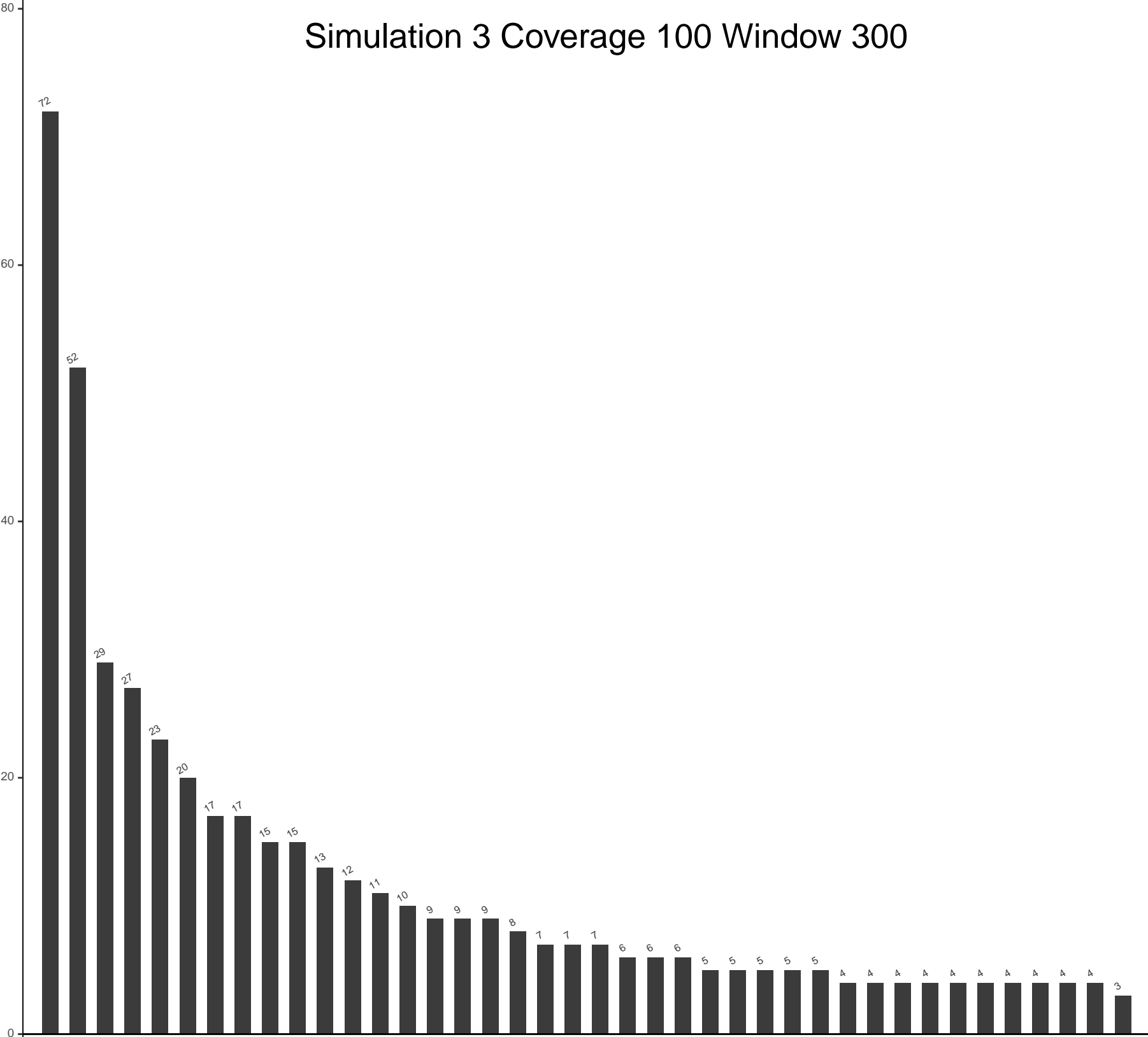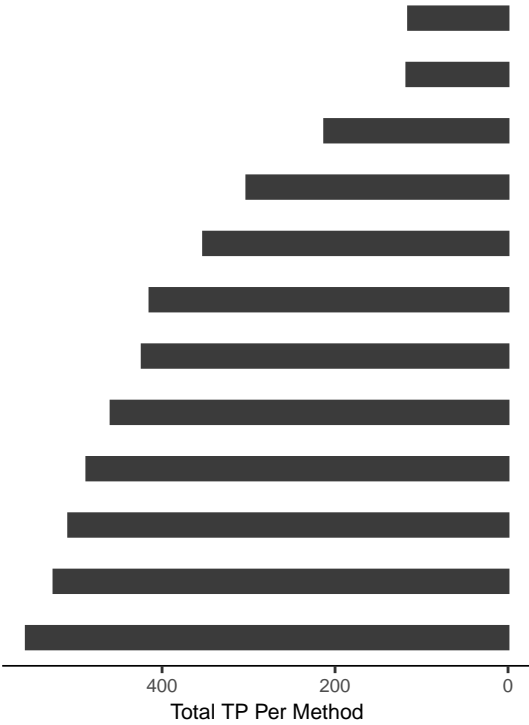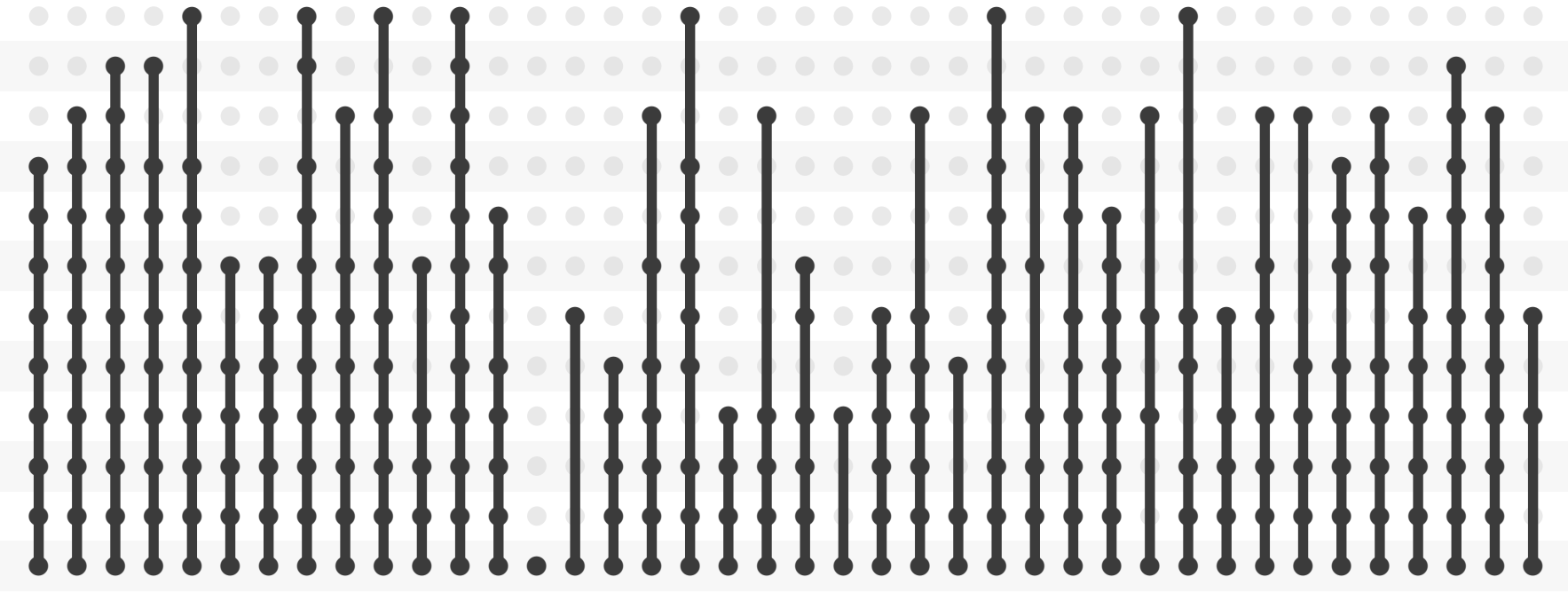

Supplement: Supplementary file 4 — Additional file 4. Overlaps between numbers of non-reference TEs predicted by McClintock component methods in simulated data. UpSet plots visualizing overlaps among component methods for true positive predictions at different window sizes and fold-coverages for Simulations 3 and 4. [file 13100_2023_296_MOESM4_ESM.zip › intersection/sim3_upsetplot_100x_300.pdf]

Simulation 4 Coverage 3x Window 500bp

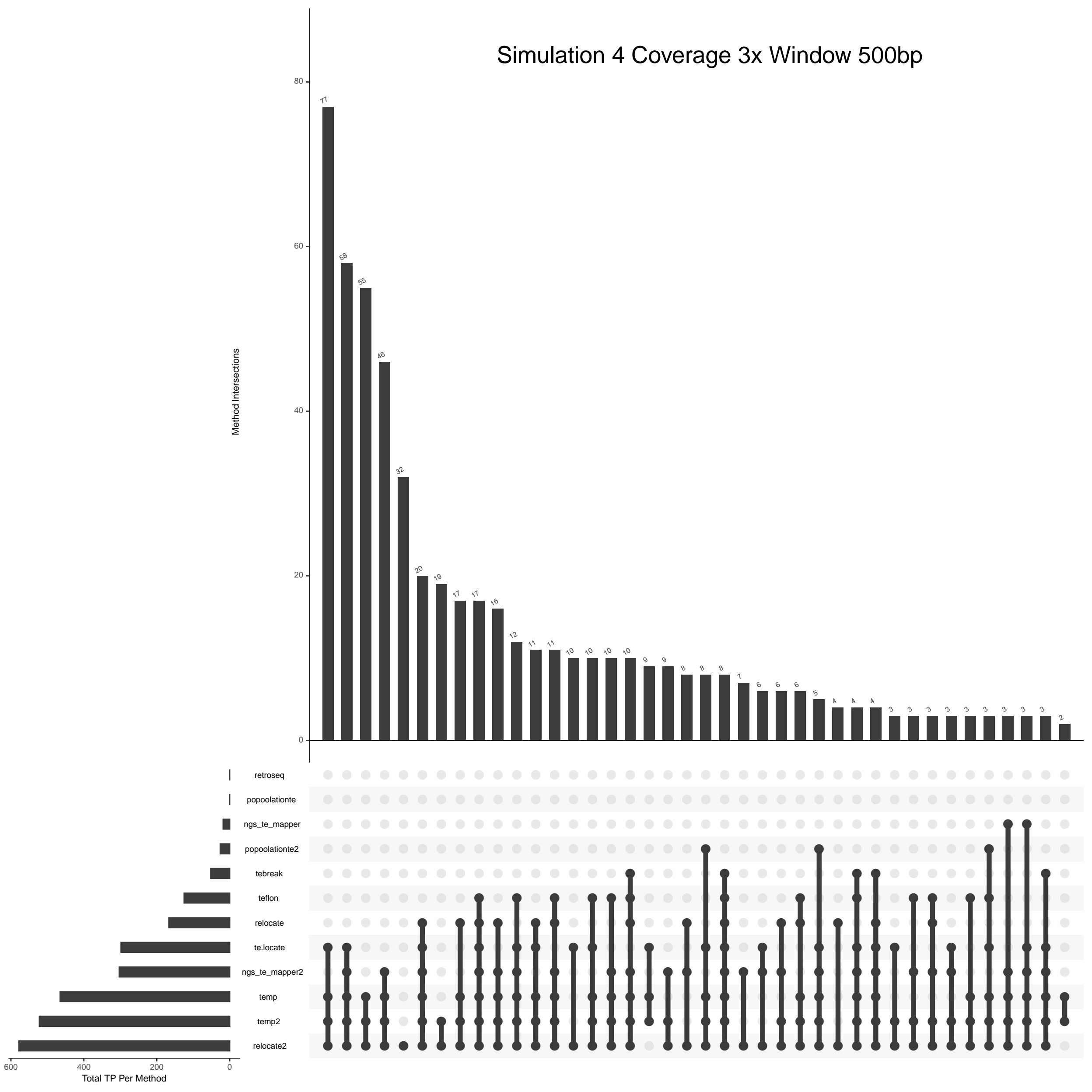

Supplement: Supplementary file 4 — Additional file 4. Overlaps between numbers of non-reference TEs predicted by McClintock component methods in simulated data. UpSet plots visualizing overlaps among component methods for true positive predictions at different window sizes and fold-coverages for Simulations 3 and 4. [file 13100_2023_296_MOESM4_ESM.zip › intersection/sim4_upsetplot_3x_500.pdf]

### Simulation 3 Coverage 6 Window 500

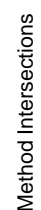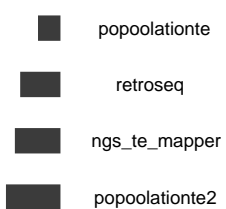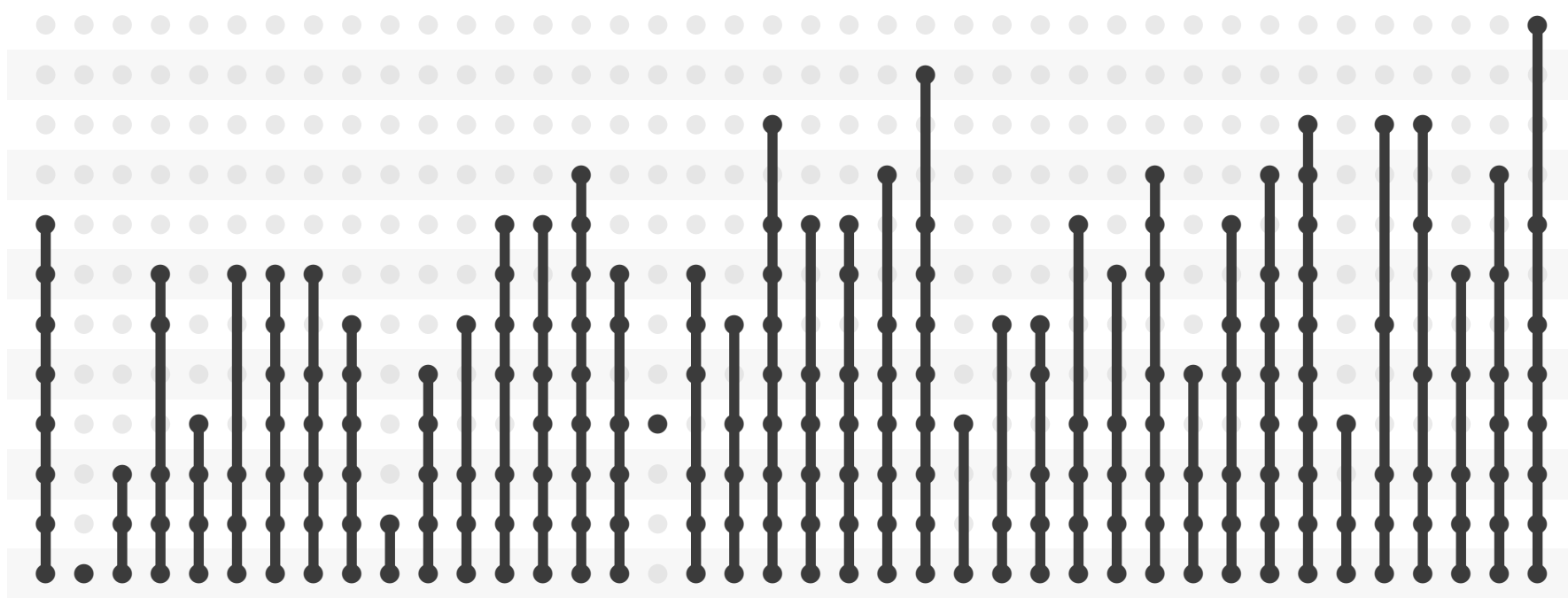

Supplement: Supplementary file 4 — Additional file 4. Overlaps between numbers of non-reference TEs predicted by McClintock component methods in simulated data. UpSet plots visualizing overlaps among component methods for true positive predictions at different window sizes and fold-coverages for Simulations 3 and 4. [file 13100_2023_296_MOESM4_ESM.zip › intersection/sim3_upsetplot_6x_500.pdf]

Simulation 4 Coverage 50x Window 500bp

Method Intersections

150  
100  
50  
0

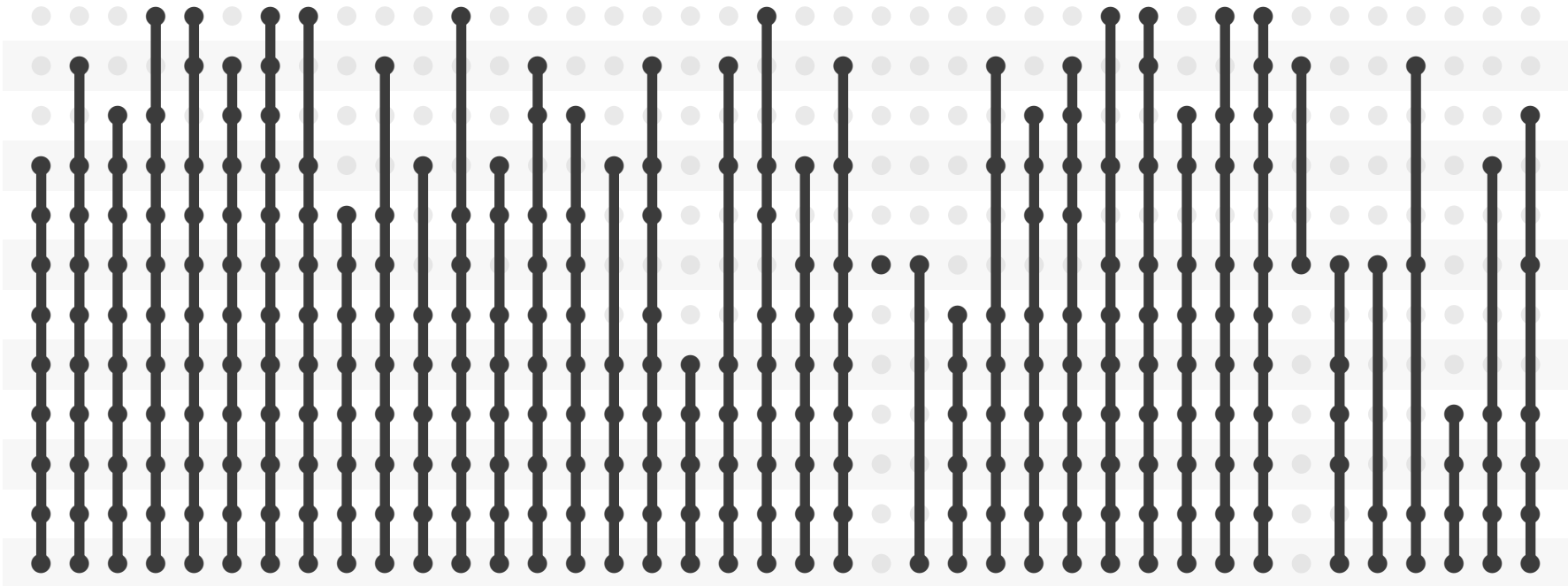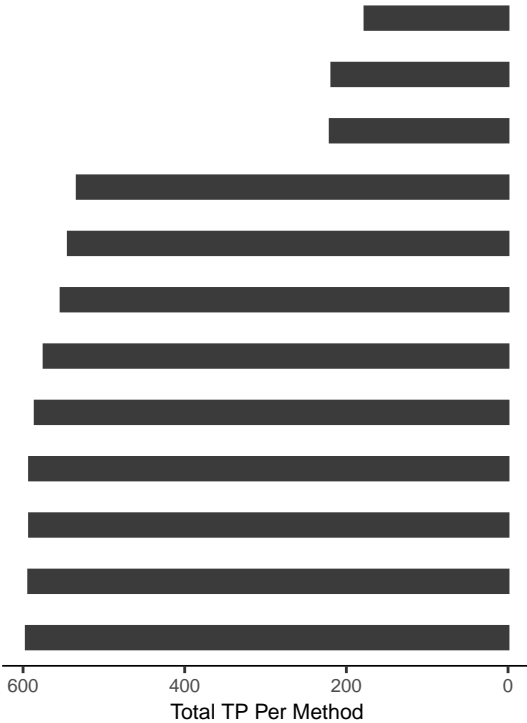

Supplement: Supplementary file 4 — Additional file 4. Overlaps between numbers of non-reference TEs predicted by McClintock component methods in simulated data. UpSet plots visualizing overlaps among component methods for true positive predictions at different window sizes and fold-coverages for Simulations 3 and 4. [file 13100_2023_296_MOESM4_ESM.zip › intersection/sim4_upsetplot_50x_500.pdf]

Simulation 4 Coverage 50x Window 0bp

Method Intersections

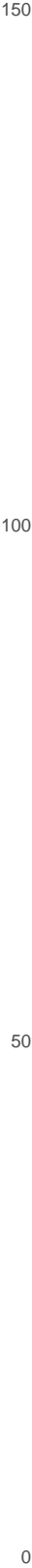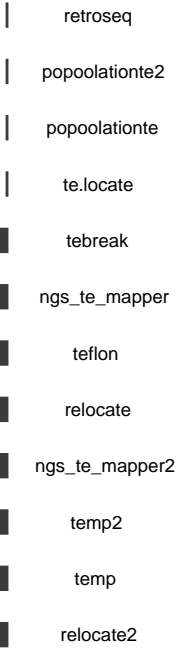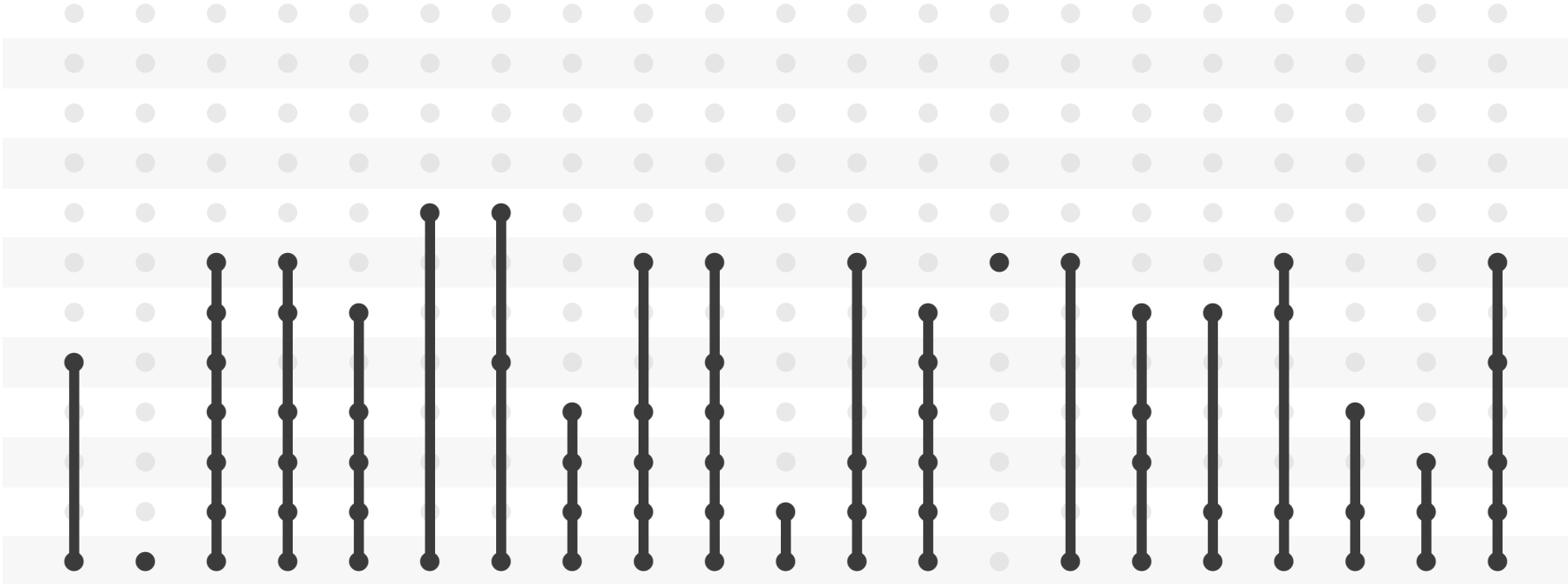

Supplement: Supplementary file 4 — Additional file 4. Overlaps between numbers of non-reference TEs predicted by McClintock component methods in simulated data. UpSet plots visualizing overlaps among component methods for true positive predictions at different window sizes and fold-coverages for Simulations 3 and 4. [file 13100_2023_296_MOESM4_ESM.zip › intersection/sim4_upsetplot_50x_0.pdf]

Simulation 3 Coverage 3 Window 5

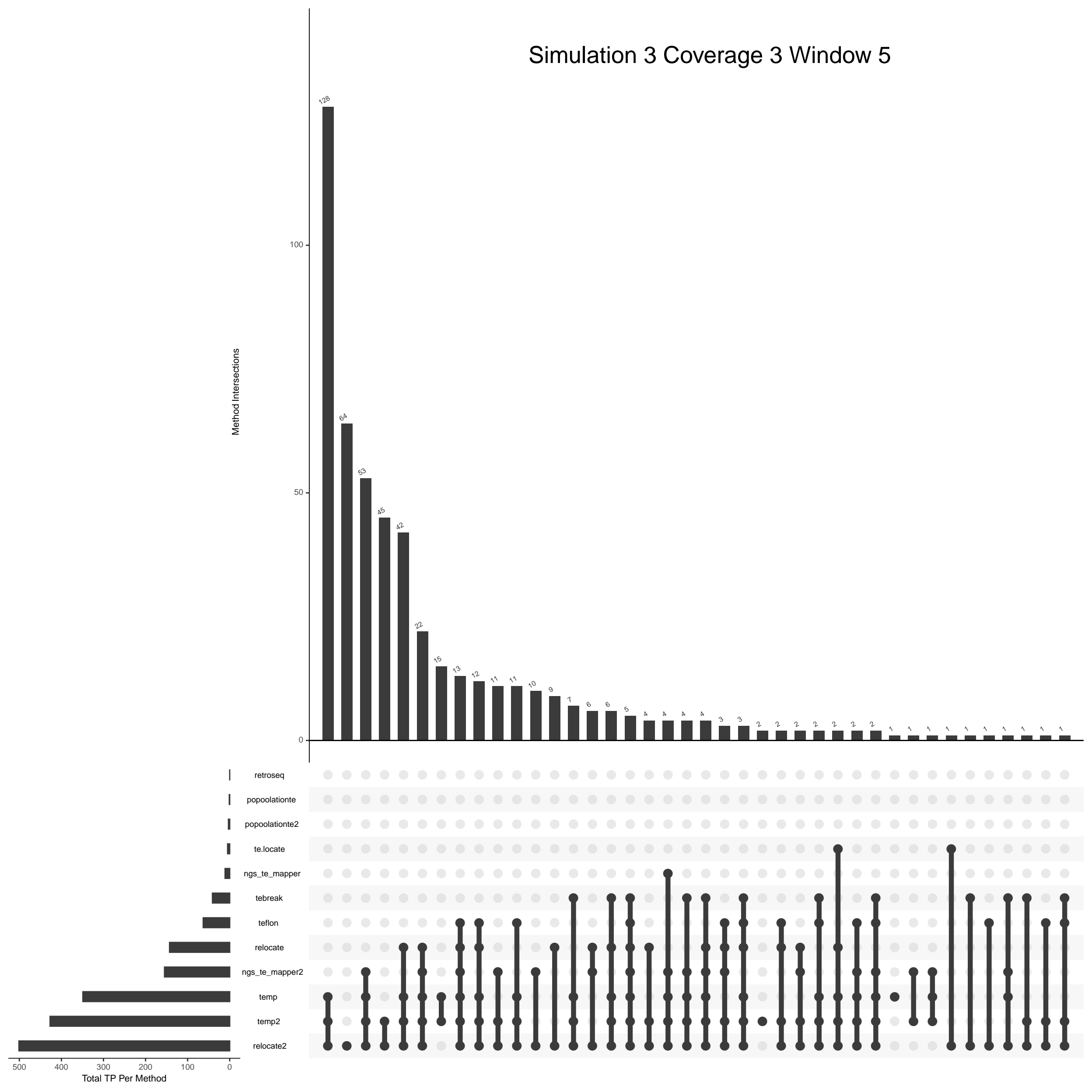

Supplement: Supplementary file 4 — Additional file 4. Overlaps between numbers of non-reference TEs predicted by McClintock component methods in simulated data. UpSet plots visualizing overlaps among component methods for true positive predictions at different window sizes and fold-coverages for Simulations 3 and 4. [file 13100_2023_296_MOESM4_ESM.zip › intersection/sim3_upsetplot_3x_5.pdf]

## Simulation 4 Coverage 50x Window 100bp

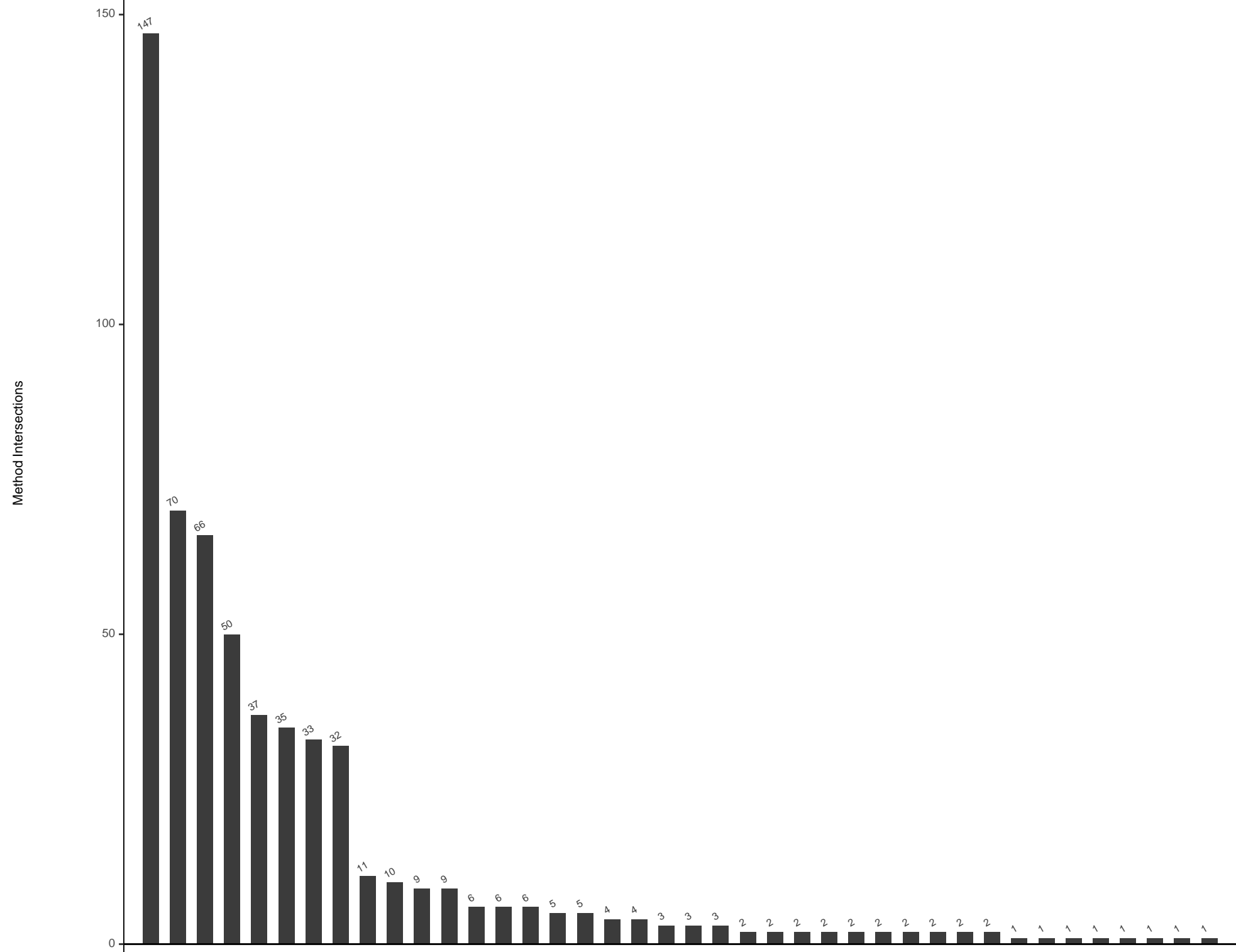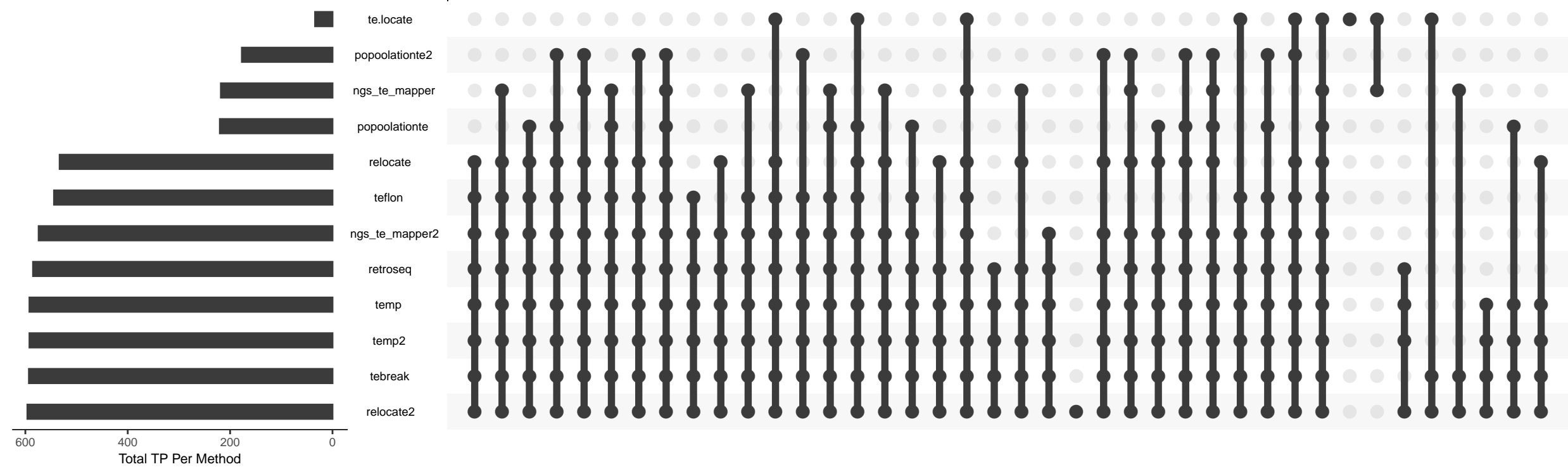

Supplement: Supplementary file 4 — Additional file 4. Overlaps between numbers of non-reference TEs predicted by McClintock component methods in simulated data. UpSet plots visualizing overlaps among component methods for true positive predictions at different window sizes and fold-coverages for Simulations 3 and 4. [file 13100_2023_296_MOESM4_ESM.zip › intersection/sim4_upsetplot_50x_100.pdf]

### Simulation 3 Coverage 6 Window 300

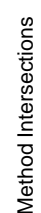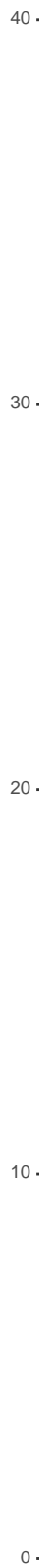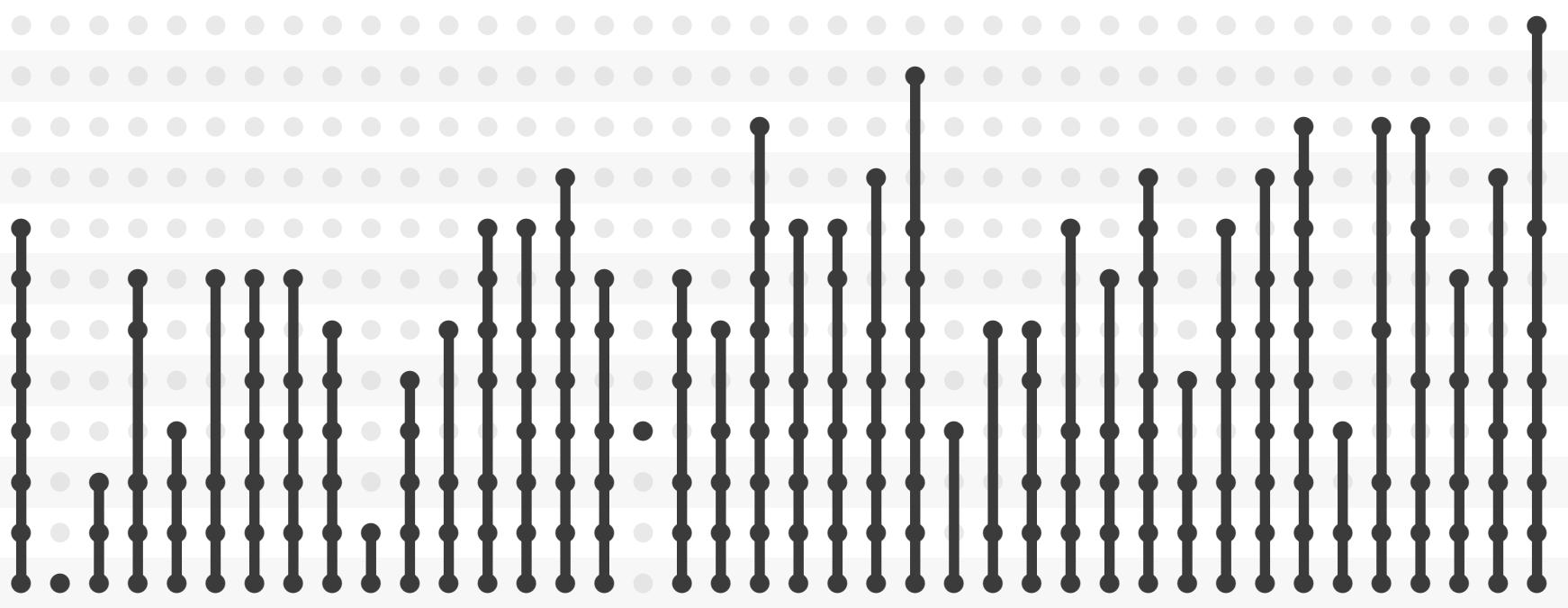

Supplement: Supplementary file 4 — Additional file 4. Overlaps between numbers of non-reference TEs predicted by McClintock component methods in simulated data. UpSet plots visualizing overlaps among component methods for true positive predictions at different window sizes and fold-coverages for Simulations 3 and 4. [file 13100_2023_296_MOESM4_ESM.zip › intersection/sim3_upsetplot_6x_300.pdf]

Simulation 4 Coverage 50x Window 5bp

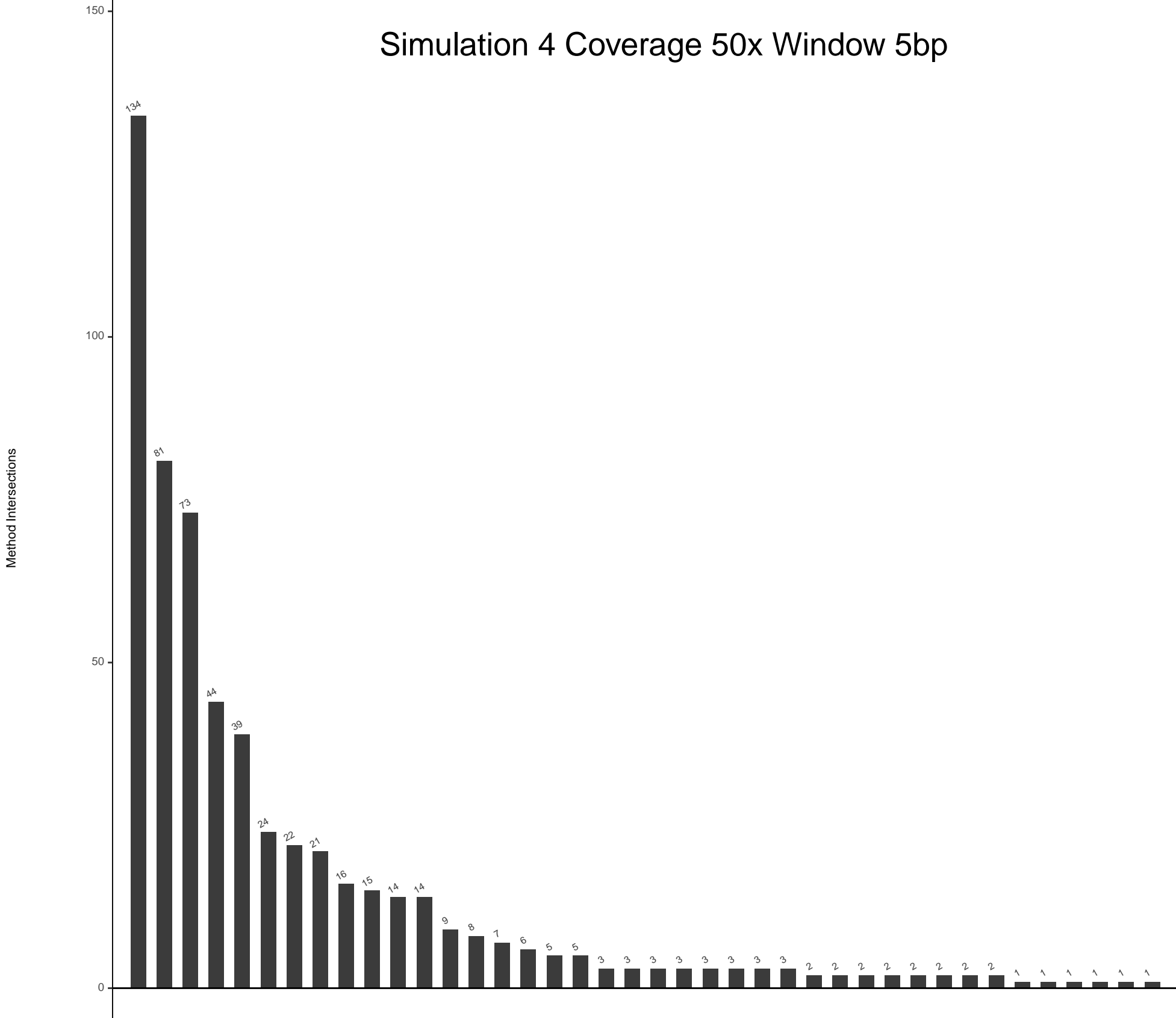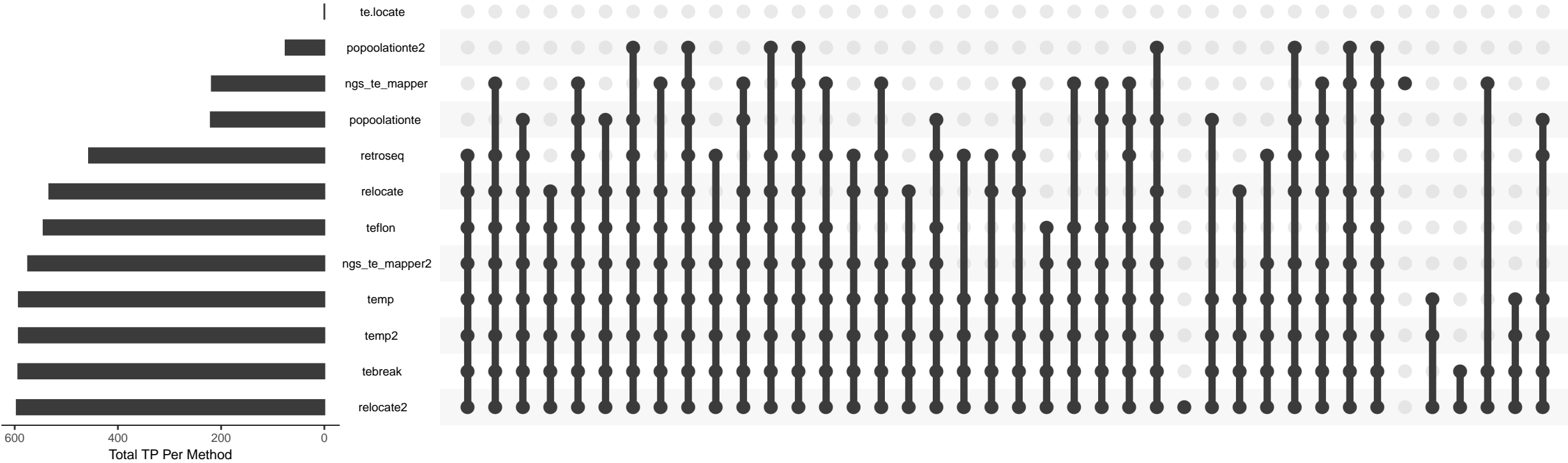

Supplement: Supplementary file 4 — Additional file 4. Overlaps between numbers of non-reference TEs predicted by McClintock component methods in simulated data. UpSet plots visualizing overlaps among component methods for true positive predictions at different window sizes and fold-coverages for Simulations 3 and 4. [file 13100_2023_296_MOESM4_ESM.zip › intersection/sim4_upsetplot_50x_5.pdf]

Simulation 3 Coverage 50 Window 0

Method Intersections

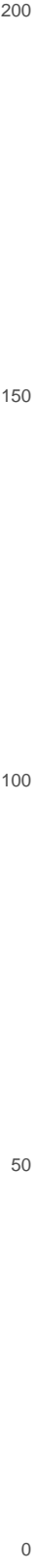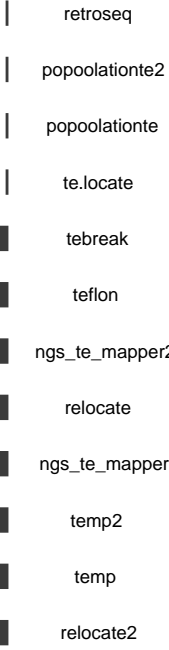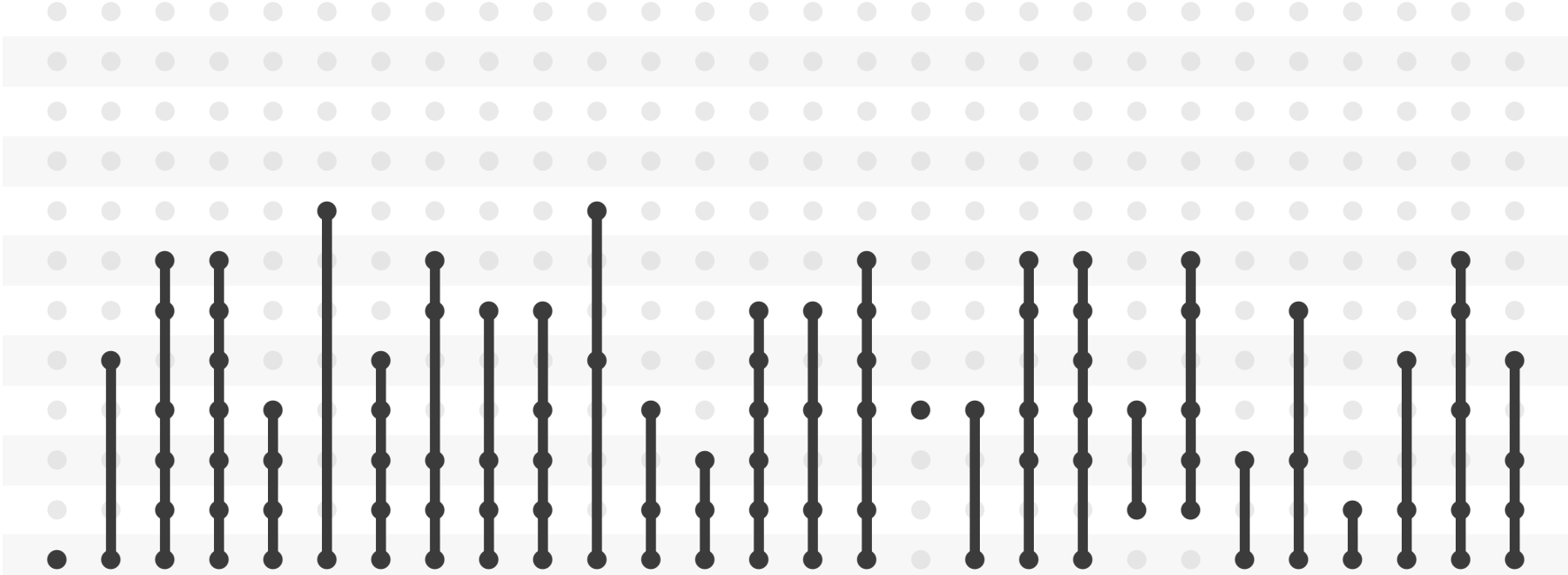

Supplement: Supplementary file 4 — Additional file 4. Overlaps between numbers of non-reference TEs predicted by McClintock component methods in simulated data. UpSet plots visualizing overlaps among component methods for true positive predictions at different window sizes and fold-coverages for Simulations 3 and 4. [file 13100_2023_296_MOESM4_ESM.zip › intersection/sim3_upsetplot_50x_0.pdf]

### Simulation 3 Coverage 3 Window 0

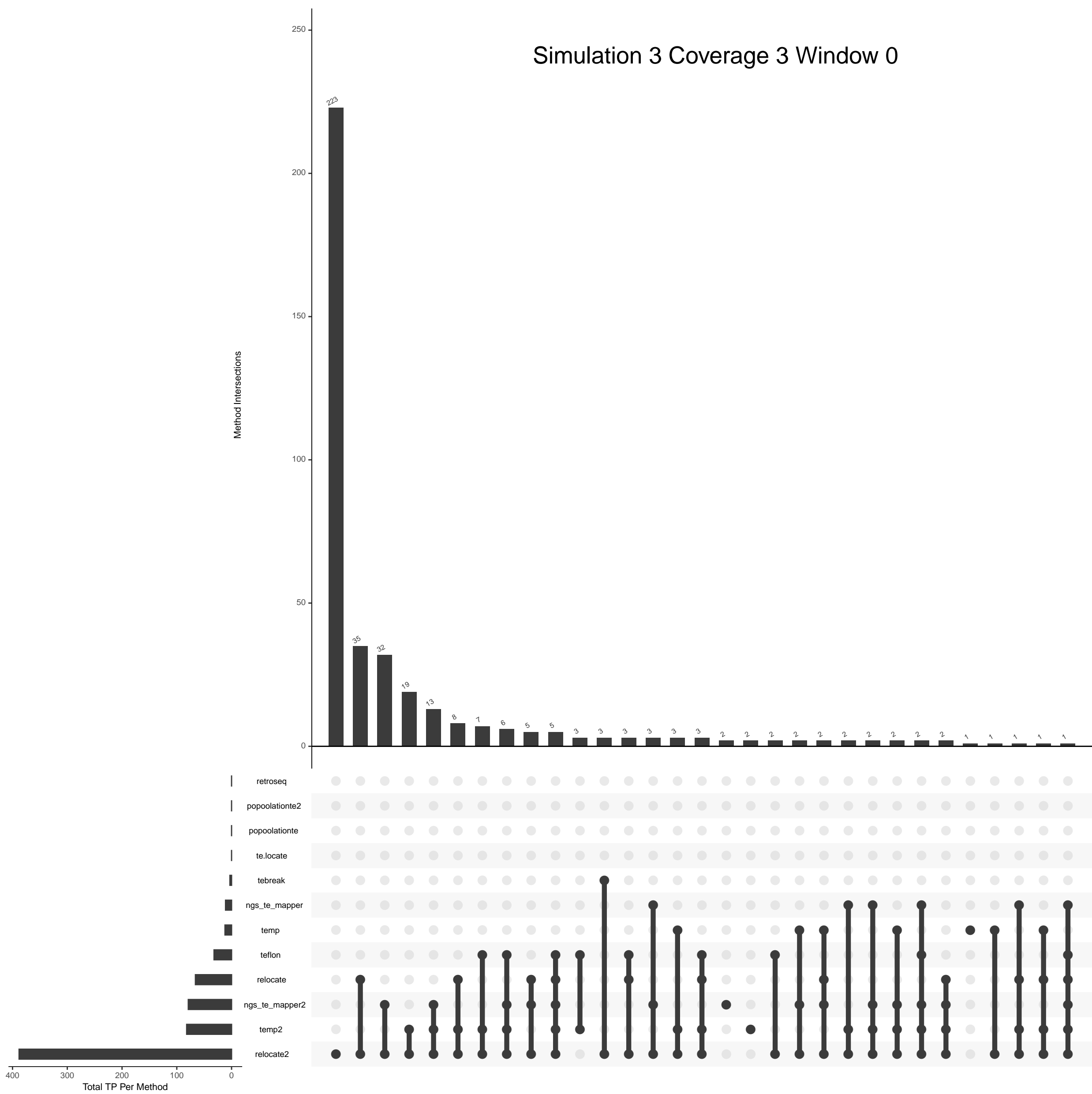

Supplement: Supplementary file 4 — Additional file 4. Overlaps between numbers of non-reference TEs predicted by McClintock component methods in simulated data. UpSet plots visualizing overlaps among component methods for true positive predictions at different window sizes and fold-coverages for Simulations 3 and 4. [file 13100_2023_296_MOESM4_ESM.zip › intersection/sim3_upsetplot_3x_0.pdf]
