# Supplementary material for: Reproducible evaluation of transposable element detectors with McClintock 2 guides accurate inference of Ty insertion patterns in yeast
Source: Mob DNA. 2023 Jul 14;14:8. doi: 10.1186/s13100-023-00296-4 (PMC10347736; doi:10.1186/s13100-023-00296-4)
Supplement: Supplementary file 4 — Additional file 4. Overlaps between numbers of non-reference TEs predicted by McClintock component methods in simulated data. UpSet plots visualizing overlaps among component methods for true positive predictions at different window sizes and fold-coverages for Simulations 3 and 4. [file sim3_upsetplot_100x_0.pdf › intersection/sim3_upsetplot_100x_0.pdf]

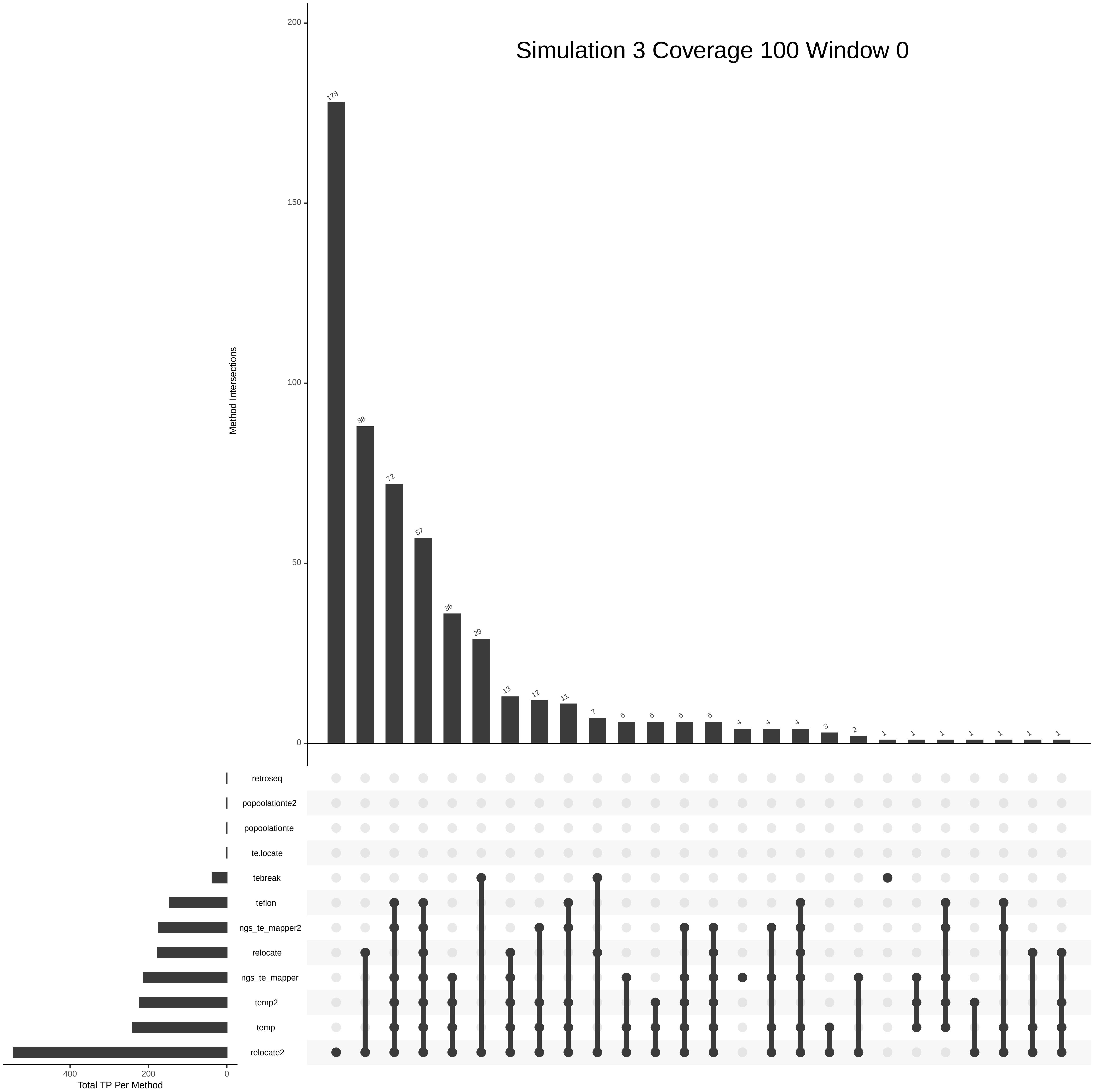

Simulation 3 Coverage 100 Window 0
Method Intersections
0
50
100
150
200
178
88
72
57
36
29
13
12
11
7
6
6
6
6
4
4
4
3
2
1
1
1
1
1
1
1
retroseq
popoolationte2
popoolationte
te.locate
tebreak
teflon
ngs_te_mapper2
relocate
ngs_te_mapper
temp2
temp
relocate2
400
200
0
Total TP Per Method
